# Supplementary material for: Bacterial ubiquitin ligase engineered for small molecule and protein target identification
Source: EMBO J. 2026 Jan 3;45(3):1024–50. doi: 10.1038/s44318-025-00665-0 (PMC12865202; doi:10.1038/s44318-025-00665-0)
Supplement: Supplementary file 1 — Appendix [file 44318_2025_665_MOESM1_ESM.pdf]

Appendix for *Bacterial ubiquitin ligase engineered for small molecule and protein target identification*

Table of contents

Appendix Table S1: Data collection and refinement statistics for crystal structures.

Appendix Table S2: Primers used in this study.

Appendix Supplementary Methods: Synthesis of CLP-linker and CLP-small molecules.

Appendix Table S1. Data collection and refinement statistics for CAMKIIδ^{kd}:ribociclib and RavB:CapZ structures.

Values in parentheses are for highest resolution shell.

	**CAMKIIδ^{kd}:ribociclib**	**RavB:CapZ**
**Data collection**		
Space group	$P2_12_12_1$	$P2_1$
Cell dimensions		
a, b, c (Å)	46.32, 82.82, 172.91	66.50, 55.32, 77.90
α, β, γ (°)	90, 90, 90	90, 107.08, 90
Wavelength (Å)	0.97946	1.5418
Resolution range (Å)	39.37 - 2.35 (2.39 - 2.35)	44.41 - 2.00 (2.03 - 2.00)
Unique reflections	28133 (1168)	35564 (1036)
Multiplicity	6.9 (4.9)	9.3 (1.3)
Completeness (%)	98.4 (82.6)	96.7 (56.6)
R_{merge} (%)	6.1 (79.1)	12.4 (35.9)
R_{pim} (%)	2.4 (35.7)	3.9 (34.5)
$I/\sigma I$	28.9 (1.6)	19.3 (1.8)
$CC_{1/2}$	0.74	0.79
Wilson B-value (Å^2)	32.7	25.5
**Refinement**		
Resolution range (Å)	39.37 - 2.35 (2.41 - 2.35)	44.41 - 2.00 (2.04 - 2.00)
No. of reflections $R_{\mathrm{work}}/R_{\mathrm{free}}$	26175/1900 (823/67)	35046/2550 (769/61)
Data completeness (%)	91.7 (44.0)	95.21 (41.0)
Non-hydrogen atoms	4788	4585
protein	4641	4404
ligand	64	NA
solvent	73	181
R_{work}	0.197 (0.251)	0.180 (0.263)
R_{free}	0.251 (0.339)	0.208 (0.362)
R.m.s.d.		
Bond length (Å)	0.002	0.005
Bond angle (°)	0.558	0.712
Mean B-value		
chain A	41.2	31.6
chain B	52.3	27.0
chain C	NA	33.6
ligands	43.6	NA
solvent	33.5	28.4
Ramachandran favored (%)	96.9	97.2
Ramachandran allowed (%)	2.9	2.8
Ramachandran outliers (%)	0.2	0.0
Clashscore	2.1	1.5
Maximum likelihood coordinate error	0.29	0.20
Missing residues		
chain A	10, 302-309	1,2, 277-286
chain B	10, 297-309	NA
chain C	NA	109-114, 140-304

Appendix Table S2. Primer sequences used in this study.

Name		Sequence		Purpose
pETDuet1_BirA_F_NcoI		AAAACCATGGGCATGAAGGATAACACCGTGCCACTG		clone E. coli BirA into pETDuet1
pETDuet1_BIrA_R_BamHI		AAAAGGATCCTTATTTTTCTGCACTACGCAGGGATATTTCAC		
SdeA_codopt_519-1100_BamH1_F		AAAAGCGGATCCCTTCCTCAAGCTCCCAGAGATTC		clone the codon optimized SdeA ART domain into petduet1-BirA; cut vector with BglII and Xho1
SdeA_codopt_519-1100_Xho1_F		AAAACTCGAGCTACCGCTCCCTTACCTTTTTTCATC		
SdeA178-1100_BamH1_F		AAAAGGATCCGGTGACCCACAATTAGATGGGAAAG		clone SdeA 178-1100 from lp02 gDNA into ppSumo
SdeA178-1100_Xho1_R		AAAACTCGAGCTAGCGTTCTCTTACTTTCTTTTCATCG		
SNAPf_C145A_F		CTGAACCACGCGATGAGCCGGAATCAGAATCGGG		generate the SNAPf C145A mutant
SNAPf_C145A_R		CCCGATTCTGATTCCGGCTCATCGCGTGGTTCAG		
EaHopBF1_EcoR1_F		AAAAGAATTCATGTTCAATGTCTCTAACAATGTCGC		clone *E. Americana* HopBF1 into the pET28a-SidBait vector
EaHopBF1_Xho1_R		AAAATGCTCGAGTATCGCTATCAATATCCAGCAGGTTATTG		
RavB_BamH1_F		AAAAGGATCCATGAAAACAGAAGCCTTACTTAGCTG		clone RavB from lp02 gDNA into the pET28a-SidBait vector
RavB_Xho1_R		AAAATGCTCGAGTTTTAAAAAATTAACAGTTGAGTTTTGTGCTATC		
ravB_BamH1_F		AAAAGGATCCATGAAAACAGAAGCCTTACTTAGCTGG		clone RavB from lp02 gDNA into ppSumo
ravB_Xho1_R		AAAACTCGAGTCATTTTAAAAAATTAACAGTTGAGTTTTGTGC		
ravB_EcoR1_F		AAAATCGAATTCATGAAAACAGAAGCCTTACTTAGCTGG		clone RavB from lp02 gDNA into pEGFP-C1
ravB_BamH1_R		AAAAGGATCCTCATTTTAAAAAATTAACAGTTGAGTTTTGTGC		
KCC2D_BamHI_F		TAAGCAGGATCCACGGACGAGTATCAGCTTTTCGA		clone the CaMKIIδ kinase domain into ppSumo
KCC2D_XhoI_R		TAAGCACTCGAGTCACAGCATAGTTGTCAAGATGGCACC		
FCGR2B_EcoR1_F		AAAAGAATTCACCATGGGAATCCTGTCATTCTTACCTG		clone FcγRIIB into pCDNA3.1
FCGR2B_Xba1_R		AAAATCTAGAAATACGGTTCTGGTCATCAGGC		
RavB_CTD_145-C_EcoRI_F		AAAAGAATTCTACTGAAGGGGCCGAACAACAAATTAATG		clone the C-terminal domain of RavB into pEGFP
RavB_CTD_145-C_BamHI_R		TTTTGGATCCTCATTTTAAAAAATTAACAGTTGAGTTTTGTGCTATC		
RavB_R129A_130A_F		CACAAGGCCCTCGGGGAGCAGCCTTACCCGCCCATTTAC		generate the RavB R129A+R130A mutant

RavB_R129A_130A_R		GTAAATGGGCGGGTAAGGCTGCTCCCCGAGGGCCTTGTG		
RavB_R122A_F		ATTAACCCACTTTGTAAGAGAAGCGGCACAAGGCCCTC		generate the RavB R122A mutant
RavB_R122A_R		GAGGGCCTTGTGCCGCTTCTCTTACAAAGTGGGTTAAT		
RavB_H214A_F		CTGTAGTGATTCCAAAGCTTCCAGCGCGAAAAAAACTGAAAAAACCAT		generate the RavB H214A mutant
RavB_H214A_R		ATGGTTTTTTCAGTTTTTTTCGCGCTGGAAGCTTTGGAATCACTACAG		

Appendix Supplementary Methods. Synthesis of CLP-linker and CLP-small molecules.

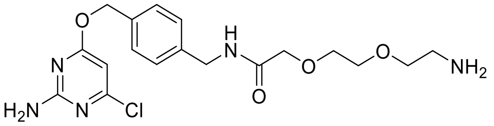

4

N-(4-(((2-amino-6-chloropyrimidin-4-yl)oxy)methyl)benzyl)-2-(2-(2-aminoethoxy)ethoxy)acetamide

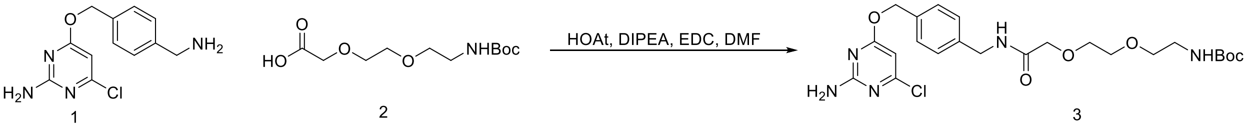

tert-butyl (2-(2-(2-((4-(((2-amino-6-chloropyrimidin-4-yl)oxy)methyl)benzyl)amino)-2-oxoethoxy)ethoxy)ethyl)carbamate. A solution of CLP-amine **1** (100.6 mg, 0.38 mmol) and 2,2-dimethyl-4-oxo-3,8,11-trioxa-5-azatridecan-13-oic acid **2** (119.5 mg, 0.45 mmol) in anhydrous DMF (1.9 ml) was cooled in an ice bath before the addition of 1-hydroxy-7-azabenzotriazole HOAt (71.0 mg, 0.52 mmol) and N,N-diisopropylethylamine DIPEA (198 µl, 1.1 mmol). The reaction was stirred cold for 5 minutes, after which N-(3-Dimethylaminopropyl)-N′-ethylcarbodiimide hydrochloride EDC (101.8 mg, 0.53 mmol) was added and the reaction was gently warmed to ambient temperature and stirred overnight. The reaction was diluted with EtOAc and washed with saturated $NaHCO_3$ and brine. The organic layer was dried over Na_2SO_4, filtered and condensed. Crude reaction product was carried forward directly. ^{1}H NMR (400 MHz, Chloroform-*d*) δ 7.36 (d, J = 8.1 Hz, 2H), 7.31 (d, J = 8.2 Hz, 2H), 7.22 (bs, 1H), 6.16 (s, 1H), 5.30 (d, J = 6.2 Hz, 4H), 5.14 (s, 2H), 4.71 (s, 1H), 4.49 (d, J = 5.9 Hz, 2H), 4.05 (s, 2H), 3.69 – 3.64 (m, 2H), 3.60 – 3.54 (m, 2H), 3.41 (t, J = 5.3 Hz, 2H), 3.14 (q, J = 5.4 Hz, 2H), 1.43 (s, 9H). ESI-MS (*m/z*): 510.1[M+H]$^+$

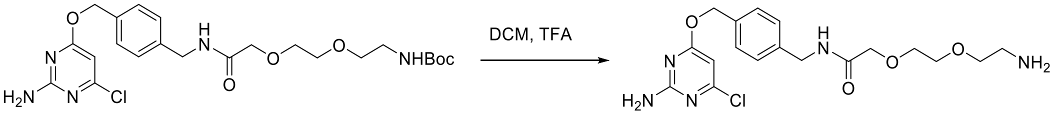

A solution of tert-butyl (2-(2-(2-((4-(((2-amino-6-chloropyrimidin-4-yl)oxy)methyl)benzyl)amino)-2-oxoethoxy)ethoxy)ethyl)carbamate **3** (156.3 mg, 0.31 mmol) in anhydrous DCM (2.5 ml) was cooled in an ice bath before the slow dropwise addition of trifluoroactic acid TFA (1.1 ml, 14.4 mmol). Lcms monitoring after 1 hour showed complete consumption of SM and desired m/z. The reaction was condensed and crude reaction mixture was carried forward. ^{1}H NMR (400 MHz, Chloroform-*d*) δ 7.39 – 7.28 (m, 6H), 7.17 (dd, J = 7.9, 3.2 Hz, 1H), 6.16 (d, J = 0.9 Hz, 1H), 5.31 (s, 2H), 5.15 (s, 2H), 4.50 (d, J = 6.0 Hz, 3H), 4.06 (d, J = 2.1 Hz, 2H), 3.71 – 3.66 (m, 3H), 3.62 – 3.57 (m, 2H), 3.42 (t, J = 5.3 Hz, 2H), 2.73 (t, J = 5.3 Hz, 2H), 2.35 (s, 1H). ESI-MS (*m/z*): 410.1 [M+H]$^+$

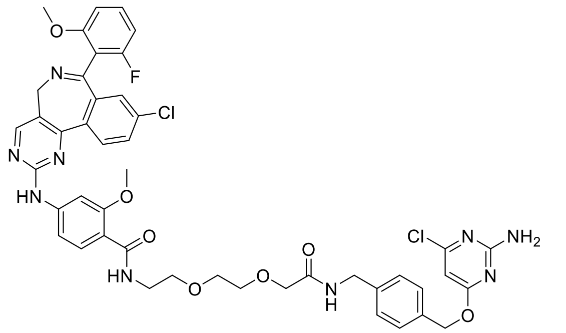

N-(2-(2-(2-((4-(((2-amino-6-chloropyrimidin-4-yl)oxy)methyl)benzyl)amino)-2-oxoethoxy)ethoxy)ethyl)-4-((9-chloro-7-(2-fluoro-6-methoxyphenyl)-5H-benzo[c]pyrimido[4,5-e]azepin-2-yl)amino)-2-methoxybenzamide

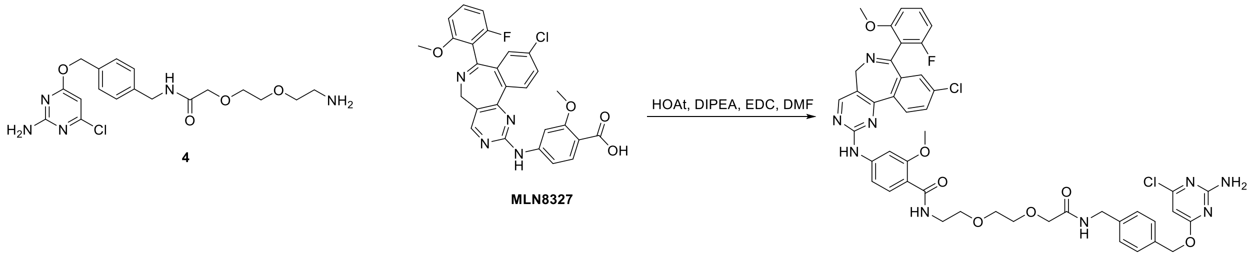

A solution of **4** (52.0 mg, 0.13 mmol) and MLN8237 (69.7 mg, 0.13 mmol) in anhydrous DMF (0.61 ml) was cooled in an ice bath before the addition of 1-hydroxy-7-azabenzotriazole (23.5 mg, 0.17 mmol) and N,N-diisopropylethylamine (64 µl, 0.37 mmol). The reaction was stirred cold for 5 minutes, after which N-(3-Dimethylaminopropyl)-N′-ethylcarbodiimide hydrochloride (34.8 mg, 0.18 mmol) was added and the reaction was gently warmed to ambient temperature and stirred overnight. Lcms monitoring indicated complete consumption of the amine and the desired m/z as the major product, along with a major and a minor byproduct. Both were less polar and smaller m/z than the desired. The reaction was diluted with EtOAc and washed with saturated $NaHCO_3$ and brine. The organic layer with suspended solid was filtered. The isolated solid was mostly both by-products and about 20% of the desired. Most of the desired was in the filtrate of the organic layer, which was dried over Na_2SO_4, filtered and condensed. The crude mixture was purified by ISCO flash column chromatography in 0-15% MeOH/DCM to give 35.0 mg of pure product. Y=29 %. ^{1}H NMR (400 MHz, Chloroform-*d*) δ 8.46 (s, 1H), 8.15 (d, *J* = 8.4 Hz, 1H), 8.08 (d, *J* = 8.6 Hz, 1H), 8.04 (t, *J* = 5.3 Hz, 1H), 7.96 (s, 1H), 7.85 (d, *J* = 2.0 Hz, 1H), 7.51 (dd, *J* = 8.4, 2.2 Hz, 1H), 7.40 (t, *J* = 6.0 Hz, 1H), 7.33 – 7.21 (m, 3H), 7.06 (d, *J* = 8.7 Hz, 1H), 6.65 (d, *J* = 59.5 Hz, 2H), 6.04 (s, 1H), 5.46 (s, 2H), 5.13 (s, 2H), 4.89 (d, *J* = 42.0 Hz, 1H), 4.40 (d, *J* = 6.0 Hz, 2H), 4.04 (s, 3H), 3.83 (s, 4H), 3.72 – 3.58 (m, 5H), 3.52 (dq, *J* = 14.3, 4.8 Hz, 5H). ESI-MS (*m/z*): 910.1 $[M+H]^+$

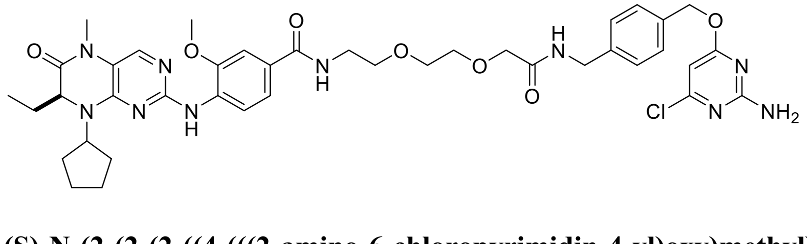

(S)-N-(2-(2-(2-((4-(((2-amino-6-chloropyrimidin-4-yl)oxy)methyl)benzyl)amino)-2-oxoethoxy)ethoxy)ethyl)-4-((8-cyclopentyl-7-ethyl-5-methyl-6-oxo-5,6,7,8-tetrahydropteridin-2-yl)amino)-3-methoxybenzamide

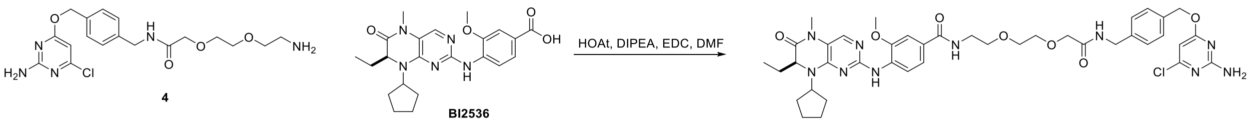

An emulsion of **4** (48.3 mg, 0.12 mmol) and BI2536 (55.5 mg, 0.13 mmol) in anhydrous DMF was cooled in an ice bath before the addition of 1-hydroxy-7-azabenzotriazole (21.4 mg, 0.16 mmol) and N,N-diisopropylethylamine (62 µl, 0.36 mmol). The reaction was stirred cold for 5 minutes, after which N-(3-Dimethylaminopropyl)-N′-ethylcarbodiimide hydrochloride (33.1 mg, 0.17 mmol) was added and the reaction was gently warmed to ambient temperature and stirred overnight in the absence of light. The reaction was diluted with EtOAc and washed with saturated $NaHCO_3$ and brine. The organic layer was dried over Na_2SO_4, filtered and condensed. The crude mixture was purified by ISCO flash column chromatography in 0-10% MeOH/DCM to give 61.1 mg of pure product. Isolated yield=63 %. ^{1}H NMR (400 MHz, Chloroform-*d*) δ 8.51 (d, *J* = 8.4 Hz, 1H), 7.65 (s, 1H), 7.58 (s, 1H), 7.39 (d, *J* = 1.9 Hz, 1H), 7.24 (q, *J* = 8.2 Hz, 6H), 6.56 (t, *J* = 5.5 Hz, 1H), 6.07 (s, 1H), 5.46 – 5.38 (m, 2H), 5.20 (s, 2H), 4.51 – 4.43 (m, 1H), 4.41 (d, *J* = 5.9 Hz, 2H), 4.19 (dd, *J* = 7.9, 3.7 Hz, 1H), 4.04 (s, 2H), 3.89 (s, 3H), 3.69 – 3.64 (m, 2H), 3.63 – 3.58 (m, 2H), 3.55 (t, *J* = 5.0 Hz, 2H), 3.49 (q, *J* = 6.0, 5.5 Hz, 2H), 3.43 (s, 1H), 3.29 (s, 3H), 2.18 – 2.04 (m, 2H), 1.97 (dq, *J* = 12.4, 8.7, 5.9 Hz, 1H), 1.88 – 1.73 (m, 4H), 1.73 – 1.59 (m, 4H), 0.84 (t, *J* = 7.5 Hz, 3H).). ESI-MS (*m/z*): 817.1 $[M+H]^+$
