# Supplementary figures and images for: Bacterial ubiquitin ligase engineered for small molecule and protein target identification
Source: EMBO J. 2026 Jan 3;45(3):1024–50. doi: 10.1038/s44318-025-00665-0 (PMC12865202; doi:10.1038/s44318-025-00665-0)

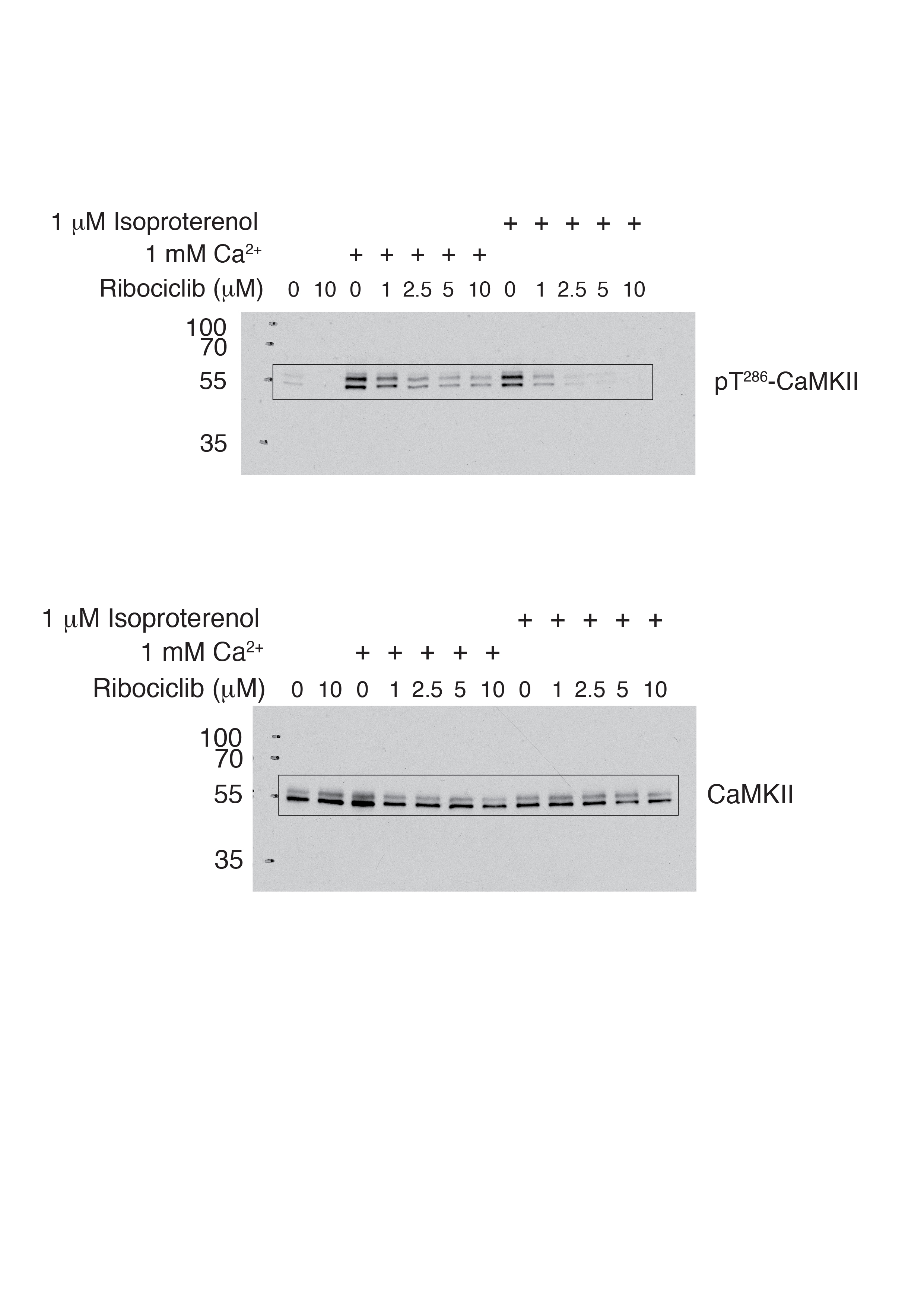

Supplement: Supplementary file 12 — Source data Fig. 2 [file 44318_2025_665_MOESM12_ESM.zip › Figure 2/2c.jpg]

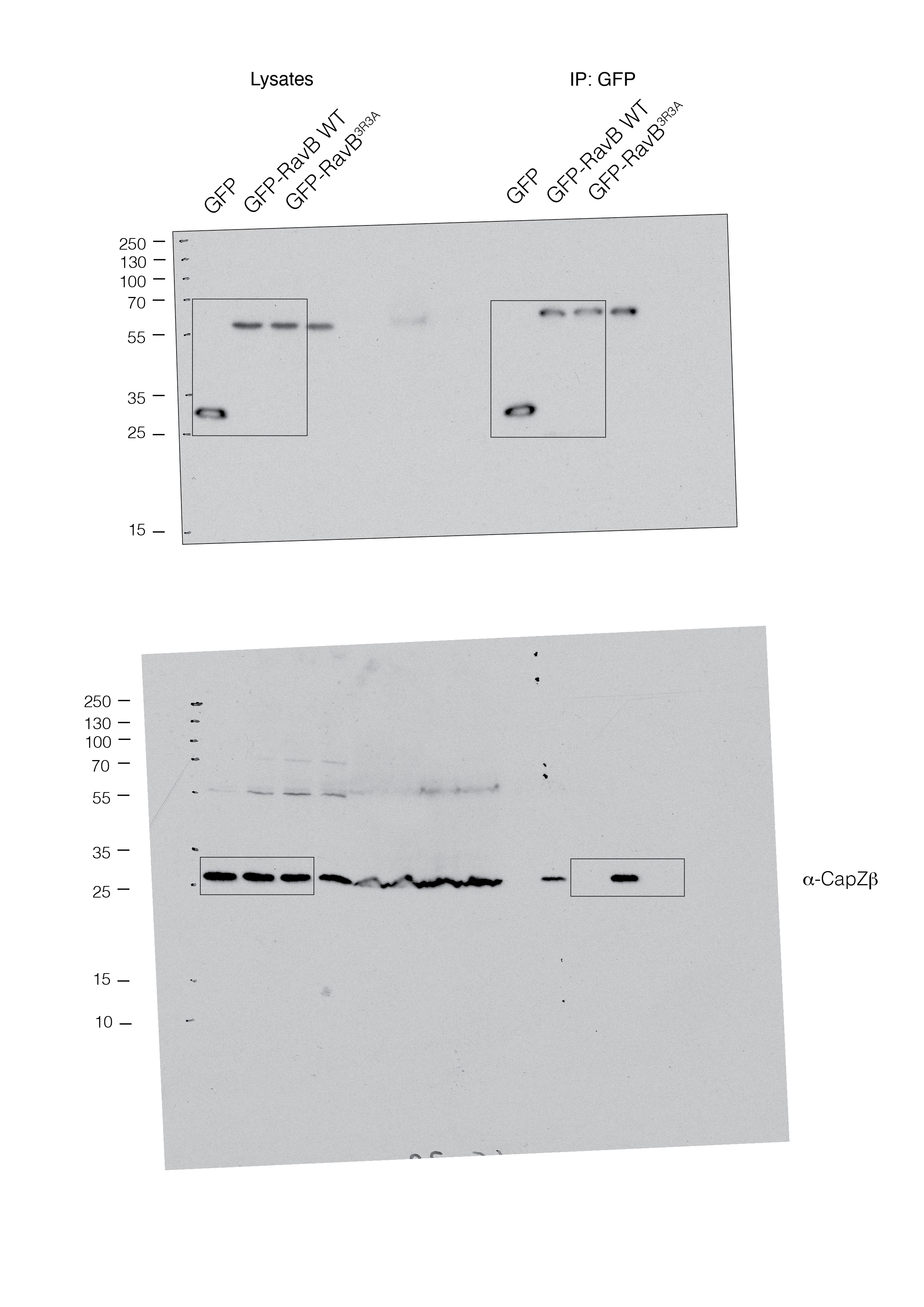

Supplement: Supplementary file 13 — Source data Fig. 4 [file 44318_2025_665_MOESM13_ESM.zip › Figure 4/4g.jpg]

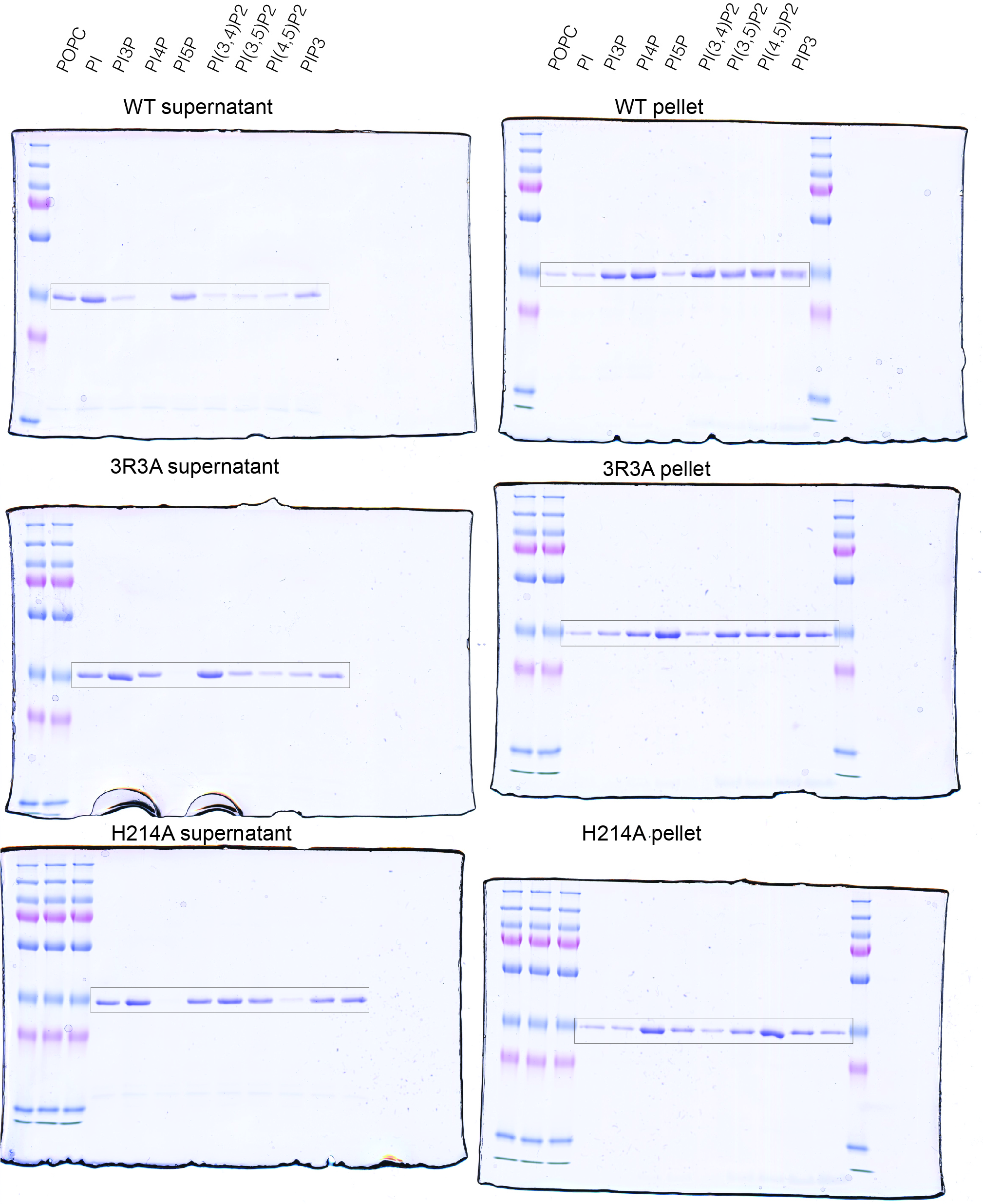

Supplement: Supplementary file 14 — Source data Fig. 5 [file 44318_2025_665_MOESM14_ESM.zip › Figure 5/5c.jpg]

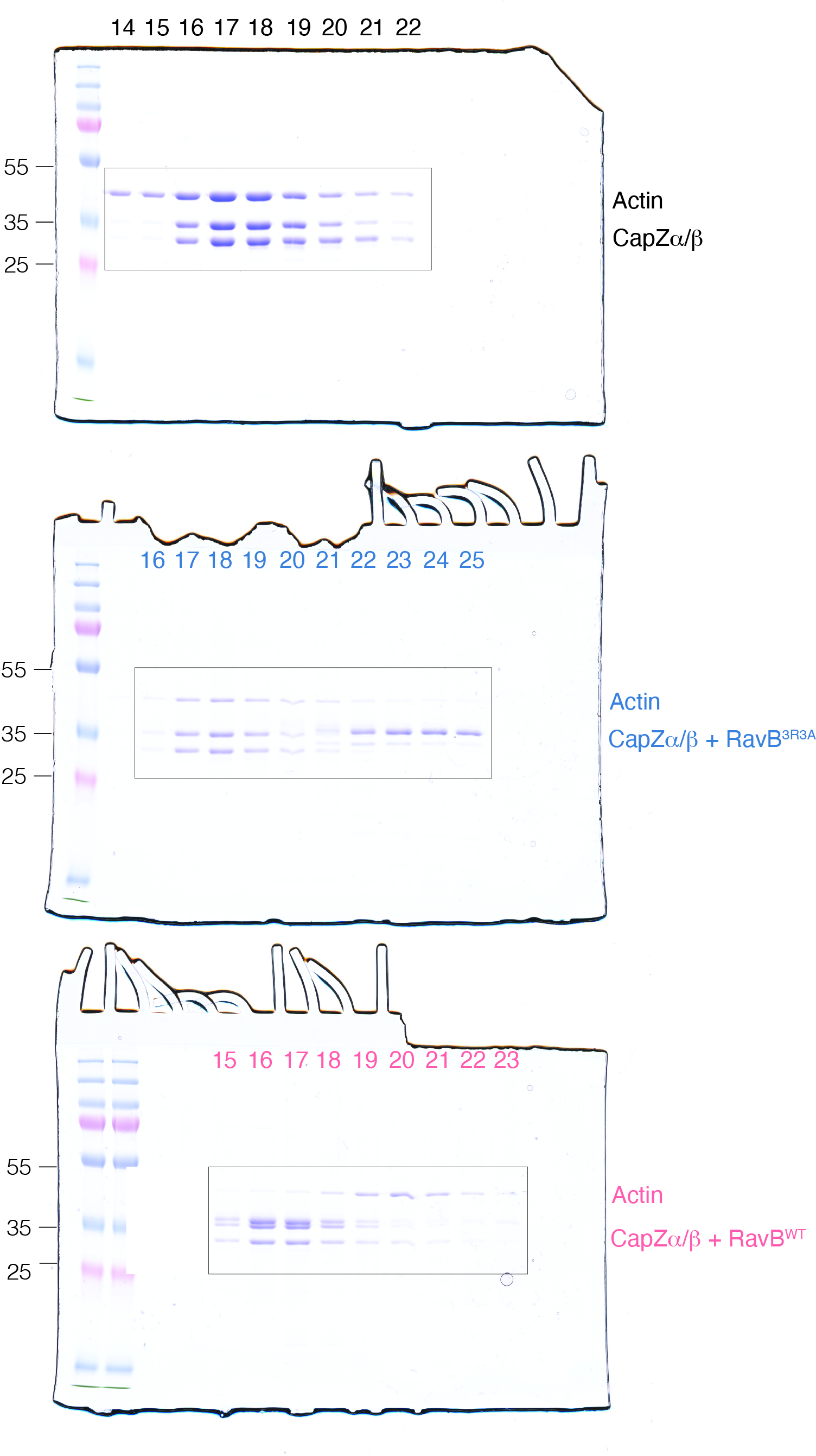

Supplement: Supplementary file 14 — Source data Fig. 5 [file 44318_2025_665_MOESM14_ESM.zip › Figure 5/5h.jpg]

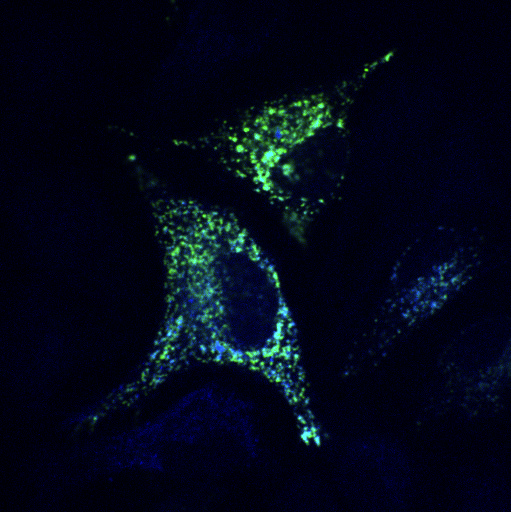

Supplement: Supplementary file 14 — Source data Fig. 5 [file 44318_2025_665_MOESM14_ESM.zip › Figure 5/5a/3RA EGFP RavB + mTagBFP Lamp1 + AF647 anti rabbit CapZB/100x EMCCD snap_1432.vsi]

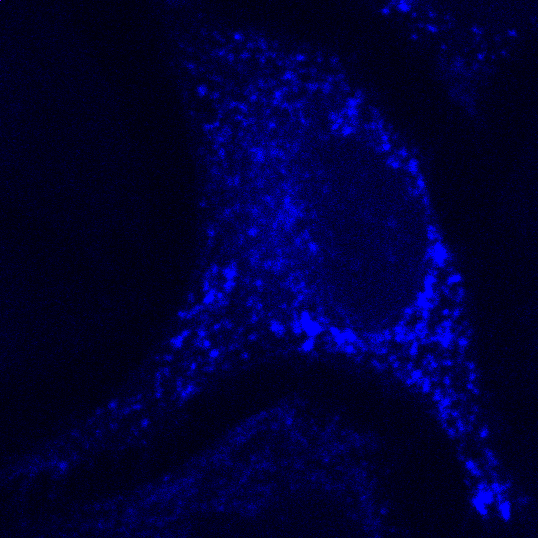

Supplement: Supplementary file 14 — Source data Fig. 5 [file 44318_2025_665_MOESM14_ESM.zip › Figure 5/5a/3RA EGFP RavB + mTagBFP Lamp1 + AF647 anti rabbit CapZB/1432 blue.tif]

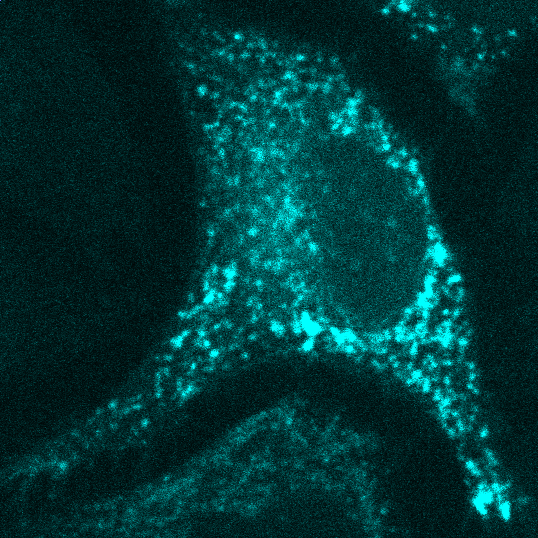

Supplement: Supplementary file 14 — Source data Fig. 5 [file 44318_2025_665_MOESM14_ESM.zip › Figure 5/5a/3RA EGFP RavB + mTagBFP Lamp1 + AF647 anti rabbit CapZB/1432 cyan.tif]

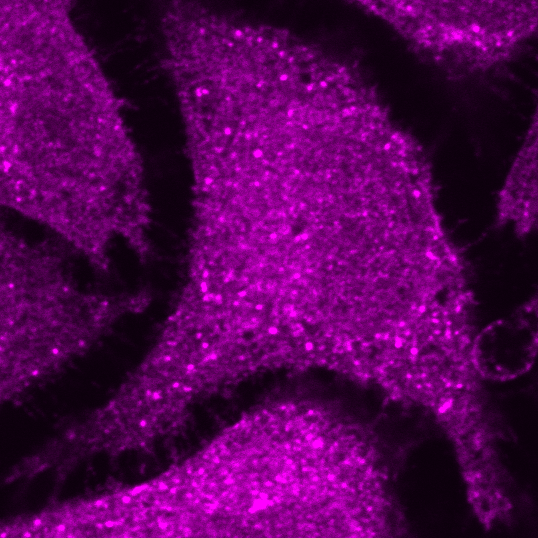

Supplement: Supplementary file 14 — Source data Fig. 5 [file 44318_2025_665_MOESM14_ESM.zip › Figure 5/5a/3RA EGFP RavB + mTagBFP Lamp1 + AF647 anti rabbit CapZB/1432 far red.tif]

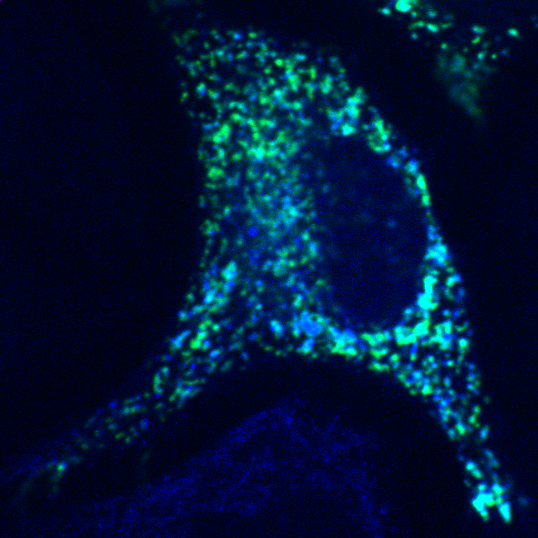

Supplement: Supplementary file 14 — Source data Fig. 5 [file 44318_2025_665_MOESM14_ESM.zip › Figure 5/5a/3RA EGFP RavB + mTagBFP Lamp1 + AF647 anti rabbit CapZB/1432 green blue.tif]

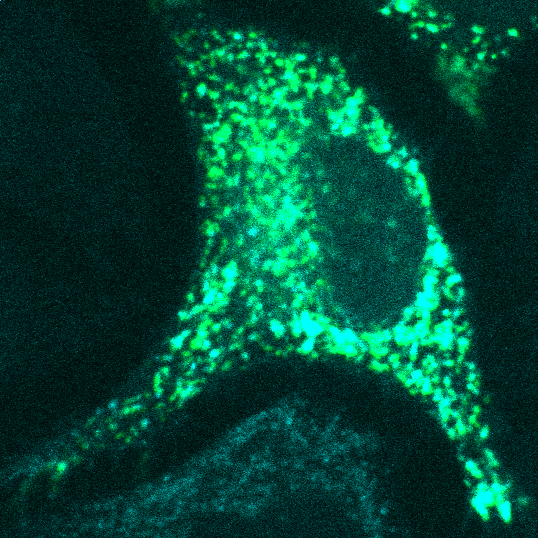

Supplement: Supplementary file 14 — Source data Fig. 5 [file 44318_2025_665_MOESM14_ESM.zip › Figure 5/5a/3RA EGFP RavB + mTagBFP Lamp1 + AF647 anti rabbit CapZB/1432 green cyan.tif]

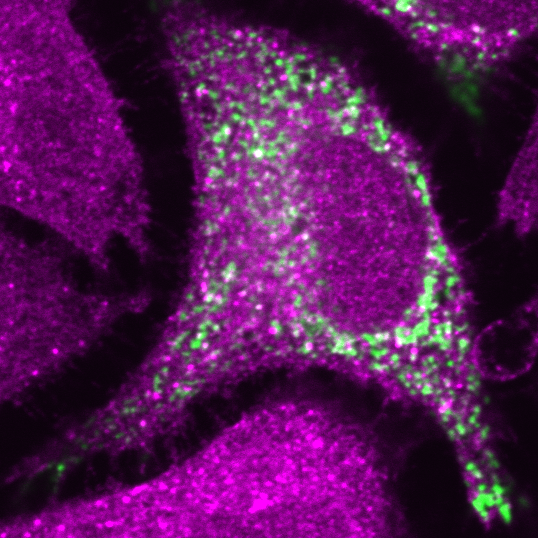

Supplement: Supplementary file 14 — Source data Fig. 5 [file 44318_2025_665_MOESM14_ESM.zip › Figure 5/5a/3RA EGFP RavB + mTagBFP Lamp1 + AF647 anti rabbit CapZB/1432 green far red.tif]

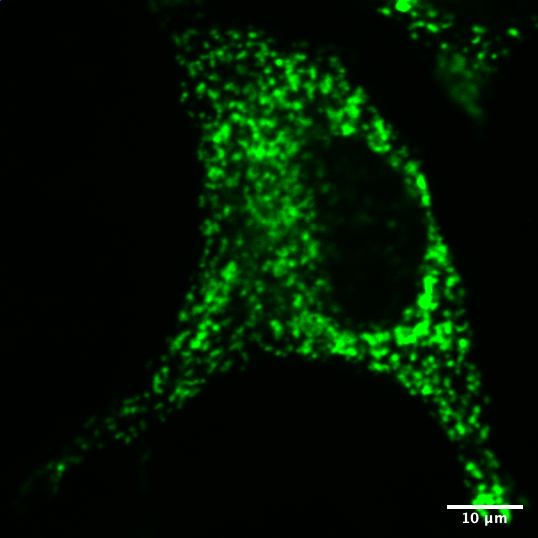

Supplement: Supplementary file 14 — Source data Fig. 5 [file 44318_2025_665_MOESM14_ESM.zip › Figure 5/5a/3RA EGFP RavB + mTagBFP Lamp1 + AF647 anti rabbit CapZB/1432 green scale.tif]

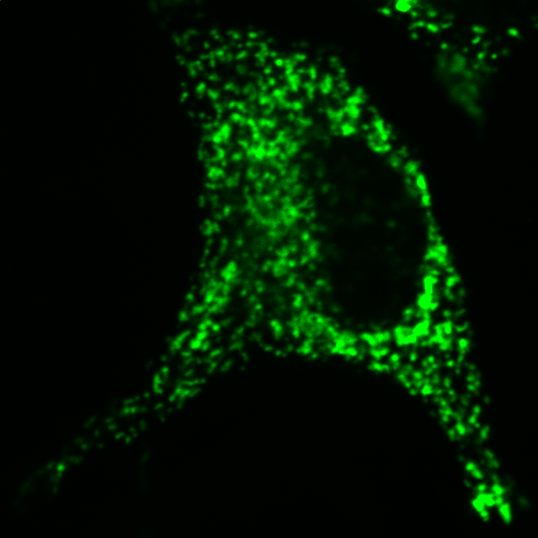

Supplement: Supplementary file 14 — Source data Fig. 5 [file 44318_2025_665_MOESM14_ESM.zip › Figure 5/5a/3RA EGFP RavB + mTagBFP Lamp1 + AF647 anti rabbit CapZB/1432 green.tif]

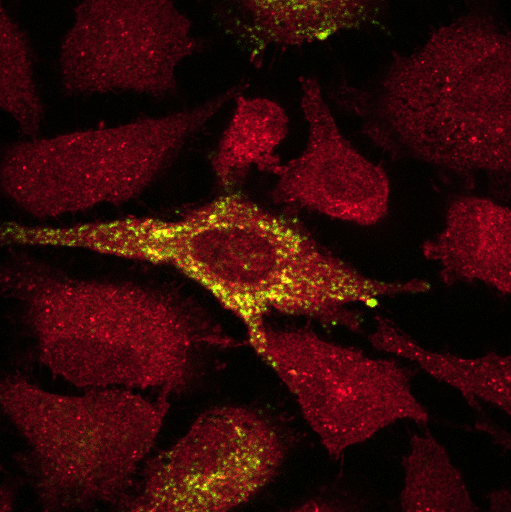

Supplement: Supplementary file 14 — Source data Fig. 5 [file 44318_2025_665_MOESM14_ESM.zip › Figure 5/5a/CTD EGFP RavB + mTagBFP Lamp1 + AF647 anti rabbit CapZB/100x EMCCD snap_1547.vsi]

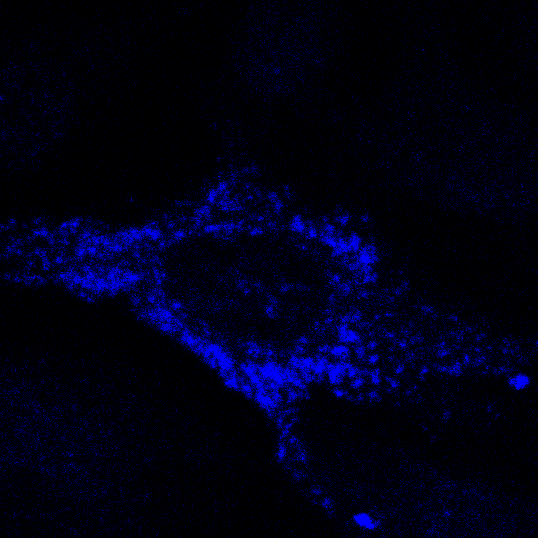

Supplement: Supplementary file 14 — Source data Fig. 5 [file 44318_2025_665_MOESM14_ESM.zip › Figure 5/5a/CTD EGFP RavB + mTagBFP Lamp1 + AF647 anti rabbit CapZB/1547 blue.tif]

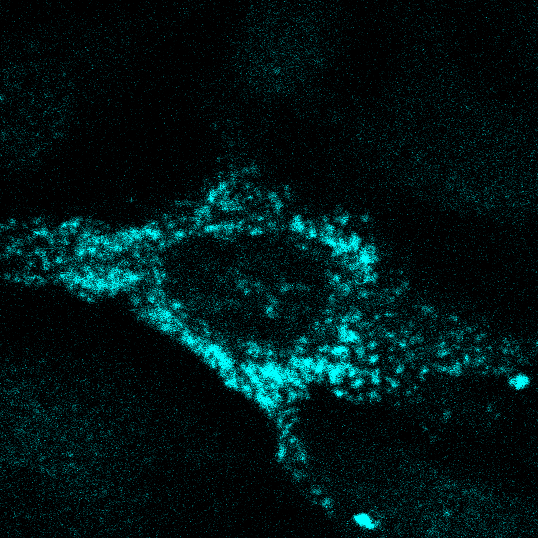

Supplement: Supplementary file 14 — Source data Fig. 5 [file 44318_2025_665_MOESM14_ESM.zip › Figure 5/5a/CTD EGFP RavB + mTagBFP Lamp1 + AF647 anti rabbit CapZB/1547 cyan.tif]

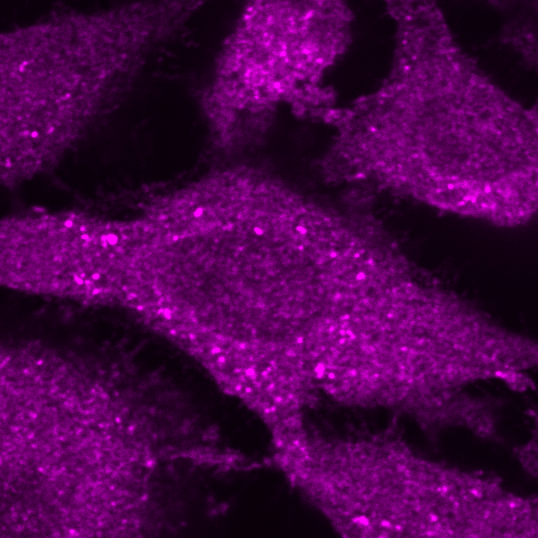

Supplement: Supplementary file 14 — Source data Fig. 5 [file 44318_2025_665_MOESM14_ESM.zip › Figure 5/5a/CTD EGFP RavB + mTagBFP Lamp1 + AF647 anti rabbit CapZB/1547 far red.tif]

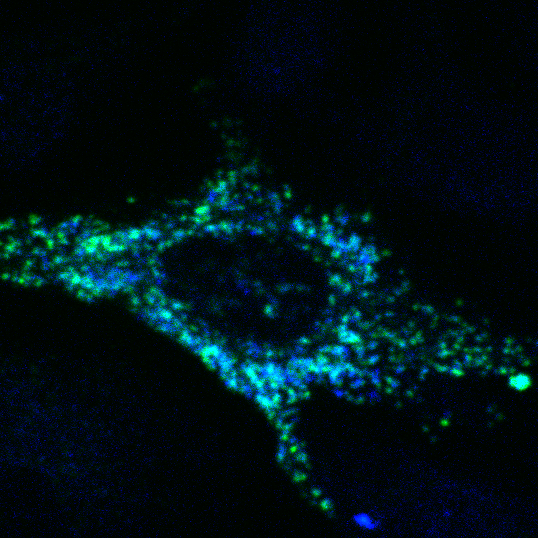

Supplement: Supplementary file 14 — Source data Fig. 5 [file 44318_2025_665_MOESM14_ESM.zip › Figure 5/5a/CTD EGFP RavB + mTagBFP Lamp1 + AF647 anti rabbit CapZB/1547 green blue.tif]

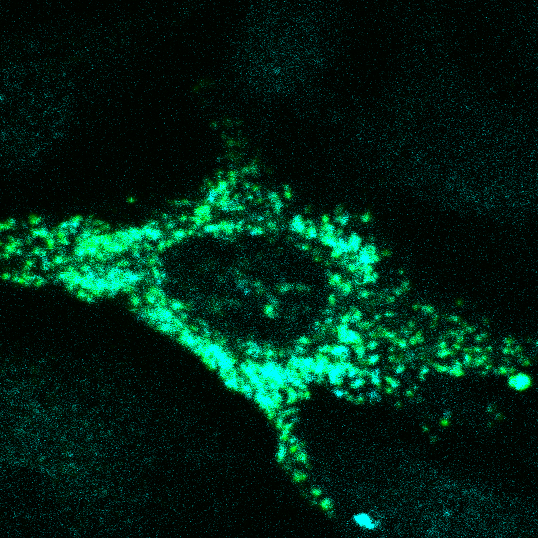

Supplement: Supplementary file 14 — Source data Fig. 5 [file 44318_2025_665_MOESM14_ESM.zip › Figure 5/5a/CTD EGFP RavB + mTagBFP Lamp1 + AF647 anti rabbit CapZB/1547 green cyan.tif]

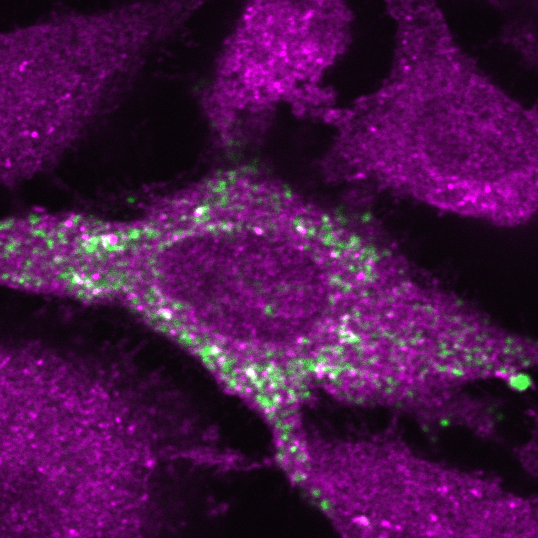

Supplement: Supplementary file 14 — Source data Fig. 5 [file 44318_2025_665_MOESM14_ESM.zip › Figure 5/5a/CTD EGFP RavB + mTagBFP Lamp1 + AF647 anti rabbit CapZB/1547 green far red.tif]

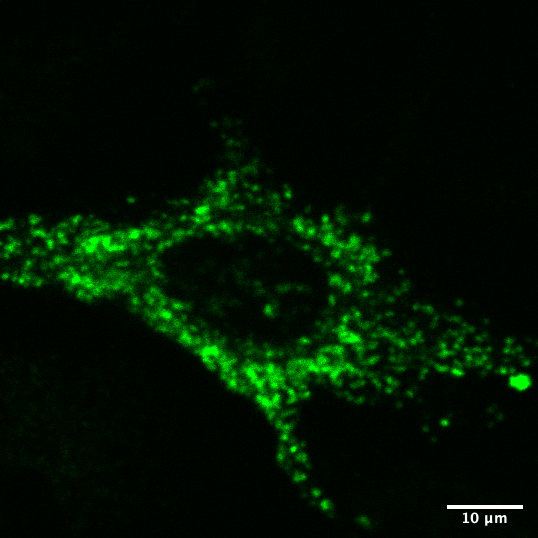

Supplement: Supplementary file 14 — Source data Fig. 5 [file 44318_2025_665_MOESM14_ESM.zip › Figure 5/5a/CTD EGFP RavB + mTagBFP Lamp1 + AF647 anti rabbit CapZB/1547 green scale.tif]

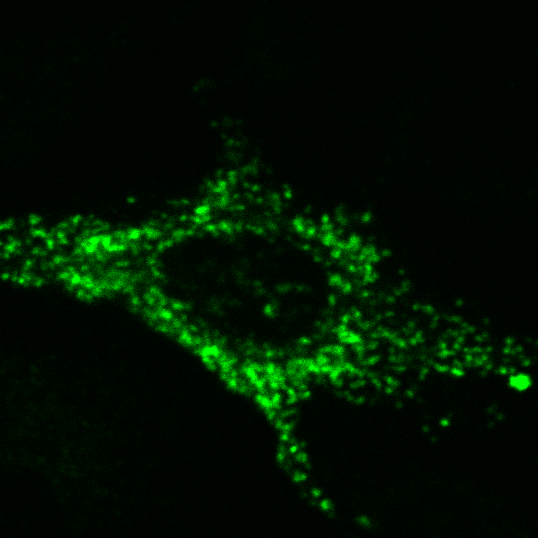

Supplement: Supplementary file 14 — Source data Fig. 5 [file 44318_2025_665_MOESM14_ESM.zip › Figure 5/5a/CTD EGFP RavB + mTagBFP Lamp1 + AF647 anti rabbit CapZB/1547 green.tif]

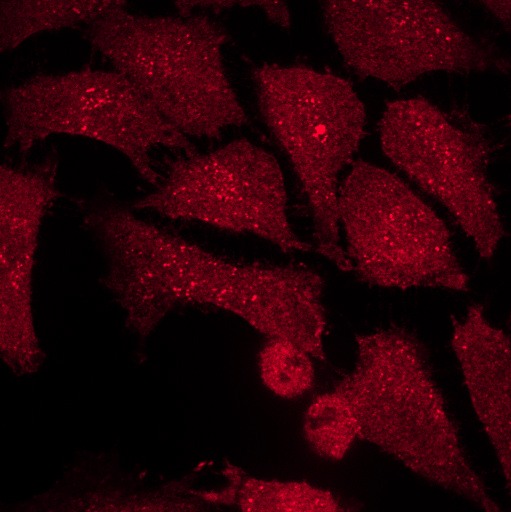

Supplement: Supplementary file 14 — Source data Fig. 5 [file 44318_2025_665_MOESM14_ESM.zip › Figure 5/5a/H206A EGFP RavB + mTagBFP Lamp1 + AF647 anti rabbit CapZB/100x EMCCD snap_1622.vsi]

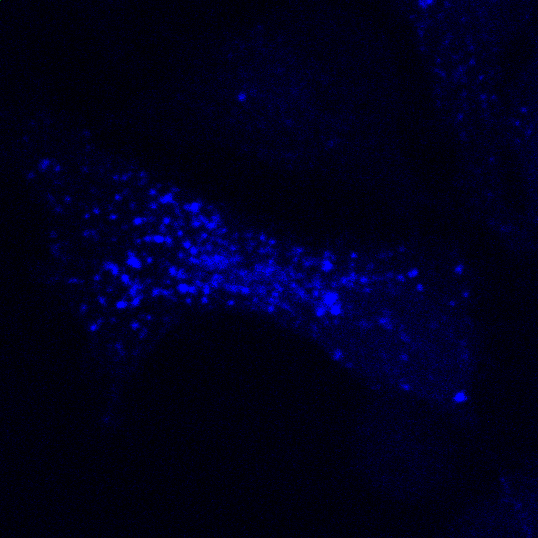

Supplement: Supplementary file 14 — Source data Fig. 5 [file 44318_2025_665_MOESM14_ESM.zip › Figure 5/5a/H206A EGFP RavB + mTagBFP Lamp1 + AF647 anti rabbit CapZB/1622 blue.tif]

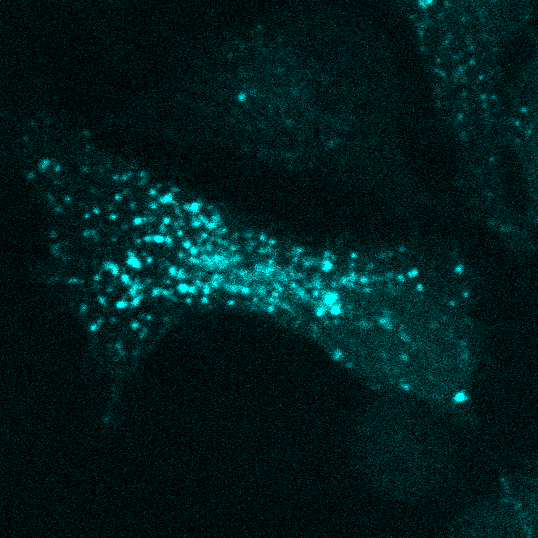

Supplement: Supplementary file 14 — Source data Fig. 5 [file 44318_2025_665_MOESM14_ESM.zip › Figure 5/5a/H206A EGFP RavB + mTagBFP Lamp1 + AF647 anti rabbit CapZB/1622 cyan.tif]

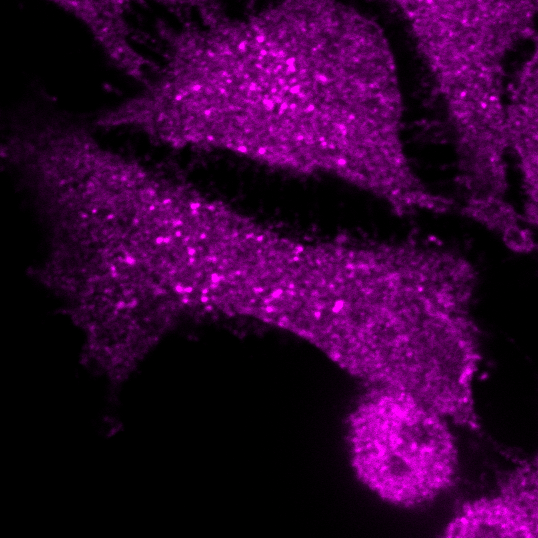

Supplement: Supplementary file 14 — Source data Fig. 5 [file 44318_2025_665_MOESM14_ESM.zip › Figure 5/5a/H206A EGFP RavB + mTagBFP Lamp1 + AF647 anti rabbit CapZB/1622 far red.tif]

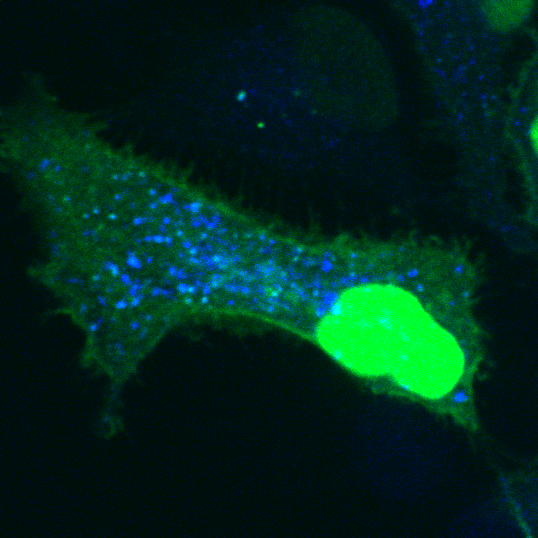

Supplement: Supplementary file 14 — Source data Fig. 5 [file 44318_2025_665_MOESM14_ESM.zip › Figure 5/5a/H206A EGFP RavB + mTagBFP Lamp1 + AF647 anti rabbit CapZB/1622 green blue.tif]

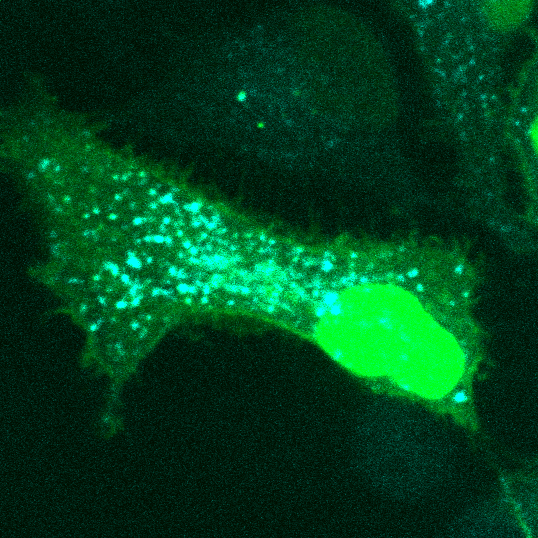

Supplement: Supplementary file 14 — Source data Fig. 5 [file 44318_2025_665_MOESM14_ESM.zip › Figure 5/5a/H206A EGFP RavB + mTagBFP Lamp1 + AF647 anti rabbit CapZB/1622 green cyan.tif]

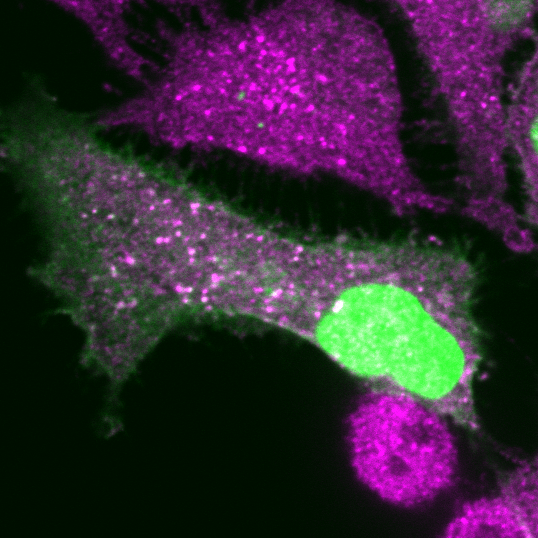

Supplement: Supplementary file 14 — Source data Fig. 5 [file 44318_2025_665_MOESM14_ESM.zip › Figure 5/5a/H206A EGFP RavB + mTagBFP Lamp1 + AF647 anti rabbit CapZB/1622 green far red.tif]

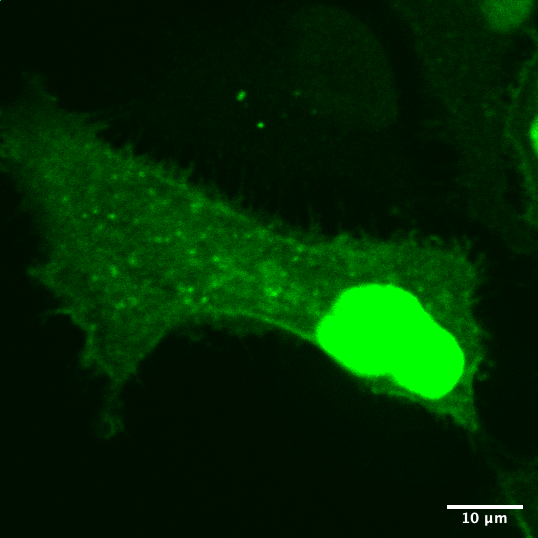

Supplement: Supplementary file 14 — Source data Fig. 5 [file 44318_2025_665_MOESM14_ESM.zip › Figure 5/5a/H206A EGFP RavB + mTagBFP Lamp1 + AF647 anti rabbit CapZB/1622 green scale.tif]

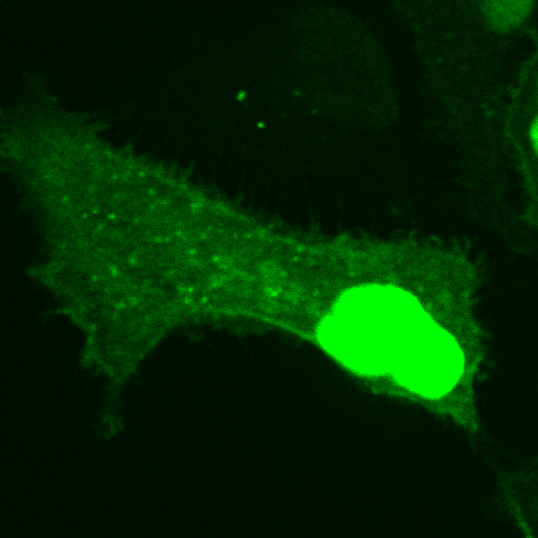

Supplement: Supplementary file 14 — Source data Fig. 5 [file 44318_2025_665_MOESM14_ESM.zip › Figure 5/5a/H206A EGFP RavB + mTagBFP Lamp1 + AF647 anti rabbit CapZB/1622 green.tif]

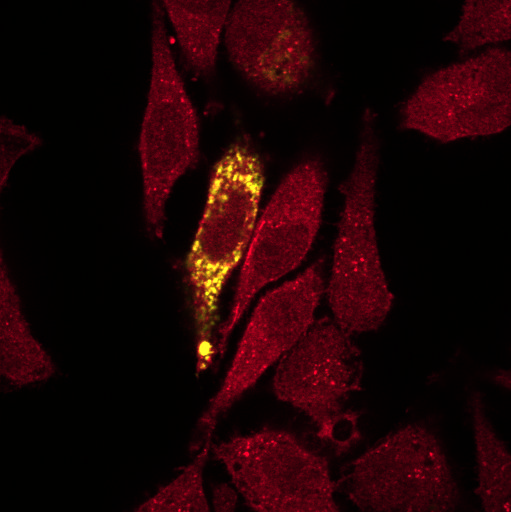

Supplement: Supplementary file 14 — Source data Fig. 5 [file 44318_2025_665_MOESM14_ESM.zip › Figure 5/5a/WT EGFP RavB + mTagBFP Lamp1 + AF647 anti rabbit CapZB/100x EMCCD snap_1528.vsi]

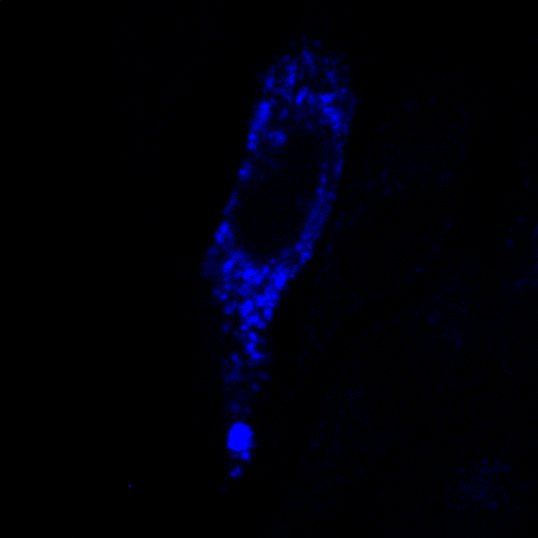

Supplement: Supplementary file 14 — Source data Fig. 5 [file 44318_2025_665_MOESM14_ESM.zip › Figure 5/5a/WT EGFP RavB + mTagBFP Lamp1 + AF647 anti rabbit CapZB/1528 blue.tif]

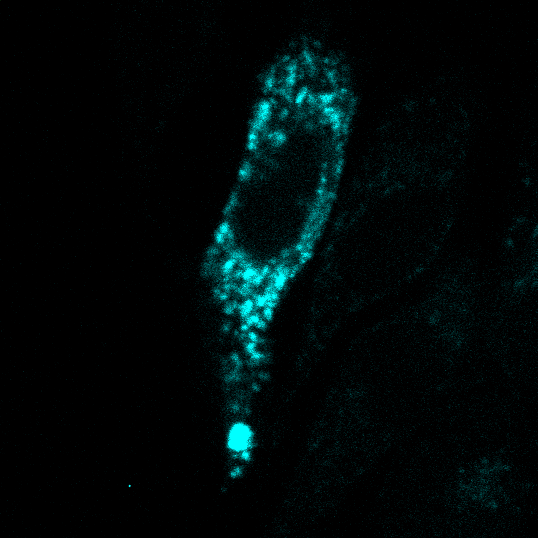

Supplement: Supplementary file 14 — Source data Fig. 5 [file 44318_2025_665_MOESM14_ESM.zip › Figure 5/5a/WT EGFP RavB + mTagBFP Lamp1 + AF647 anti rabbit CapZB/1528 cyan.tif]

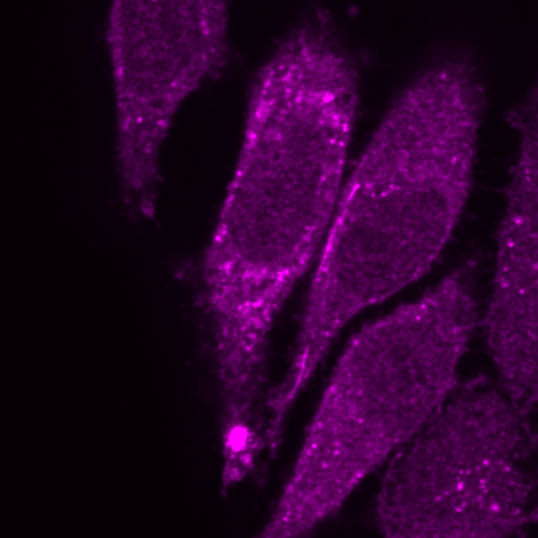

Supplement: Supplementary file 14 — Source data Fig. 5 [file 44318_2025_665_MOESM14_ESM.zip › Figure 5/5a/WT EGFP RavB + mTagBFP Lamp1 + AF647 anti rabbit CapZB/1528 far red.tif]

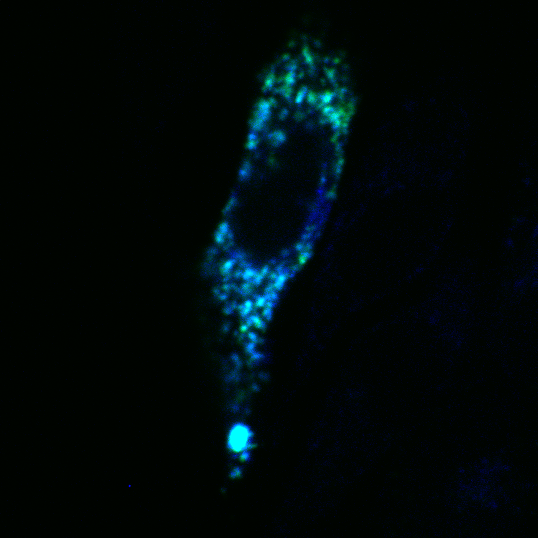

Supplement: Supplementary file 14 — Source data Fig. 5 [file 44318_2025_665_MOESM14_ESM.zip › Figure 5/5a/WT EGFP RavB + mTagBFP Lamp1 + AF647 anti rabbit CapZB/1528 green blue.tif]

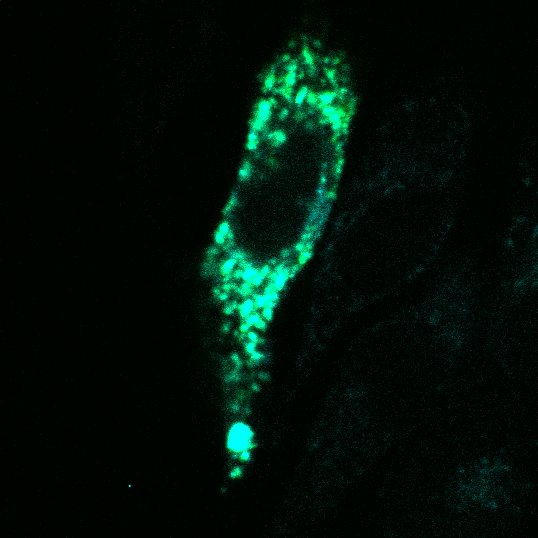

Supplement: Supplementary file 14 — Source data Fig. 5 [file 44318_2025_665_MOESM14_ESM.zip › Figure 5/5a/WT EGFP RavB + mTagBFP Lamp1 + AF647 anti rabbit CapZB/1528 green cyan.tif]

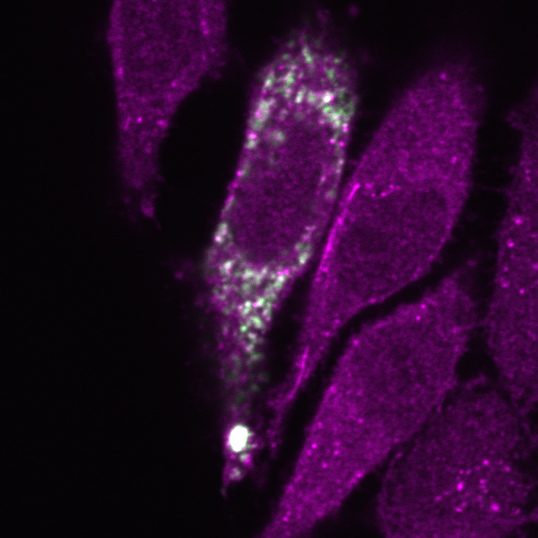

Supplement: Supplementary file 14 — Source data Fig. 5 [file 44318_2025_665_MOESM14_ESM.zip › Figure 5/5a/WT EGFP RavB + mTagBFP Lamp1 + AF647 anti rabbit CapZB/1528 green far red.tif]

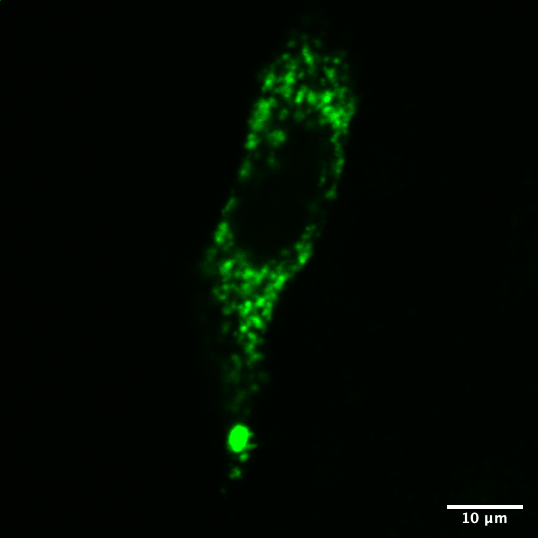

Supplement: Supplementary file 14 — Source data Fig. 5 [file 44318_2025_665_MOESM14_ESM.zip › Figure 5/5a/WT EGFP RavB + mTagBFP Lamp1 + AF647 anti rabbit CapZB/1528 green scale.tif]

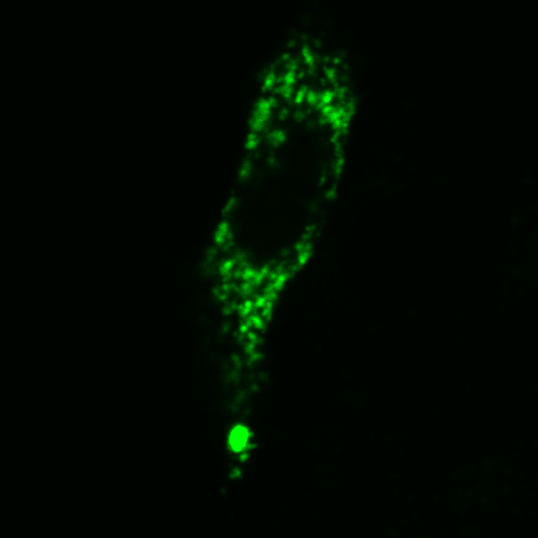

Supplement: Supplementary file 14 — Source data Fig. 5 [file 44318_2025_665_MOESM14_ESM.zip › Figure 5/5a/WT EGFP RavB + mTagBFP Lamp1 + AF647 anti rabbit CapZB/1528 green.tif]

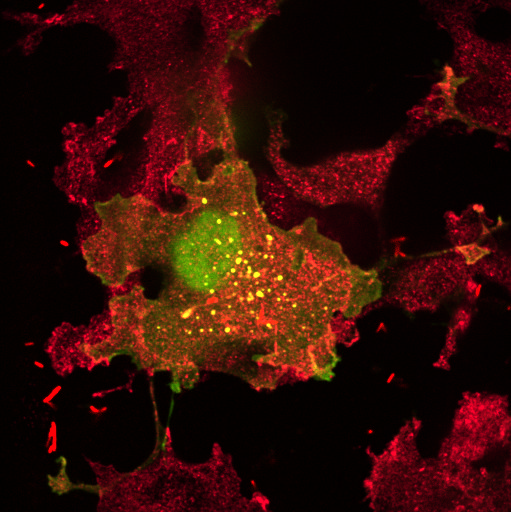

Supplement: Supplementary file 14 — Source data Fig. 5 [file 44318_2025_665_MOESM14_ESM.zip › Figure 5/5d/mch-lp03 + EGFP-H206A RavB + AF647 anti rabbit CapZB/100x EMCCD Z StpSz 0.25_1426.vsi]

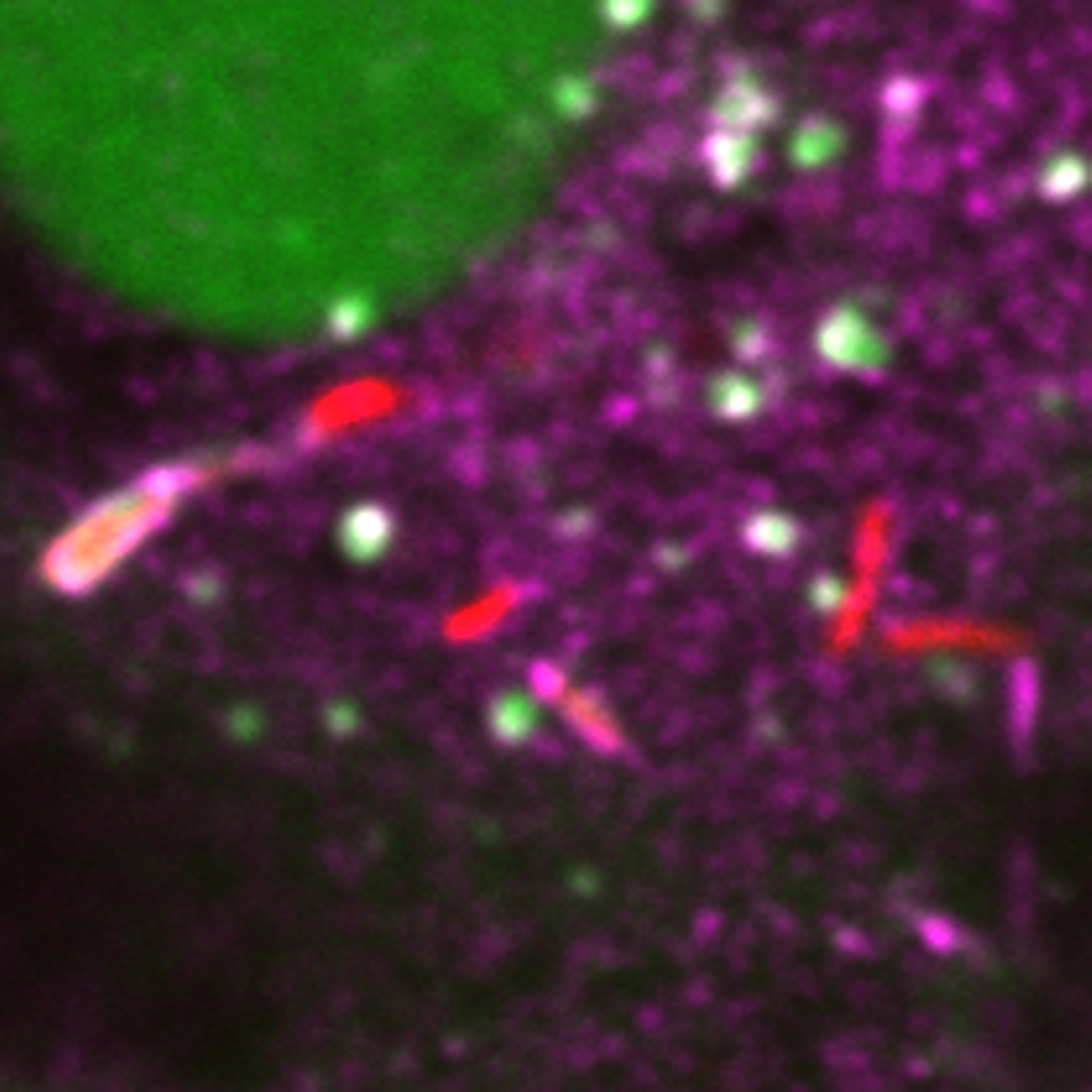

Supplement: Supplementary file 14 — Source data Fig. 5 [file 44318_2025_665_MOESM14_ESM.zip › Figure 5/5d/mch-lp03 + EGFP-H206A RavB + AF647 anti rabbit CapZB/inset scaled all.tif]

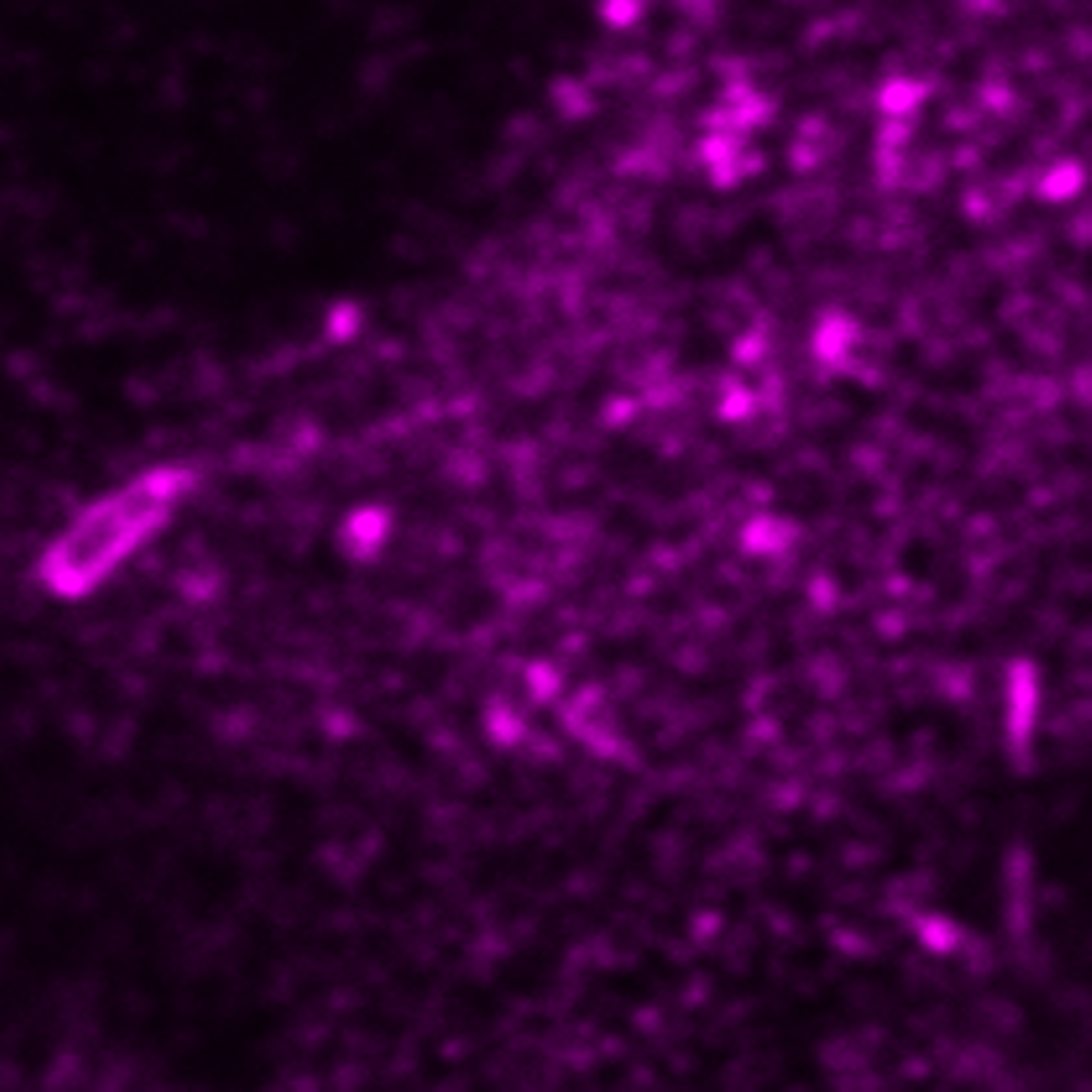

Supplement: Supplementary file 14 — Source data Fig. 5 [file 44318_2025_665_MOESM14_ESM.zip › Figure 5/5d/mch-lp03 + EGFP-H206A RavB + AF647 anti rabbit CapZB/inset scaled far red.tif]

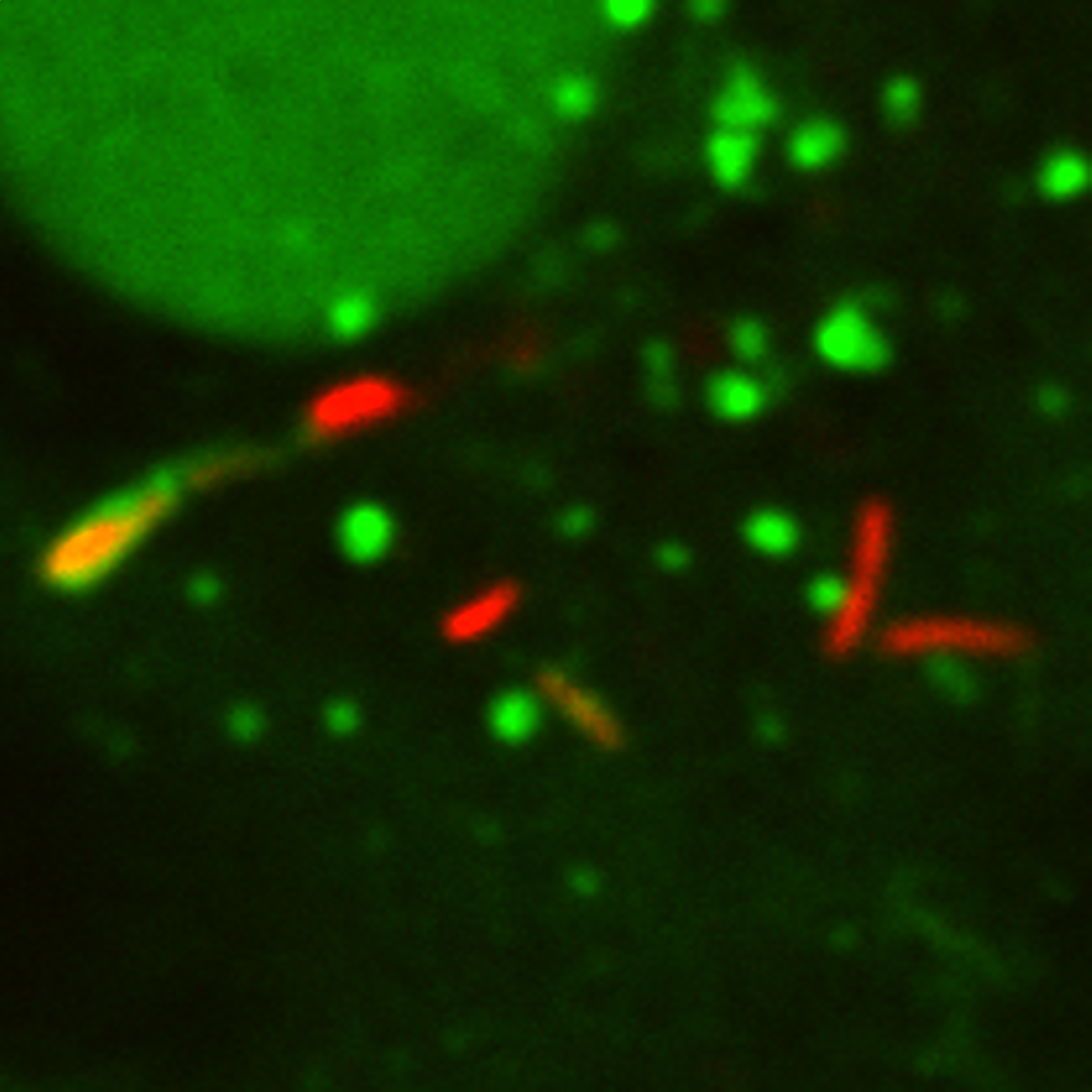

Supplement: Supplementary file 14 — Source data Fig. 5 [file 44318_2025_665_MOESM14_ESM.zip › Figure 5/5d/mch-lp03 + EGFP-H206A RavB + AF647 anti rabbit CapZB/inset scaled green red.tif]

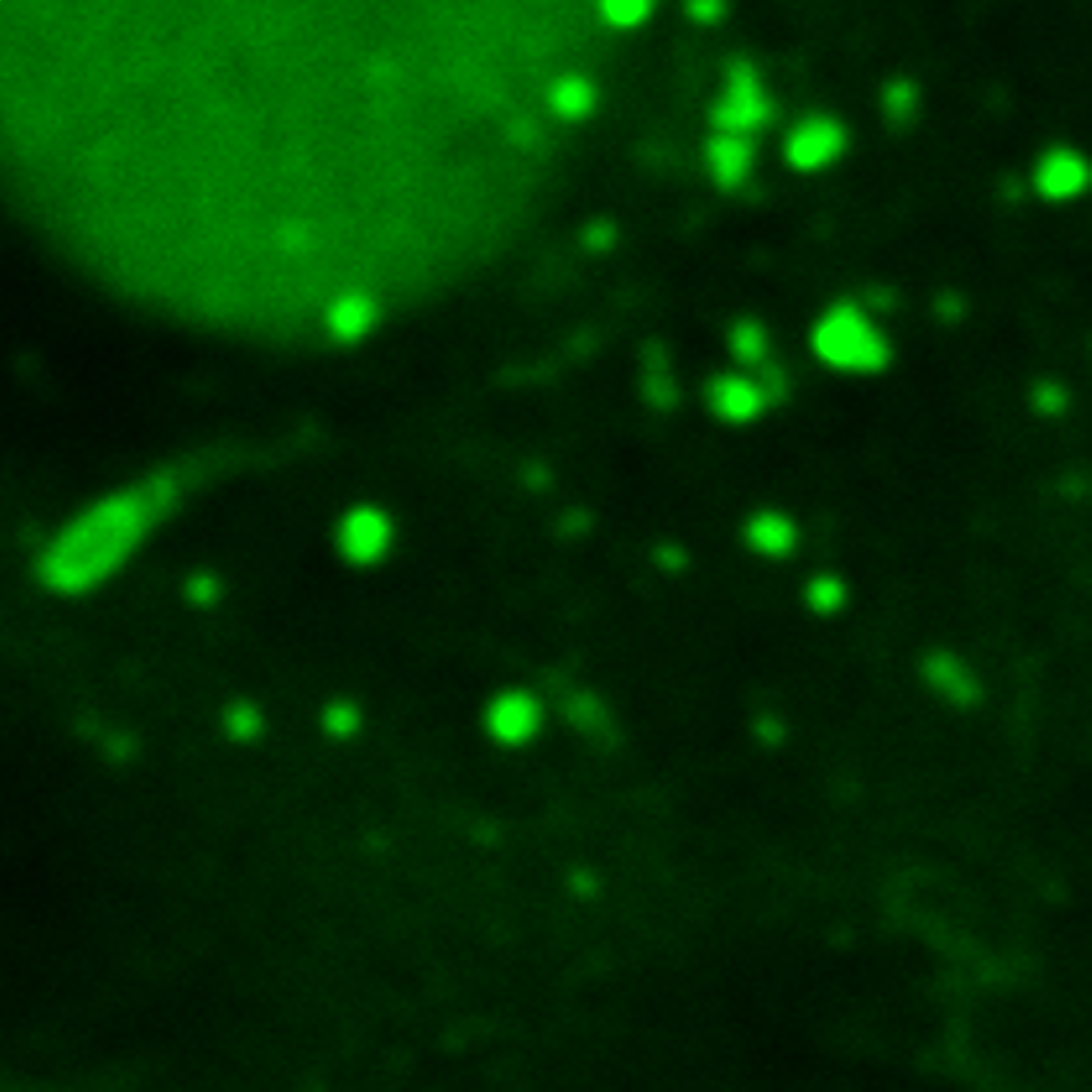

Supplement: Supplementary file 14 — Source data Fig. 5 [file 44318_2025_665_MOESM14_ESM.zip › Figure 5/5d/mch-lp03 + EGFP-H206A RavB + AF647 anti rabbit CapZB/inset scaled green.tif]

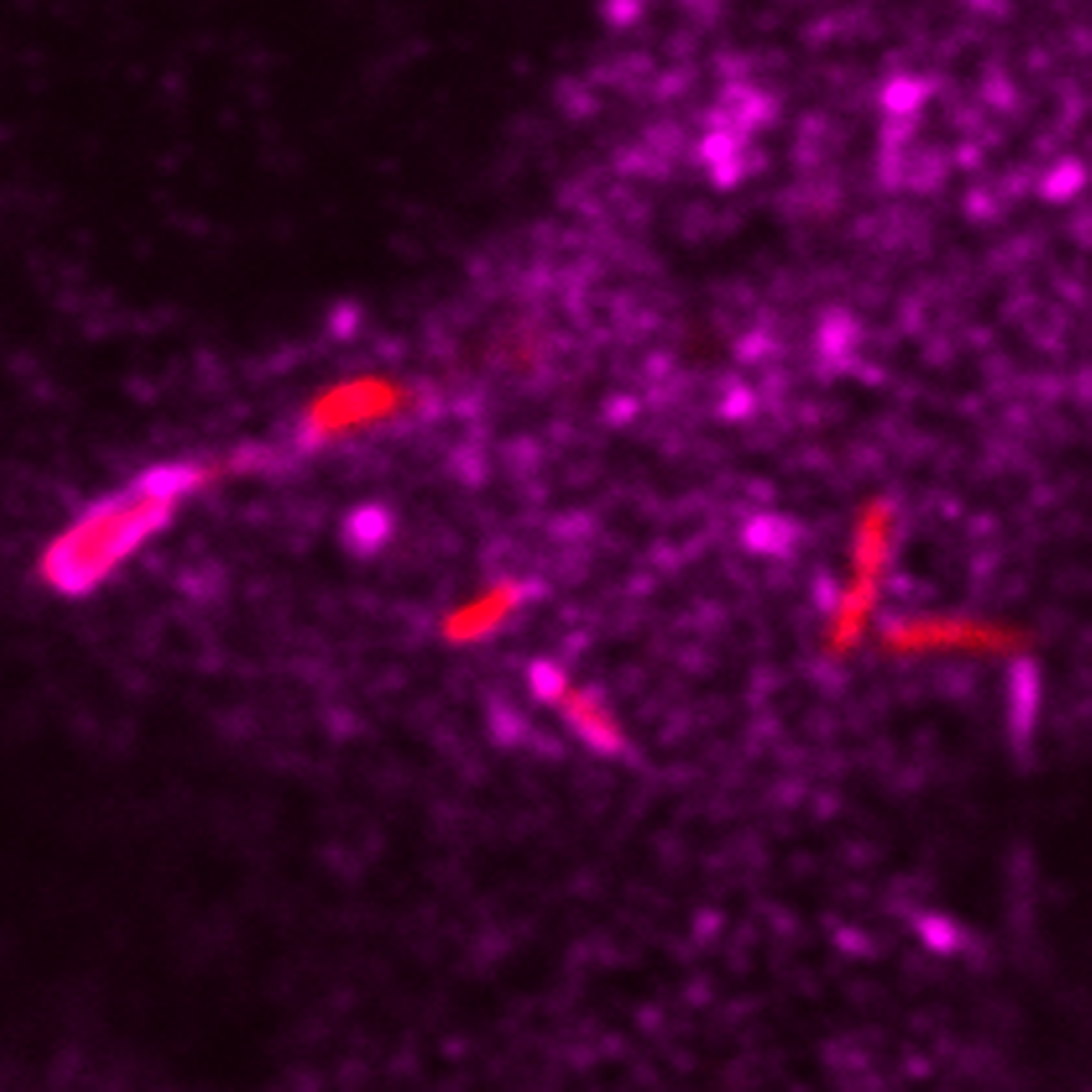

Supplement: Supplementary file 14 — Source data Fig. 5 [file 44318_2025_665_MOESM14_ESM.zip › Figure 5/5d/mch-lp03 + EGFP-H206A RavB + AF647 anti rabbit CapZB/inset scaled red far red.tif]

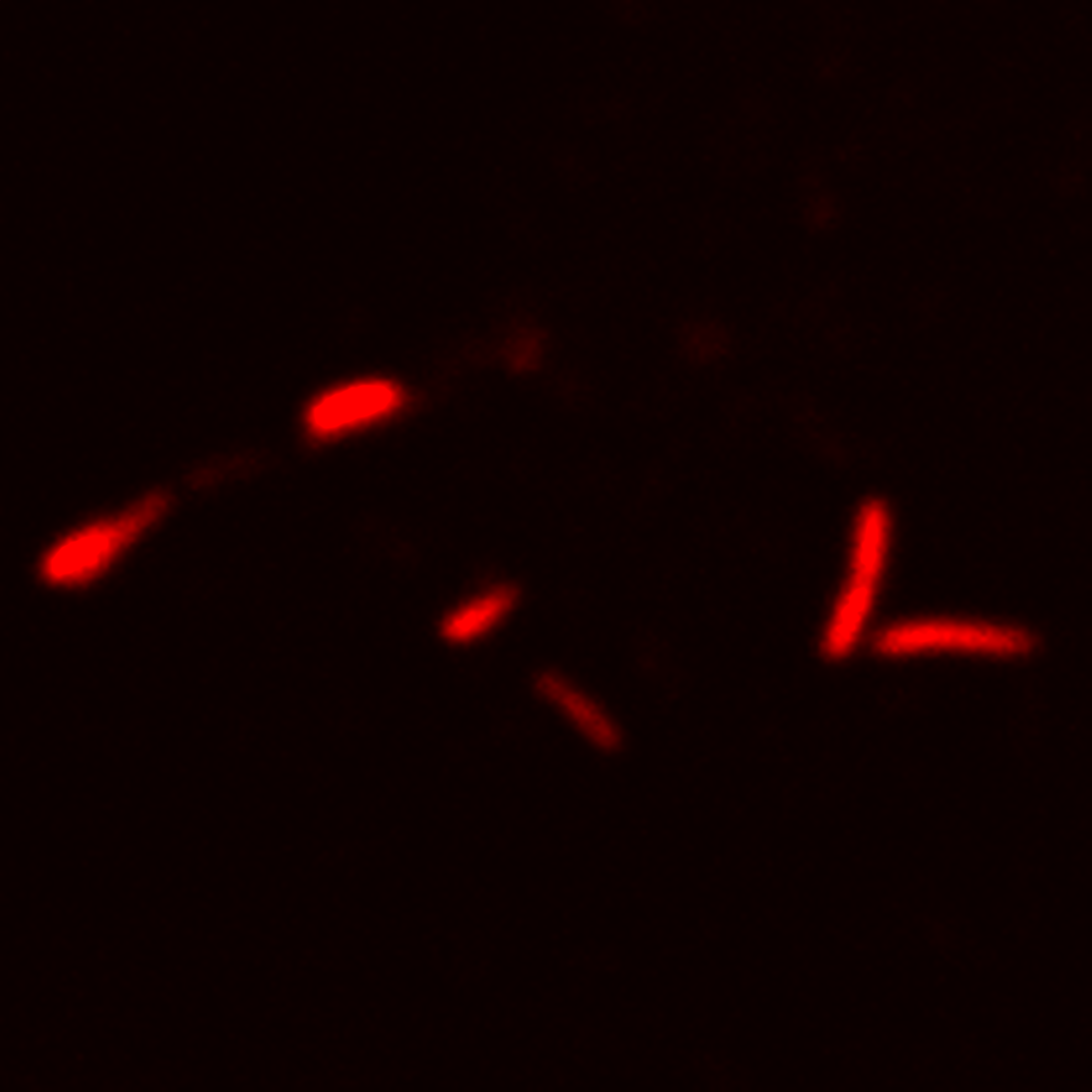

Supplement: Supplementary file 14 — Source data Fig. 5 [file 44318_2025_665_MOESM14_ESM.zip › Figure 5/5d/mch-lp03 + EGFP-H206A RavB + AF647 anti rabbit CapZB/inset scaled red.tif]

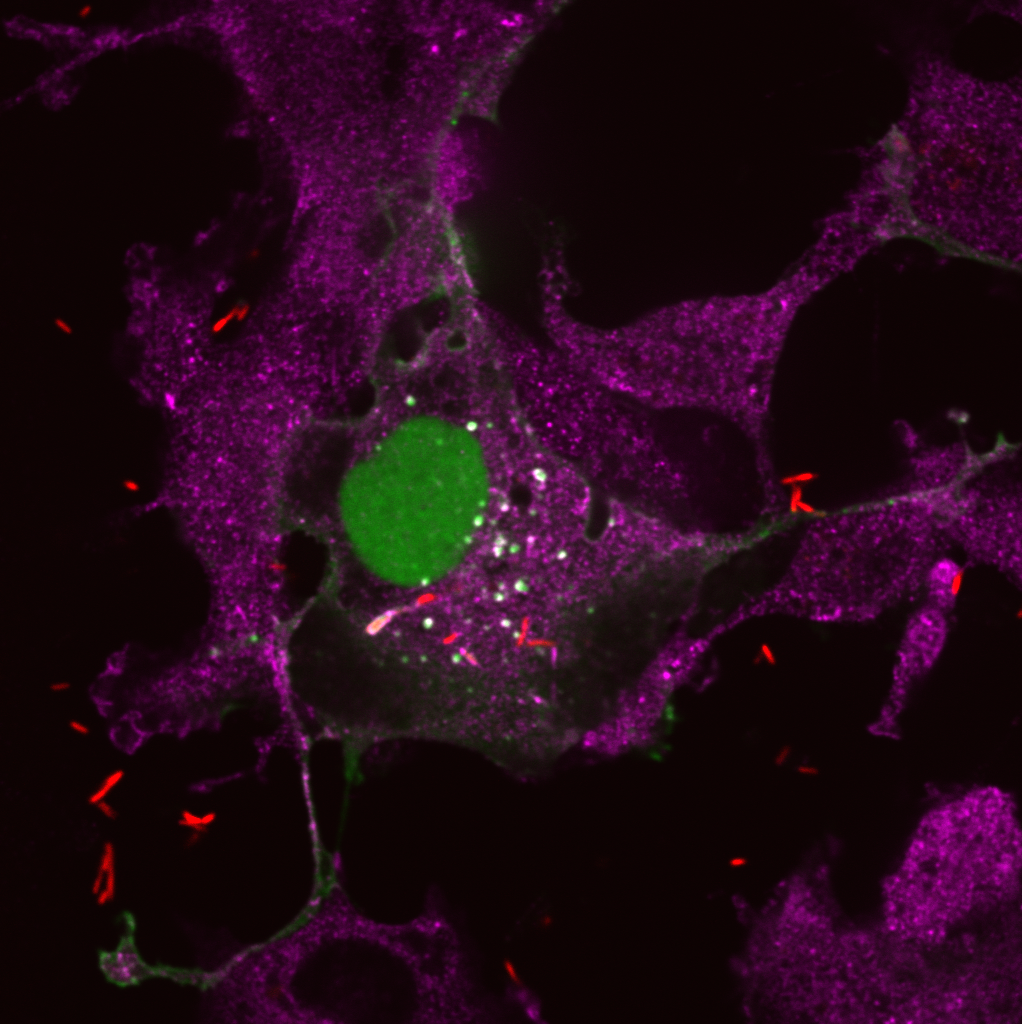

Supplement: Supplementary file 14 — Source data Fig. 5 [file 44318_2025_665_MOESM14_ESM.zip › Figure 5/5d/mch-lp03 + EGFP-H206A RavB + AF647 anti rabbit CapZB/original all.tif]

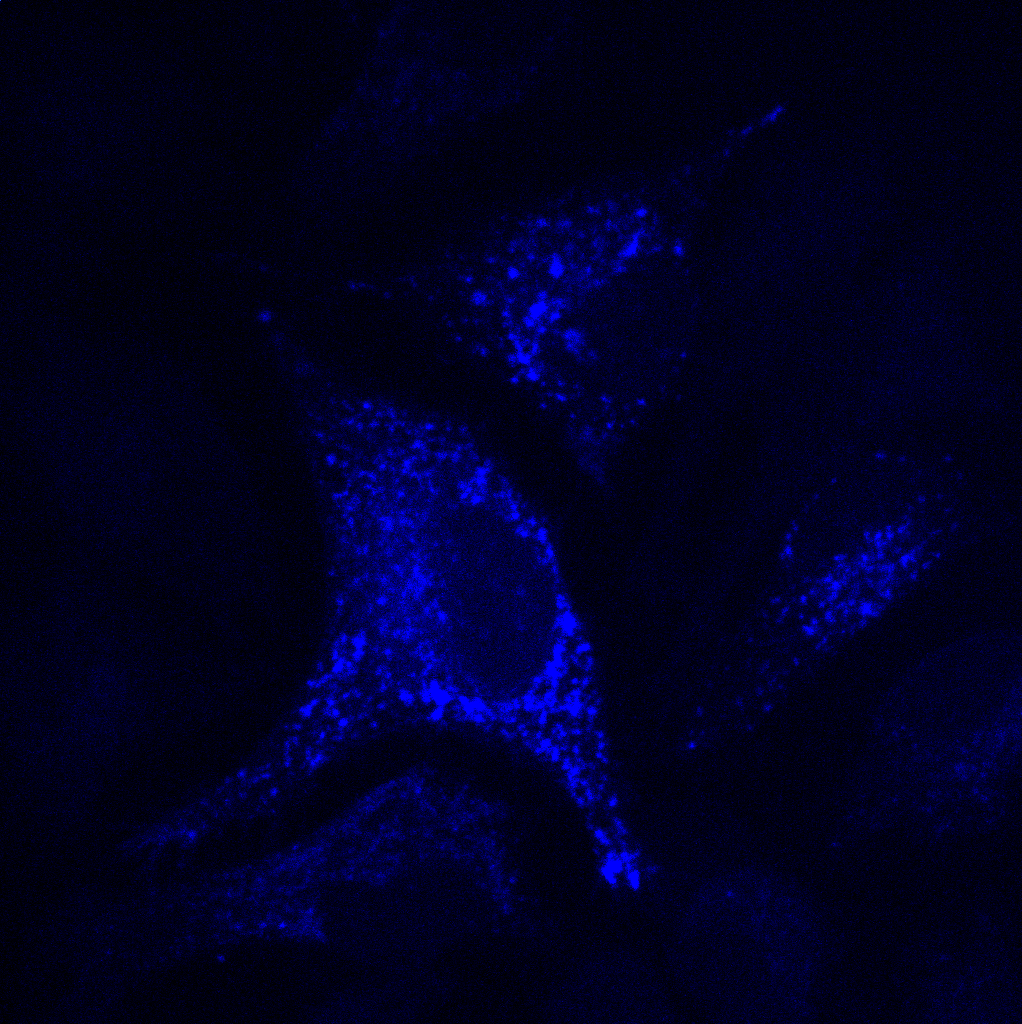

Supplement: Supplementary file 14 — Source data Fig. 5 [file 44318_2025_665_MOESM14_ESM.zip › Figure 5/5a/3RA EGFP RavB + mTagBFP Lamp1 + AF647 anti rabbit CapZB/original/1432 blue.tif]

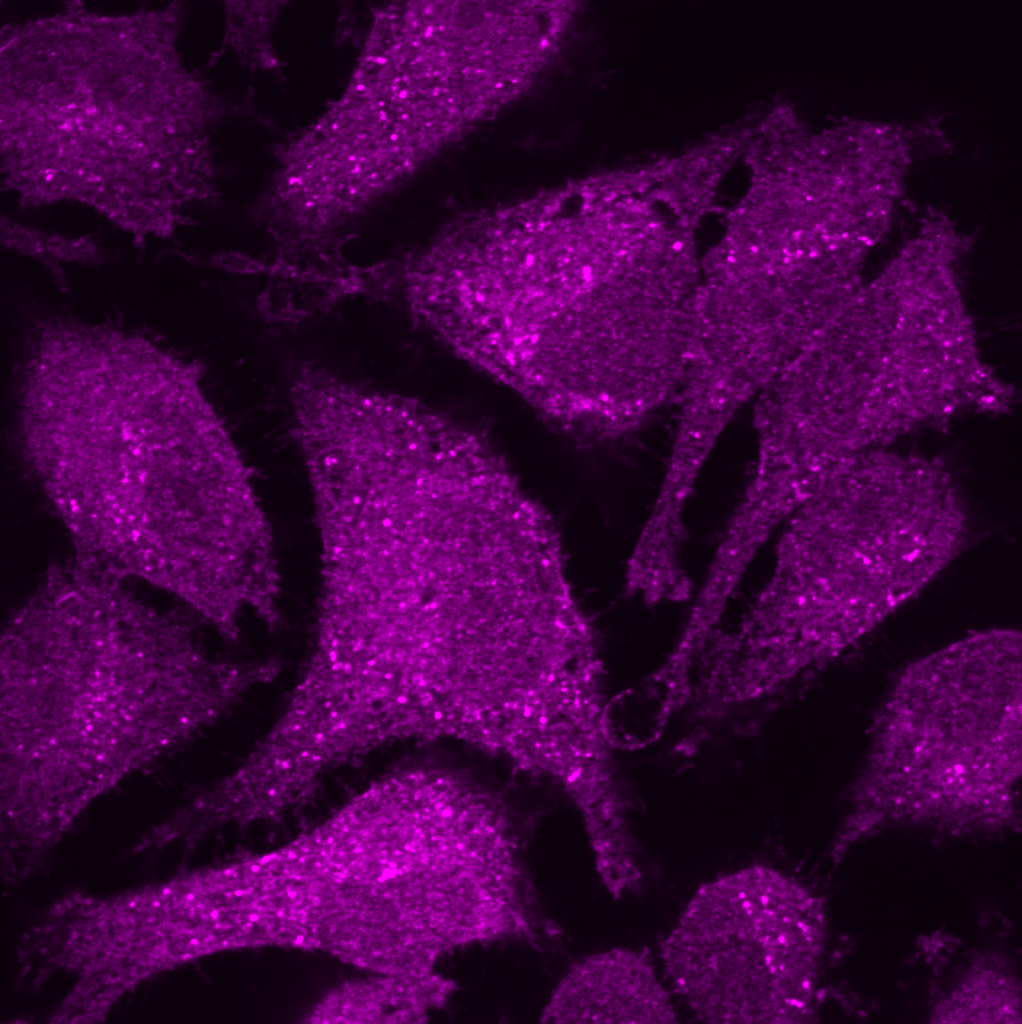

Supplement: Supplementary file 14 — Source data Fig. 5 [file 44318_2025_665_MOESM14_ESM.zip › Figure 5/5a/3RA EGFP RavB + mTagBFP Lamp1 + AF647 anti rabbit CapZB/original/1432 far red.tif]

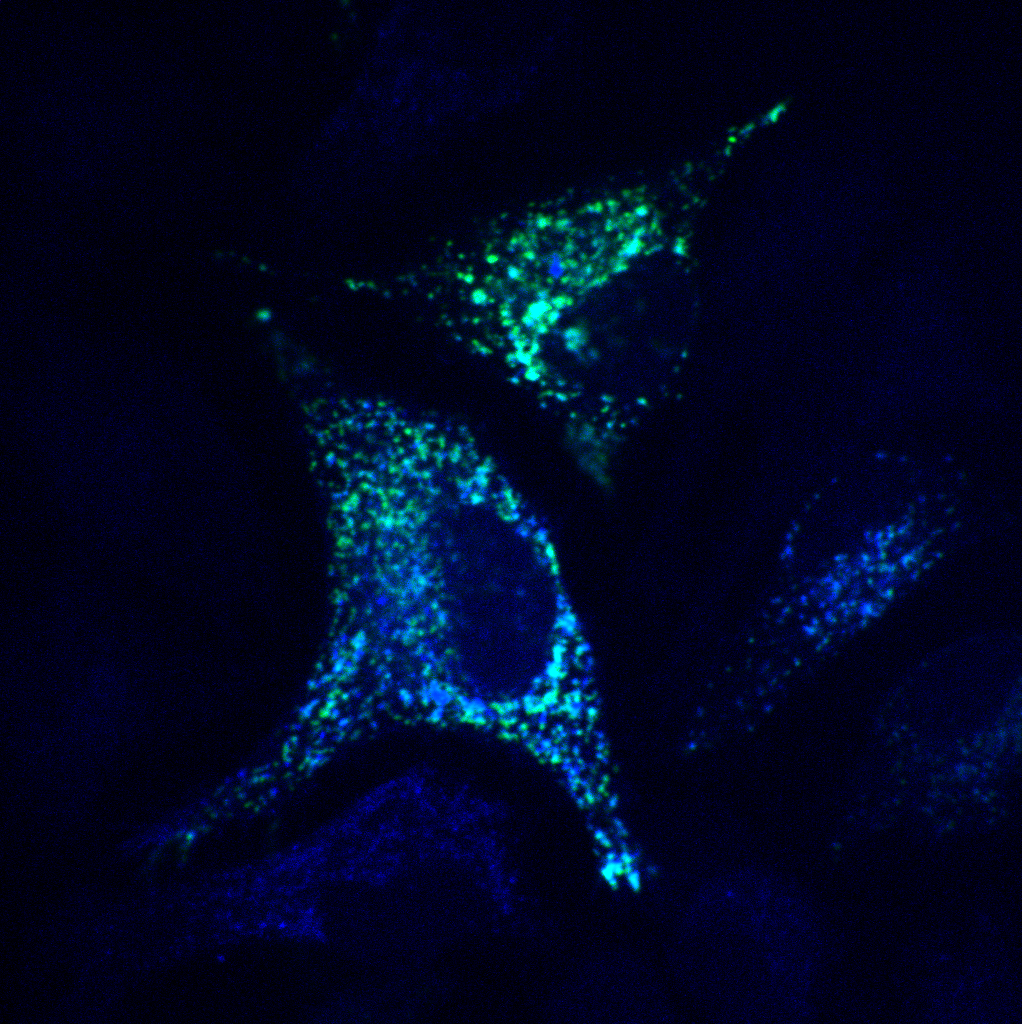

Supplement: Supplementary file 14 — Source data Fig. 5 [file 44318_2025_665_MOESM14_ESM.zip › Figure 5/5a/3RA EGFP RavB + mTagBFP Lamp1 + AF647 anti rabbit CapZB/original/1432 green blue.tif]

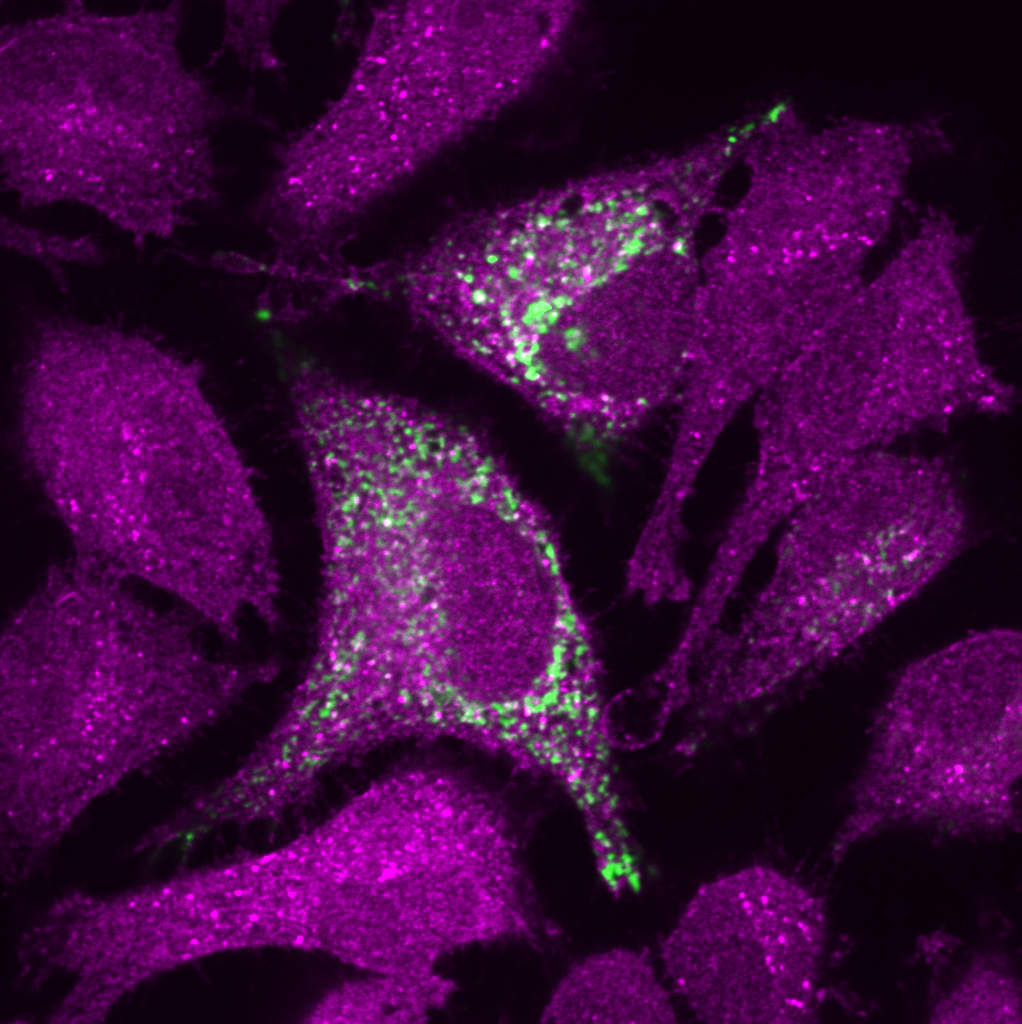

Supplement: Supplementary file 14 — Source data Fig. 5 [file 44318_2025_665_MOESM14_ESM.zip › Figure 5/5a/3RA EGFP RavB + mTagBFP Lamp1 + AF647 anti rabbit CapZB/original/1432 green far red.tif]

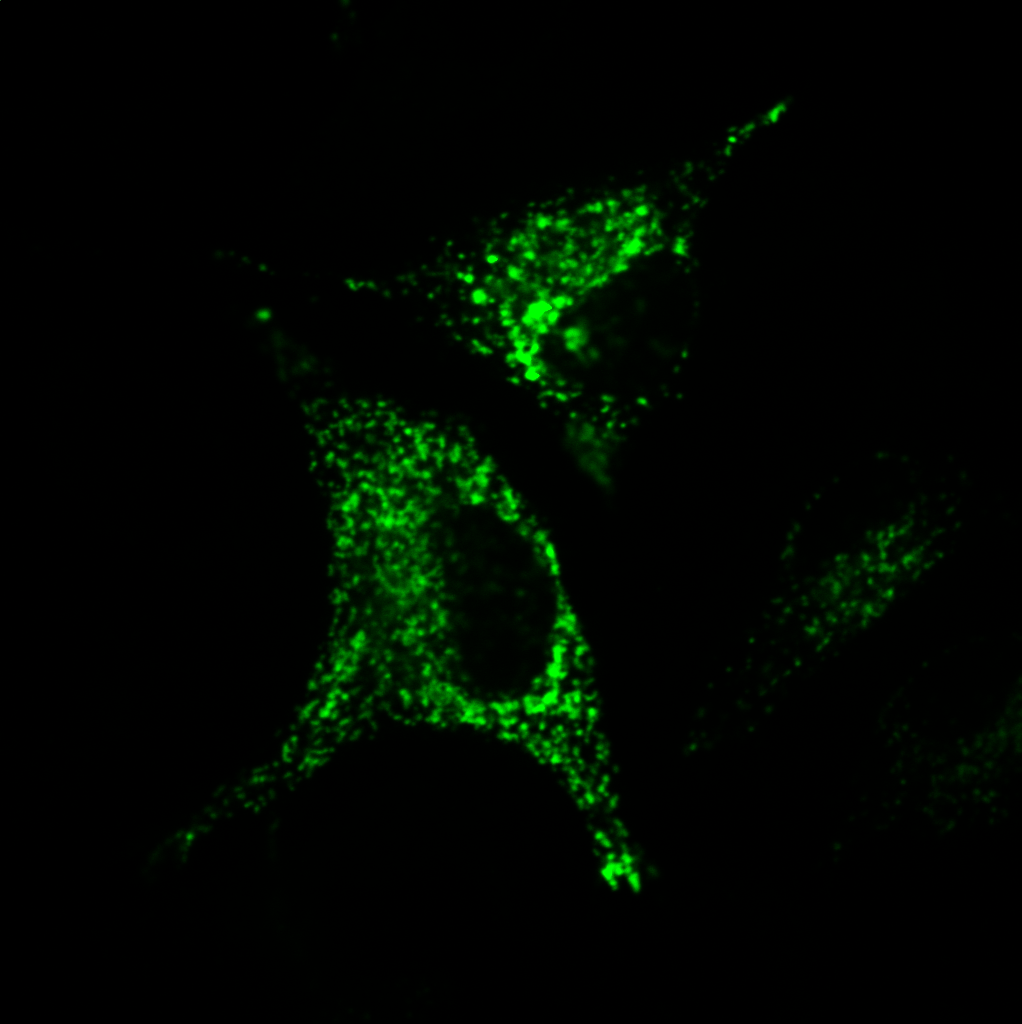

Supplement: Supplementary file 14 — Source data Fig. 5 [file 44318_2025_665_MOESM14_ESM.zip › Figure 5/5a/3RA EGFP RavB + mTagBFP Lamp1 + AF647 anti rabbit CapZB/original/1432 green.tif]

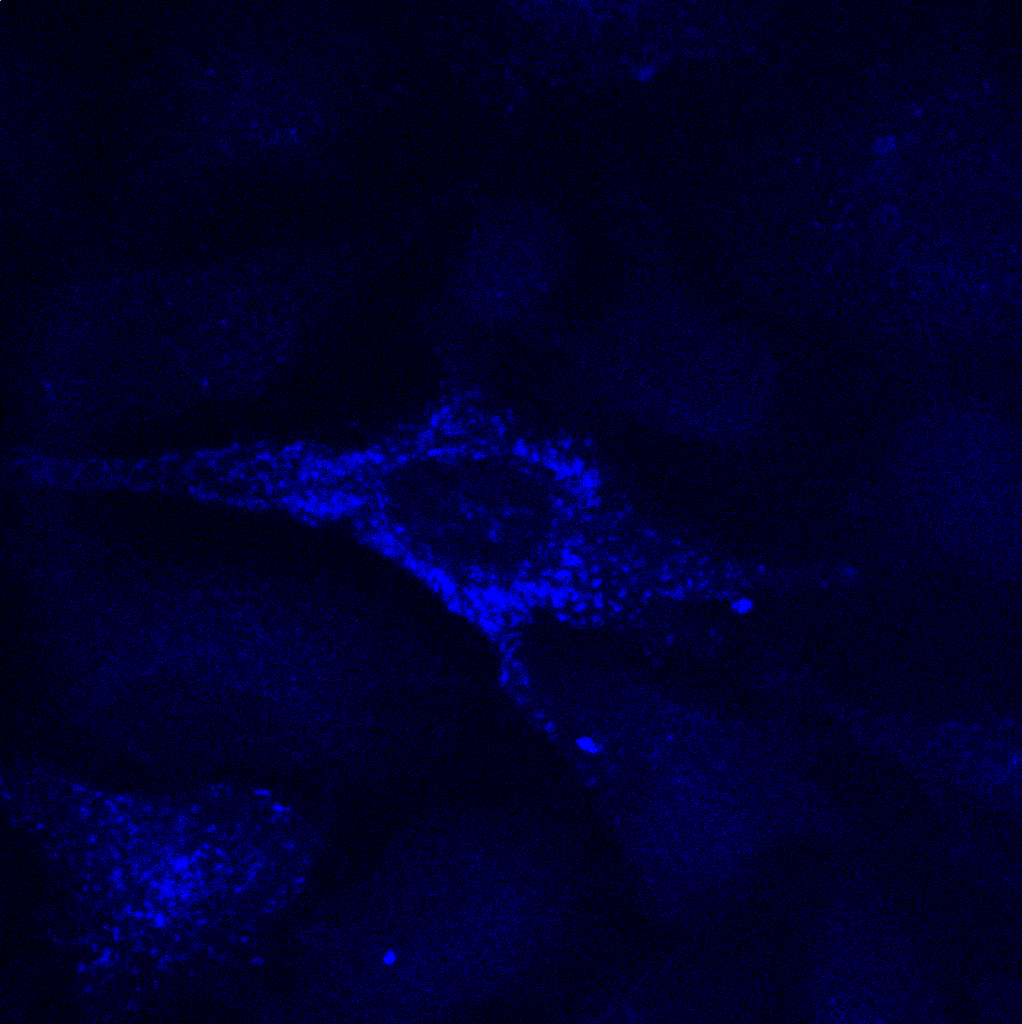

Supplement: Supplementary file 14 — Source data Fig. 5 [file 44318_2025_665_MOESM14_ESM.zip › Figure 5/5a/CTD EGFP RavB + mTagBFP Lamp1 + AF647 anti rabbit CapZB/original/1547 blue.tif]

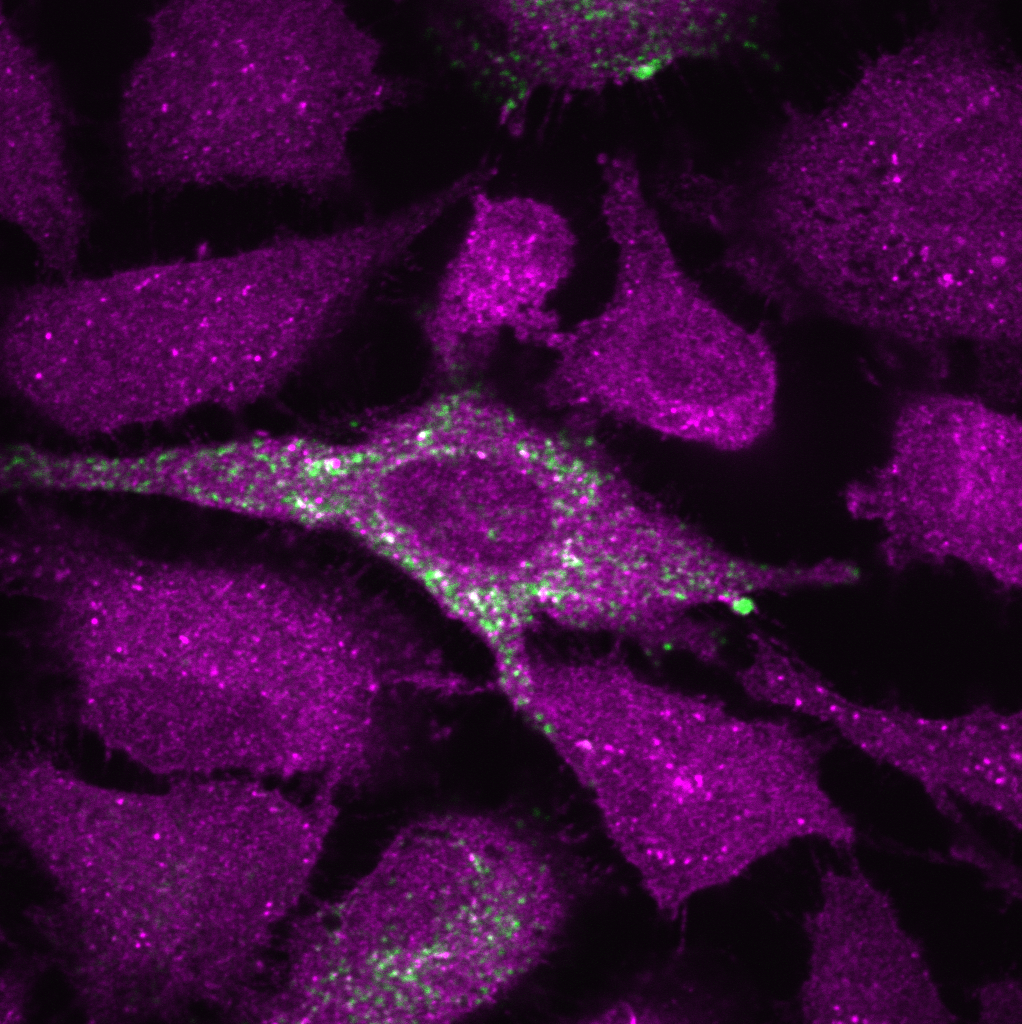

Supplement: Supplementary file 14 — Source data Fig. 5 [file 44318_2025_665_MOESM14_ESM.zip › Figure 5/5a/CTD EGFP RavB + mTagBFP Lamp1 + AF647 anti rabbit CapZB/original/1547 far red green.tif]

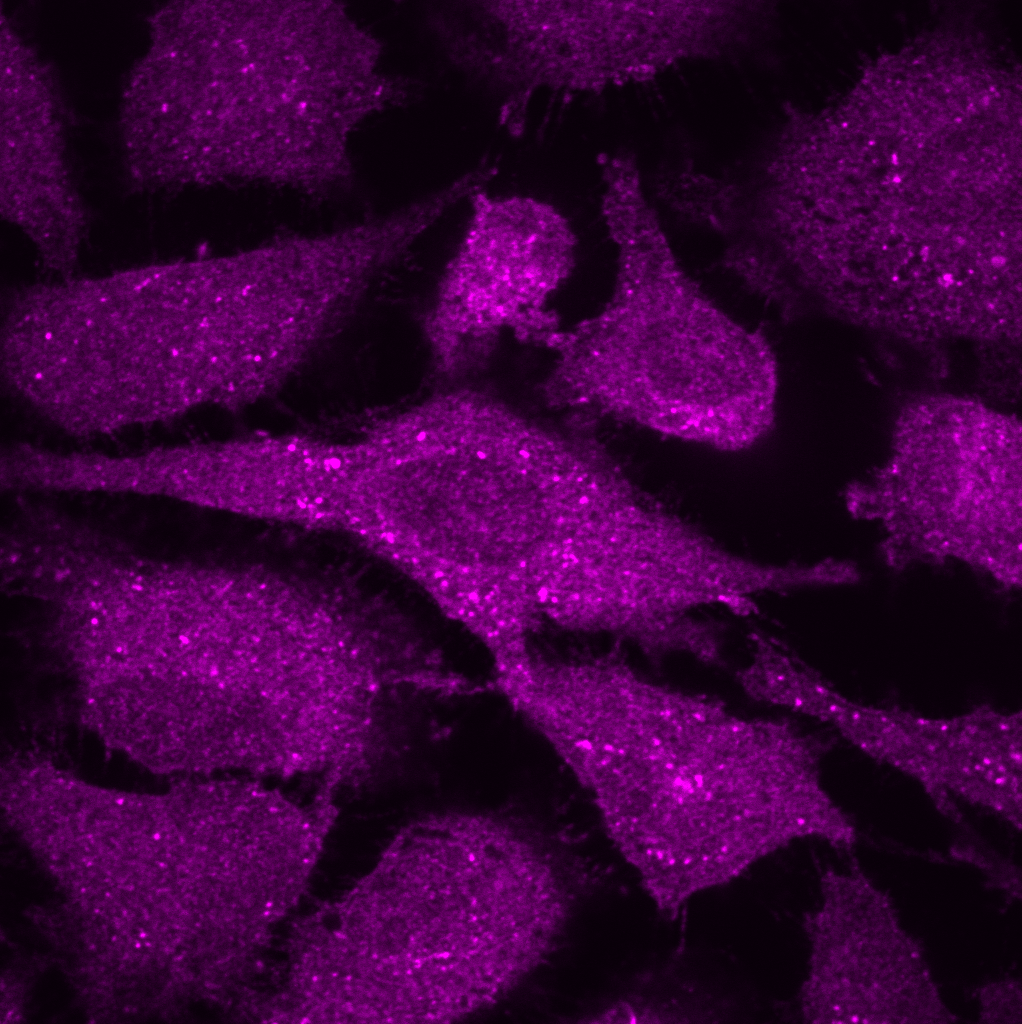

Supplement: Supplementary file 14 — Source data Fig. 5 [file 44318_2025_665_MOESM14_ESM.zip › Figure 5/5a/CTD EGFP RavB + mTagBFP Lamp1 + AF647 anti rabbit CapZB/original/1547 far red.tif]

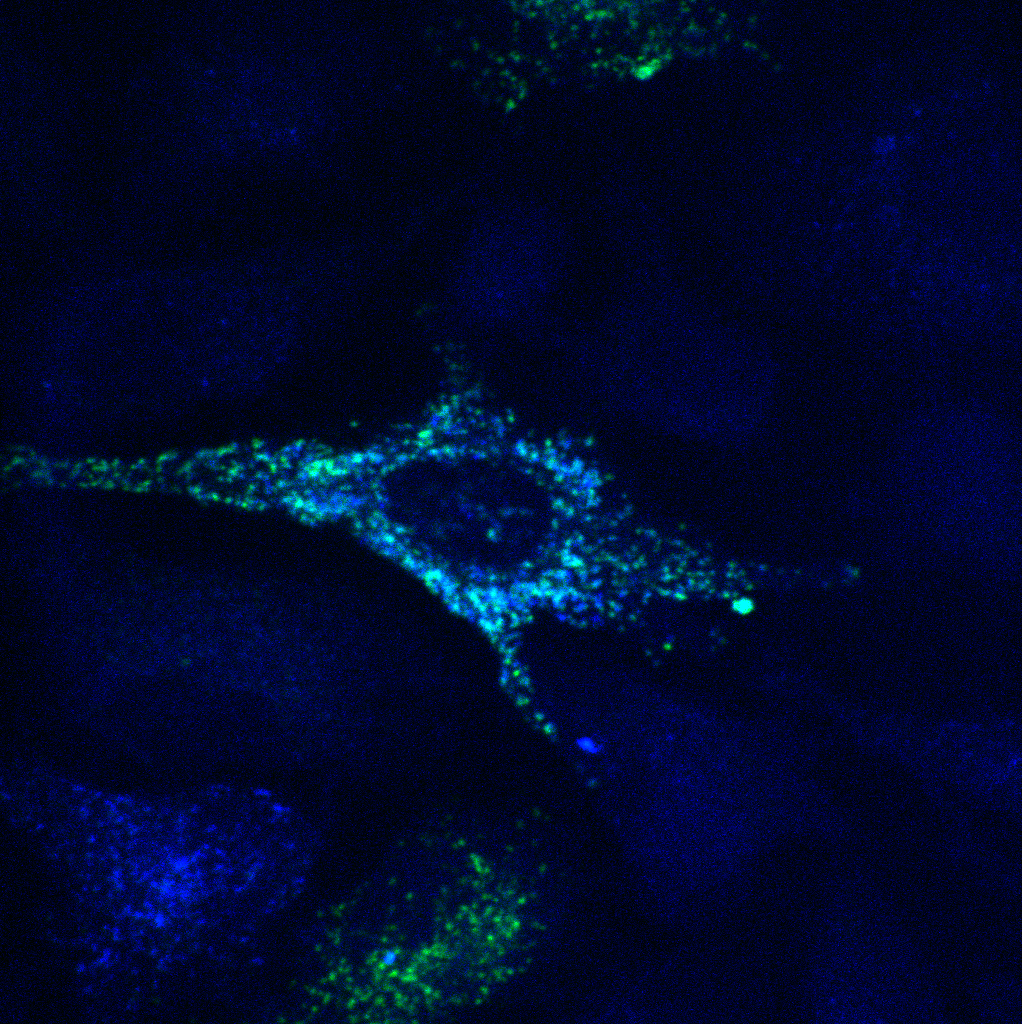

Supplement: Supplementary file 14 — Source data Fig. 5 [file 44318_2025_665_MOESM14_ESM.zip › Figure 5/5a/CTD EGFP RavB + mTagBFP Lamp1 + AF647 anti rabbit CapZB/original/1547 green blue.tif]

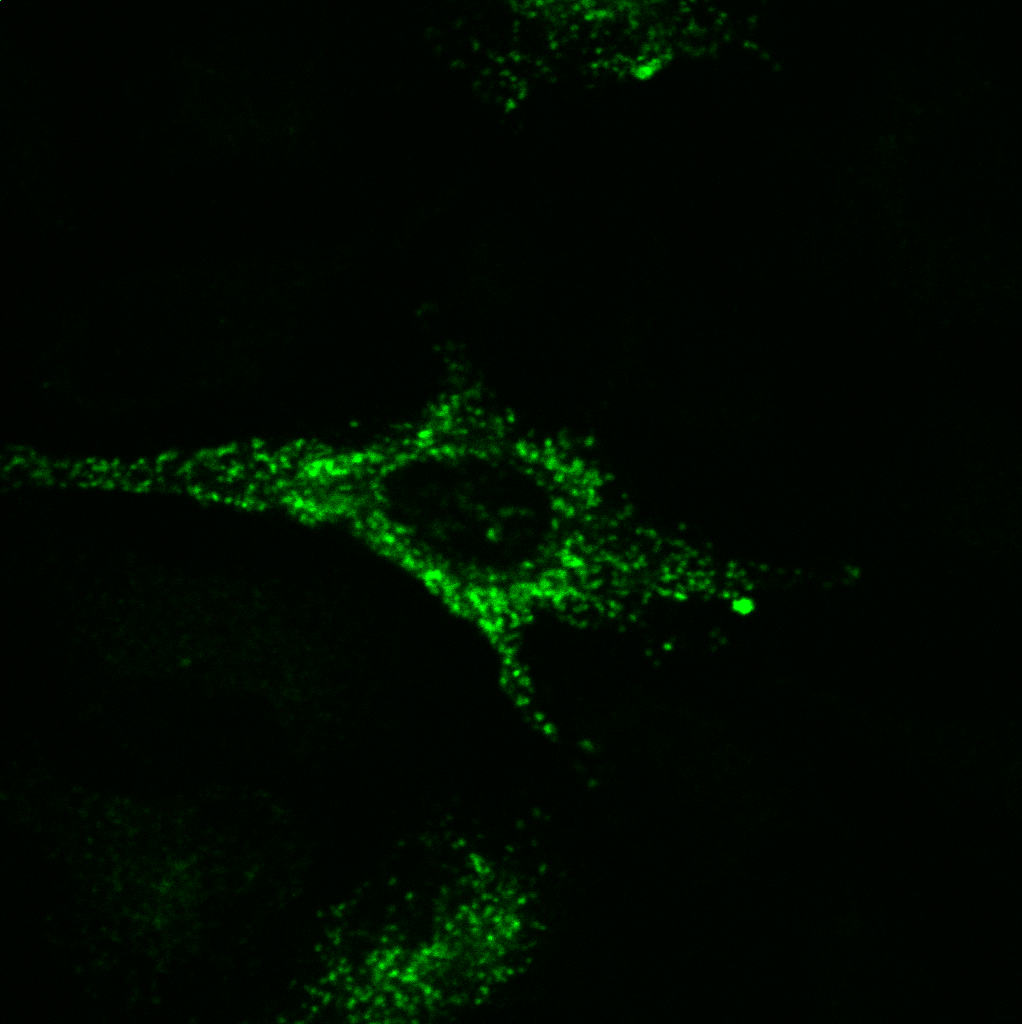

Supplement: Supplementary file 14 — Source data Fig. 5 [file 44318_2025_665_MOESM14_ESM.zip › Figure 5/5a/CTD EGFP RavB + mTagBFP Lamp1 + AF647 anti rabbit CapZB/original/1547 green.tif]

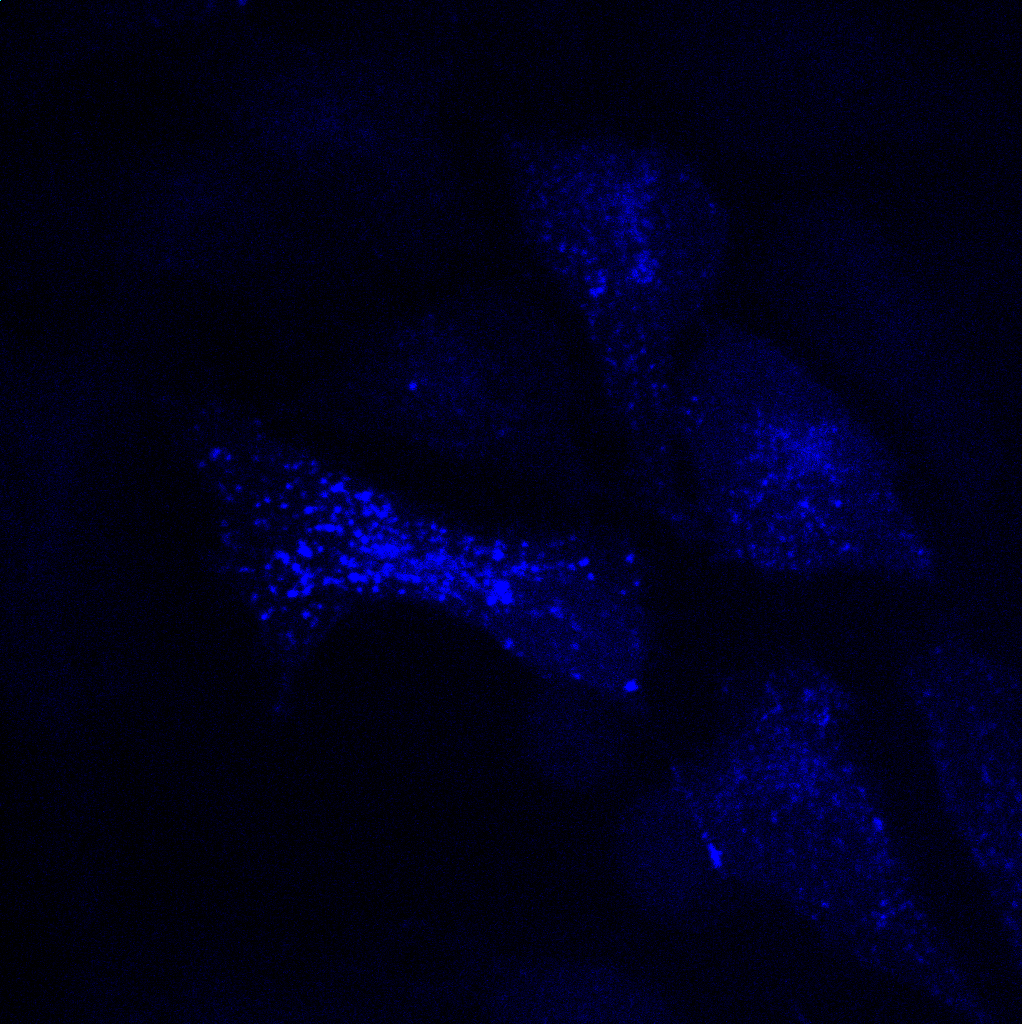

Supplement: Supplementary file 14 — Source data Fig. 5 [file 44318_2025_665_MOESM14_ESM.zip › Figure 5/5a/H206A EGFP RavB + mTagBFP Lamp1 + AF647 anti rabbit CapZB/original/1622 blue.tif]

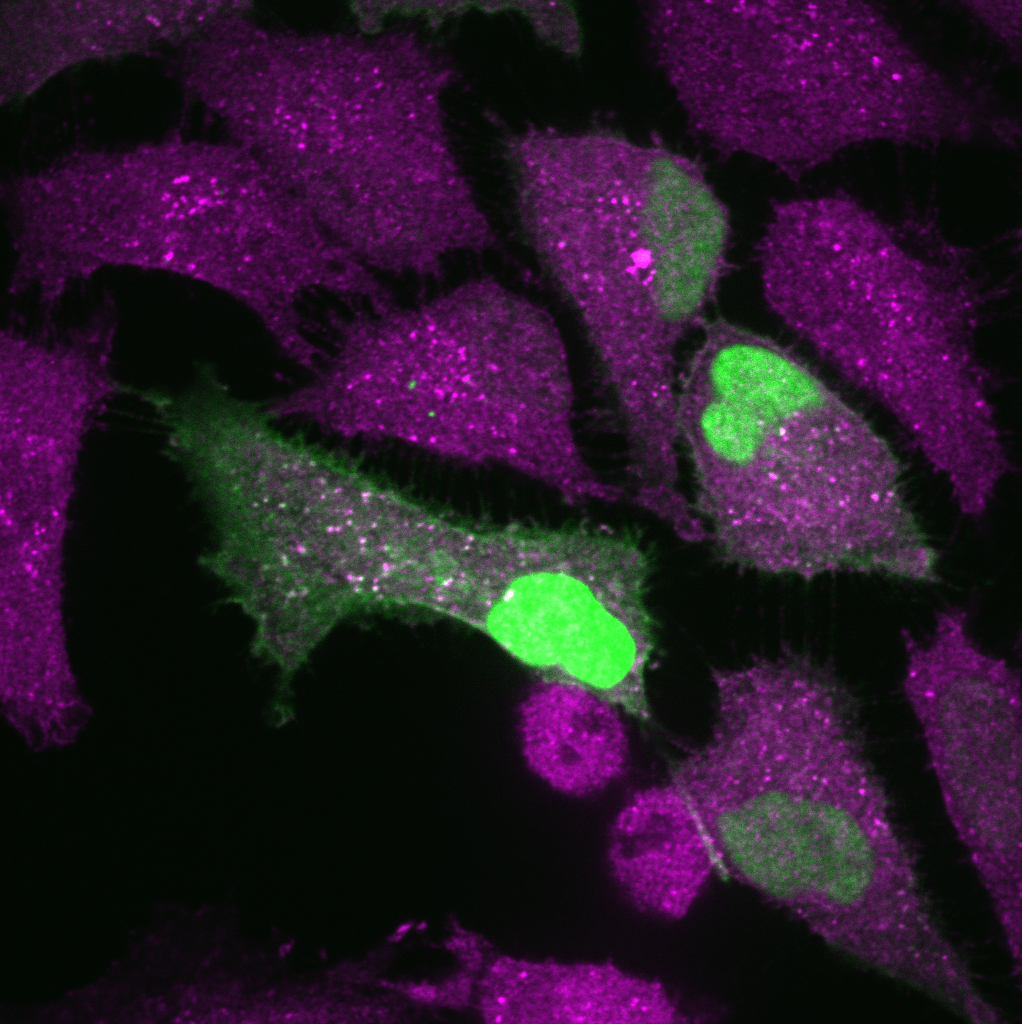

Supplement: Supplementary file 14 — Source data Fig. 5 [file 44318_2025_665_MOESM14_ESM.zip › Figure 5/5a/H206A EGFP RavB + mTagBFP Lamp1 + AF647 anti rabbit CapZB/original/1622 far red green.tif]

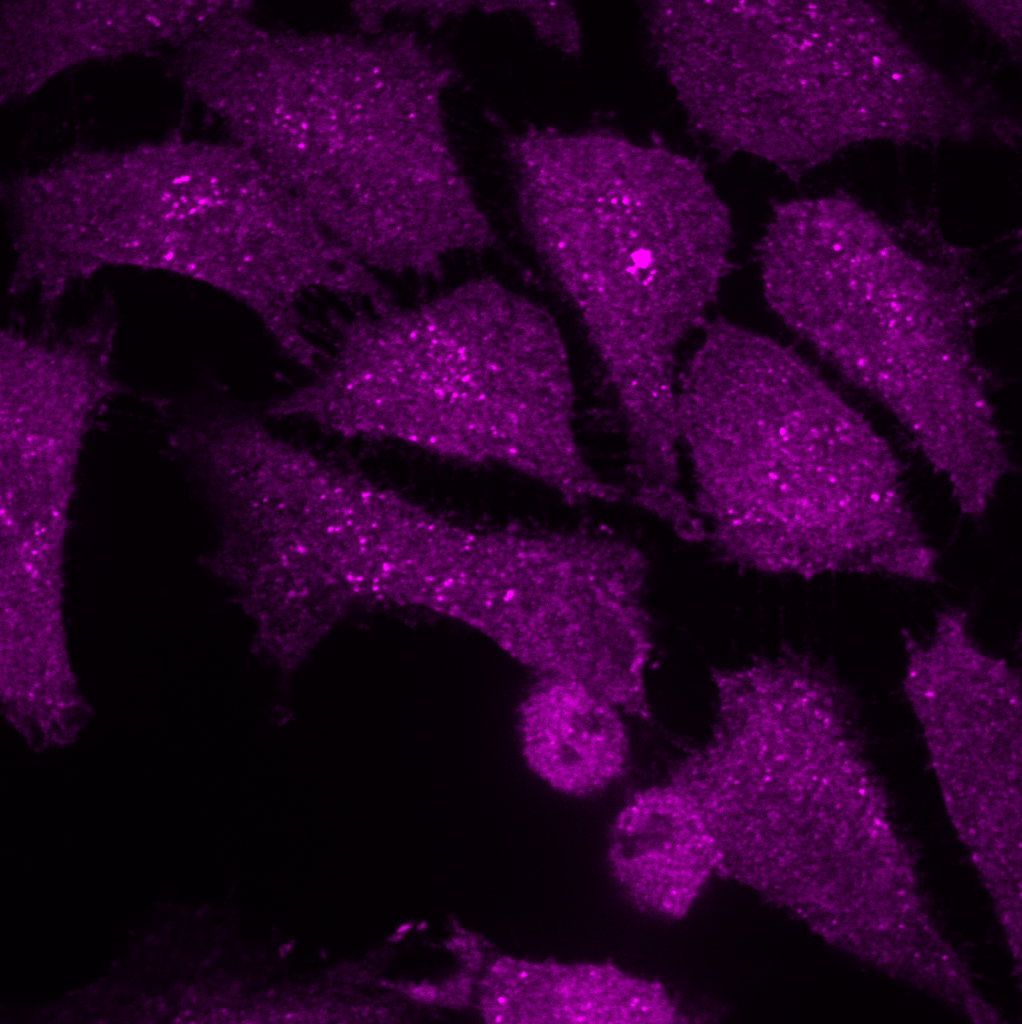

Supplement: Supplementary file 14 — Source data Fig. 5 [file 44318_2025_665_MOESM14_ESM.zip › Figure 5/5a/H206A EGFP RavB + mTagBFP Lamp1 + AF647 anti rabbit CapZB/original/1622 far red.tif]

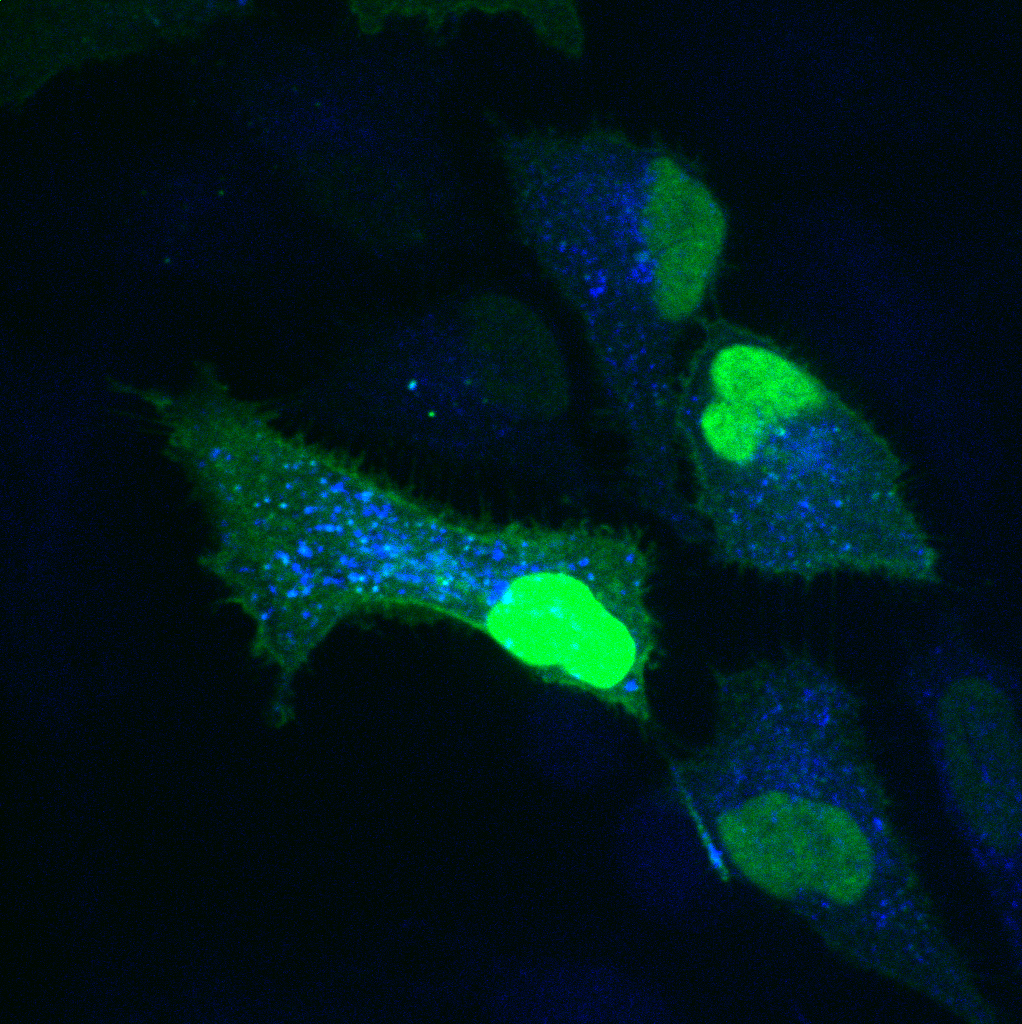

Supplement: Supplementary file 14 — Source data Fig. 5 [file 44318_2025_665_MOESM14_ESM.zip › Figure 5/5a/H206A EGFP RavB + mTagBFP Lamp1 + AF647 anti rabbit CapZB/original/1622 green blue.tif]

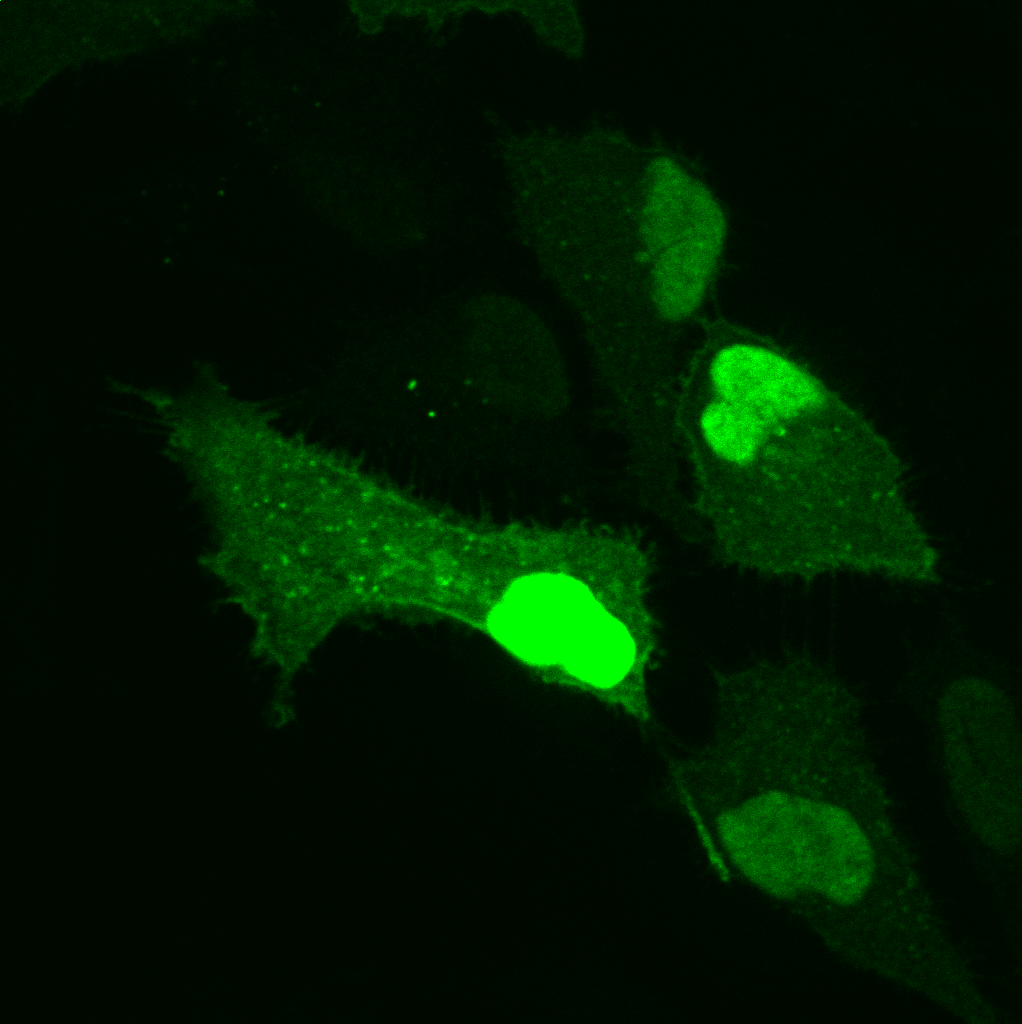

Supplement: Supplementary file 14 — Source data Fig. 5 [file 44318_2025_665_MOESM14_ESM.zip › Figure 5/5a/H206A EGFP RavB + mTagBFP Lamp1 + AF647 anti rabbit CapZB/original/1622 green.tif]

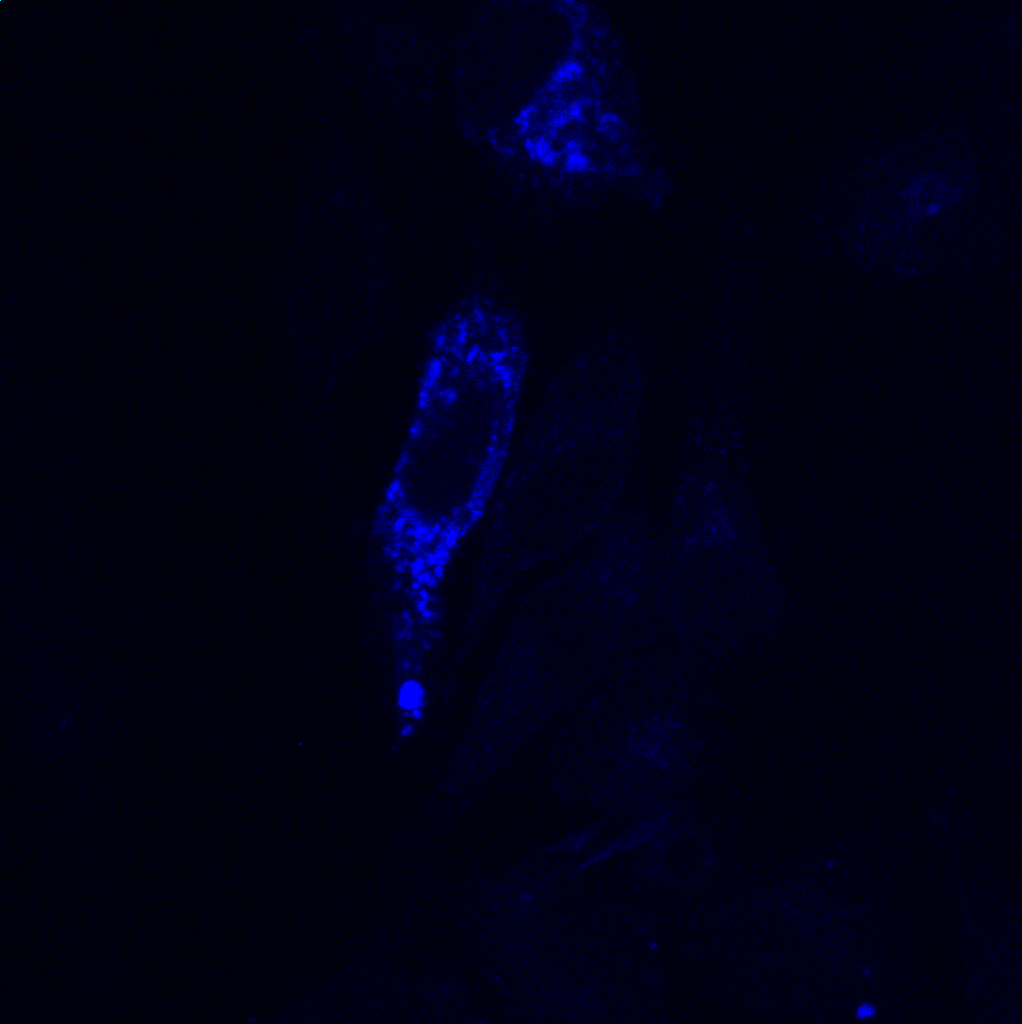

Supplement: Supplementary file 14 — Source data Fig. 5 [file 44318_2025_665_MOESM14_ESM.zip › Figure 5/5a/WT EGFP RavB + mTagBFP Lamp1 + AF647 anti rabbit CapZB/original/1528 blue.tif]

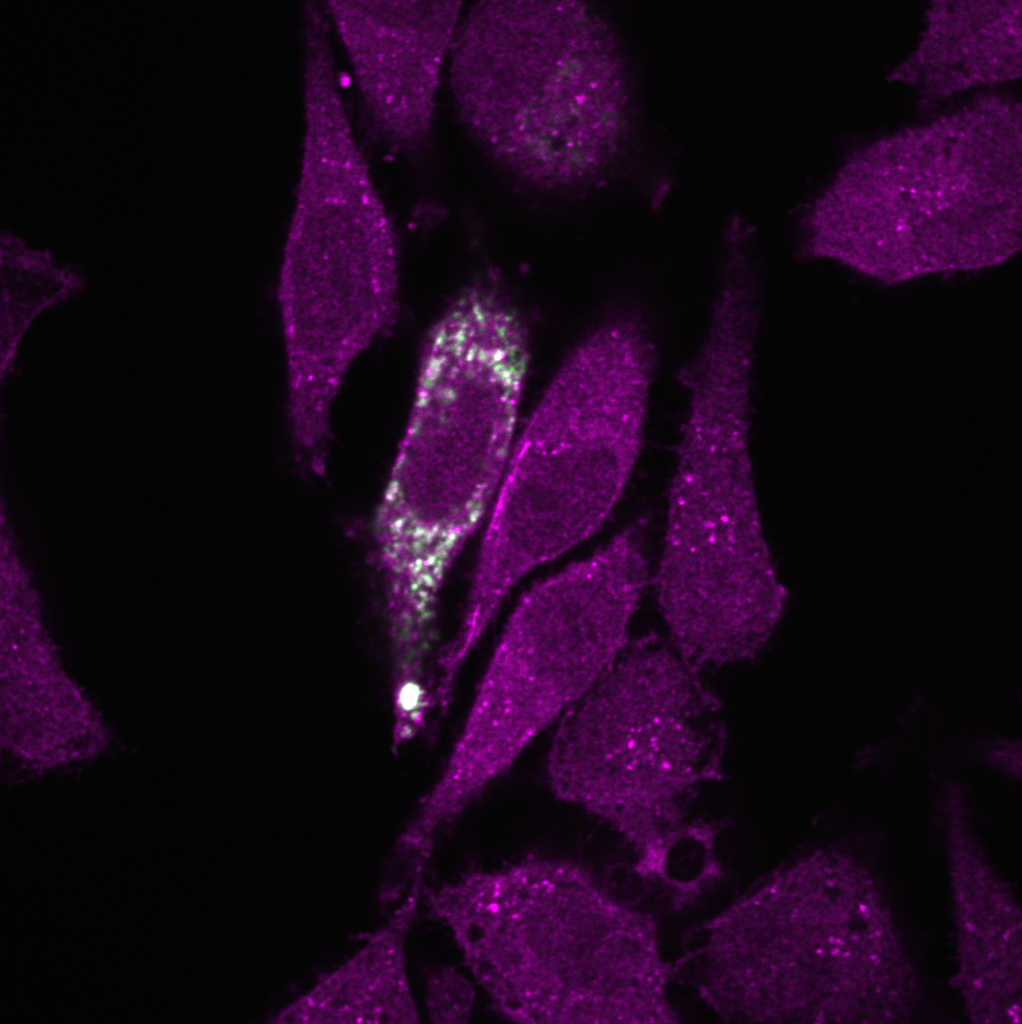

Supplement: Supplementary file 14 — Source data Fig. 5 [file 44318_2025_665_MOESM14_ESM.zip › Figure 5/5a/WT EGFP RavB + mTagBFP Lamp1 + AF647 anti rabbit CapZB/original/1528 far red green.tif]

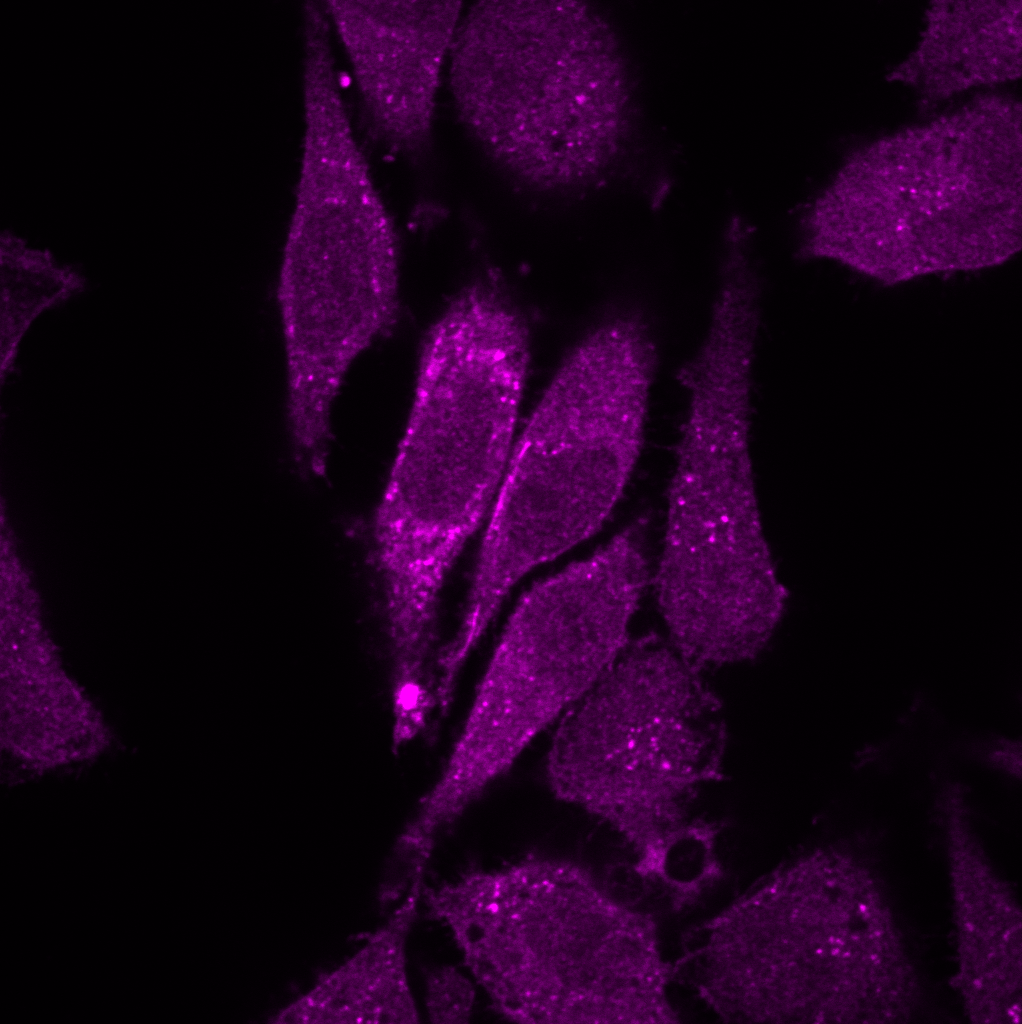

Supplement: Supplementary file 14 — Source data Fig. 5 [file 44318_2025_665_MOESM14_ESM.zip › Figure 5/5a/WT EGFP RavB + mTagBFP Lamp1 + AF647 anti rabbit CapZB/original/1528 far red.tif]

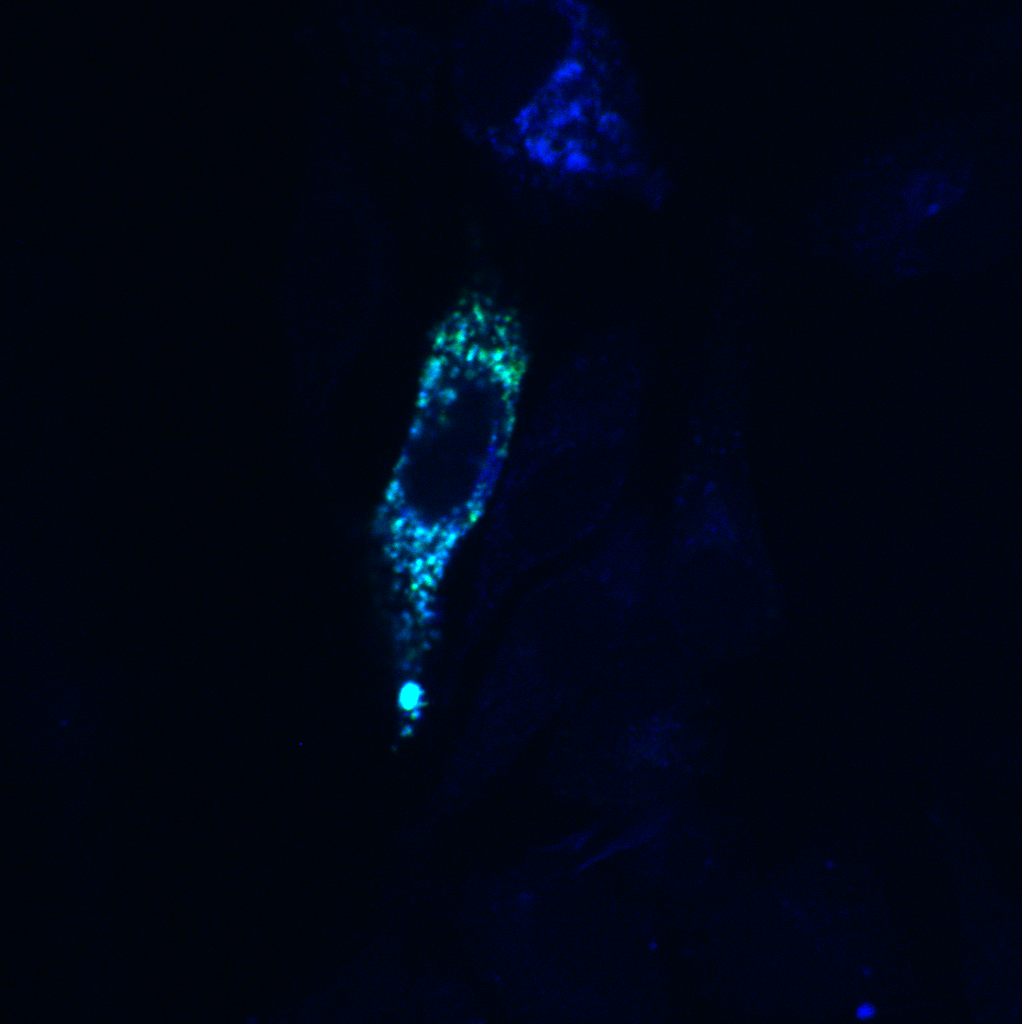

Supplement: Supplementary file 14 — Source data Fig. 5 [file 44318_2025_665_MOESM14_ESM.zip › Figure 5/5a/WT EGFP RavB + mTagBFP Lamp1 + AF647 anti rabbit CapZB/original/1528 green blue.tif]

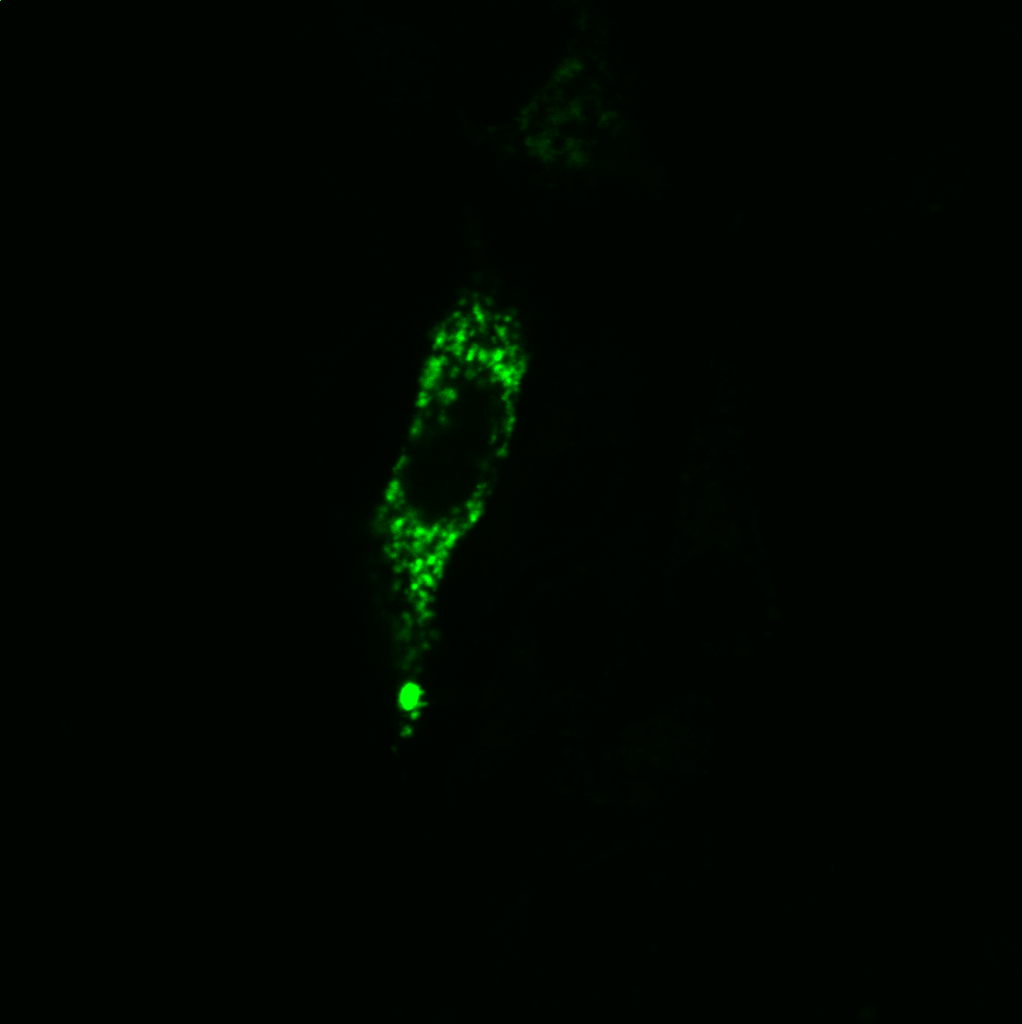

Supplement: Supplementary file 14 — Source data Fig. 5 [file 44318_2025_665_MOESM14_ESM.zip › Figure 5/5a/WT EGFP RavB + mTagBFP Lamp1 + AF647 anti rabbit CapZB/original/1528 green.tif]

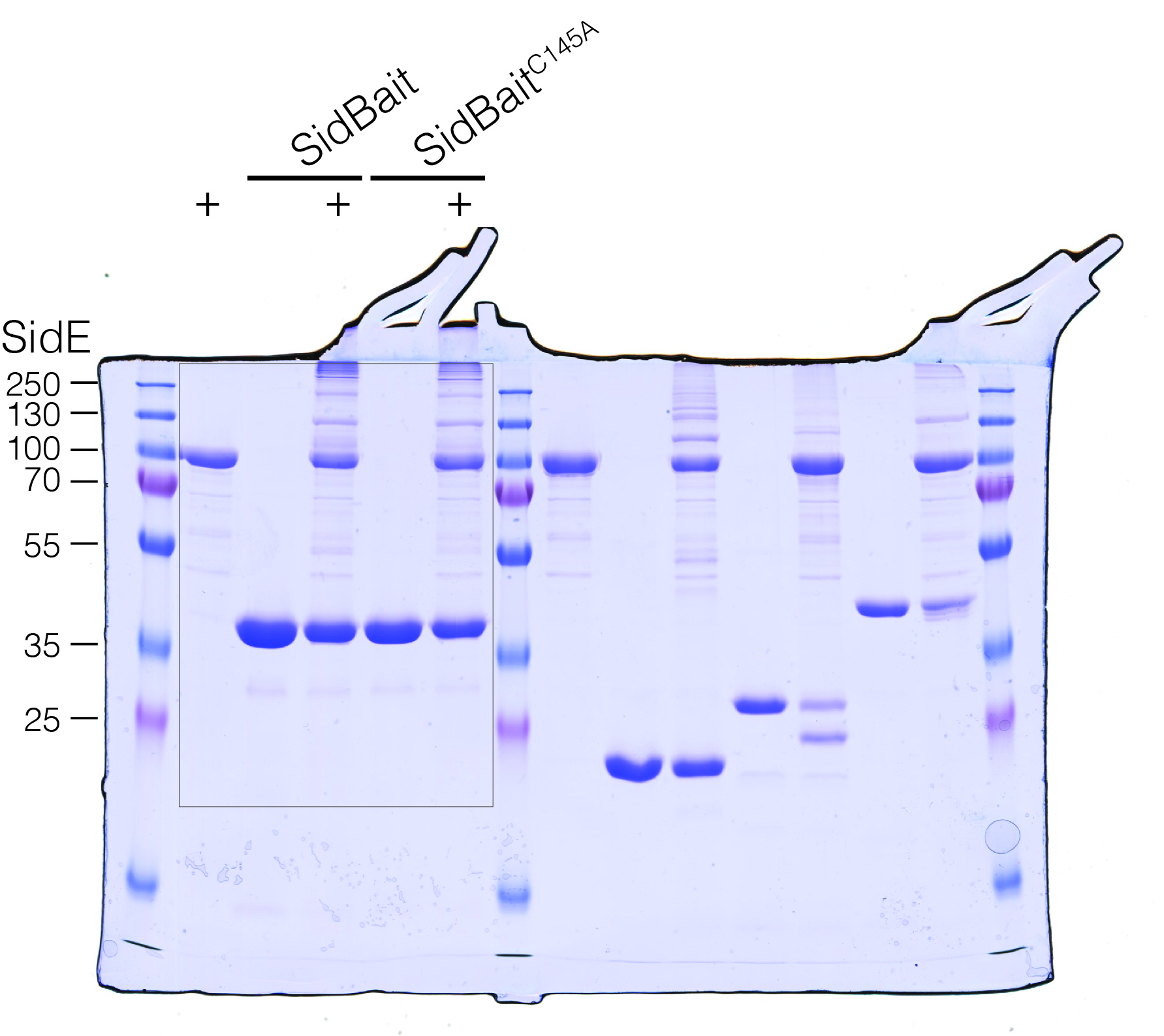

Supplement: Supplementary file 15 — Figure EV1 Source Data [file 44318_2025_665_MOESM15_ESM.zip › Figure EV1/EV1b.jpg]

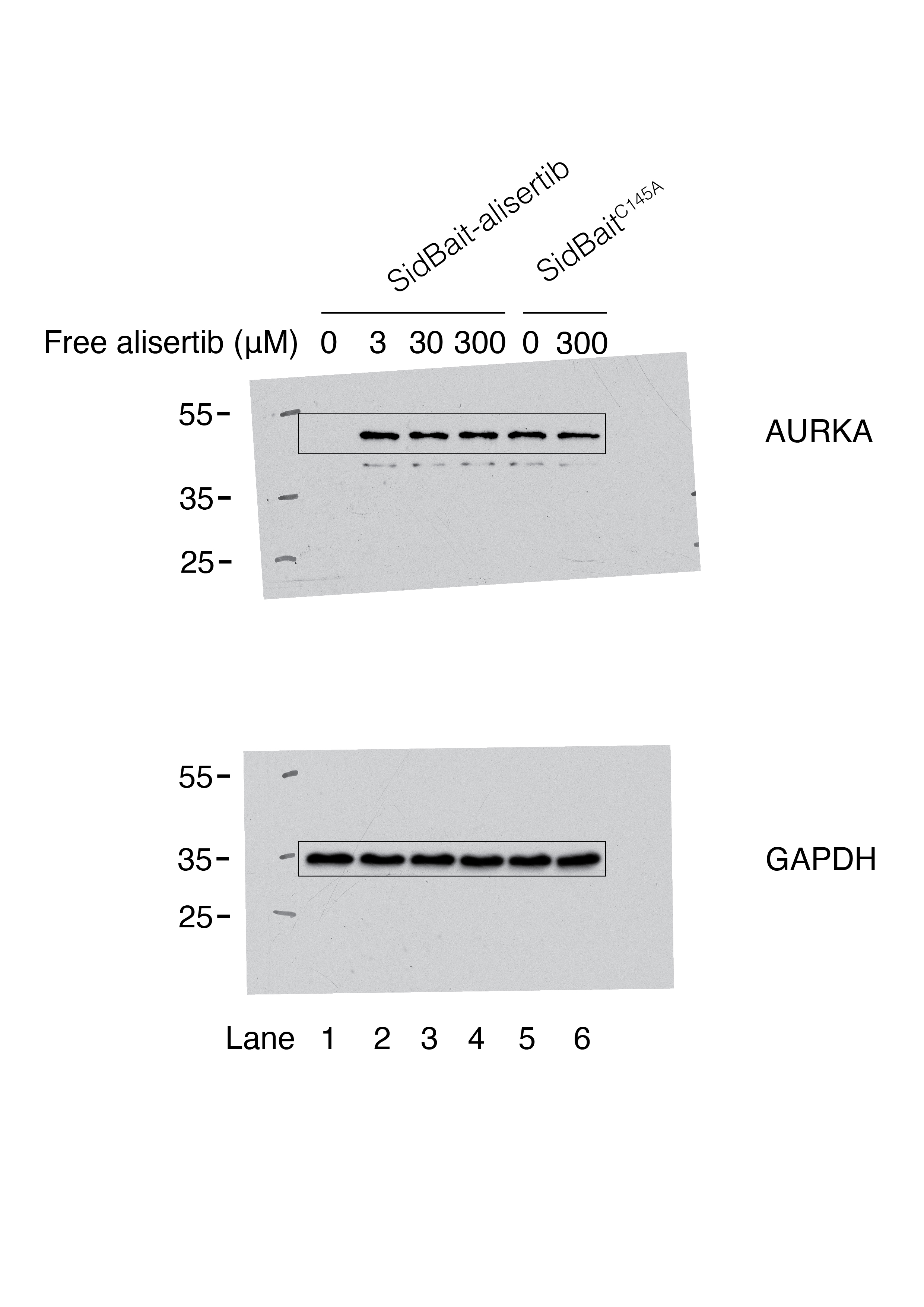

Supplement: Supplementary file 15 — Figure EV1 Source Data [file 44318_2025_665_MOESM15_ESM.zip › Figure EV1/EV1e.jpg]

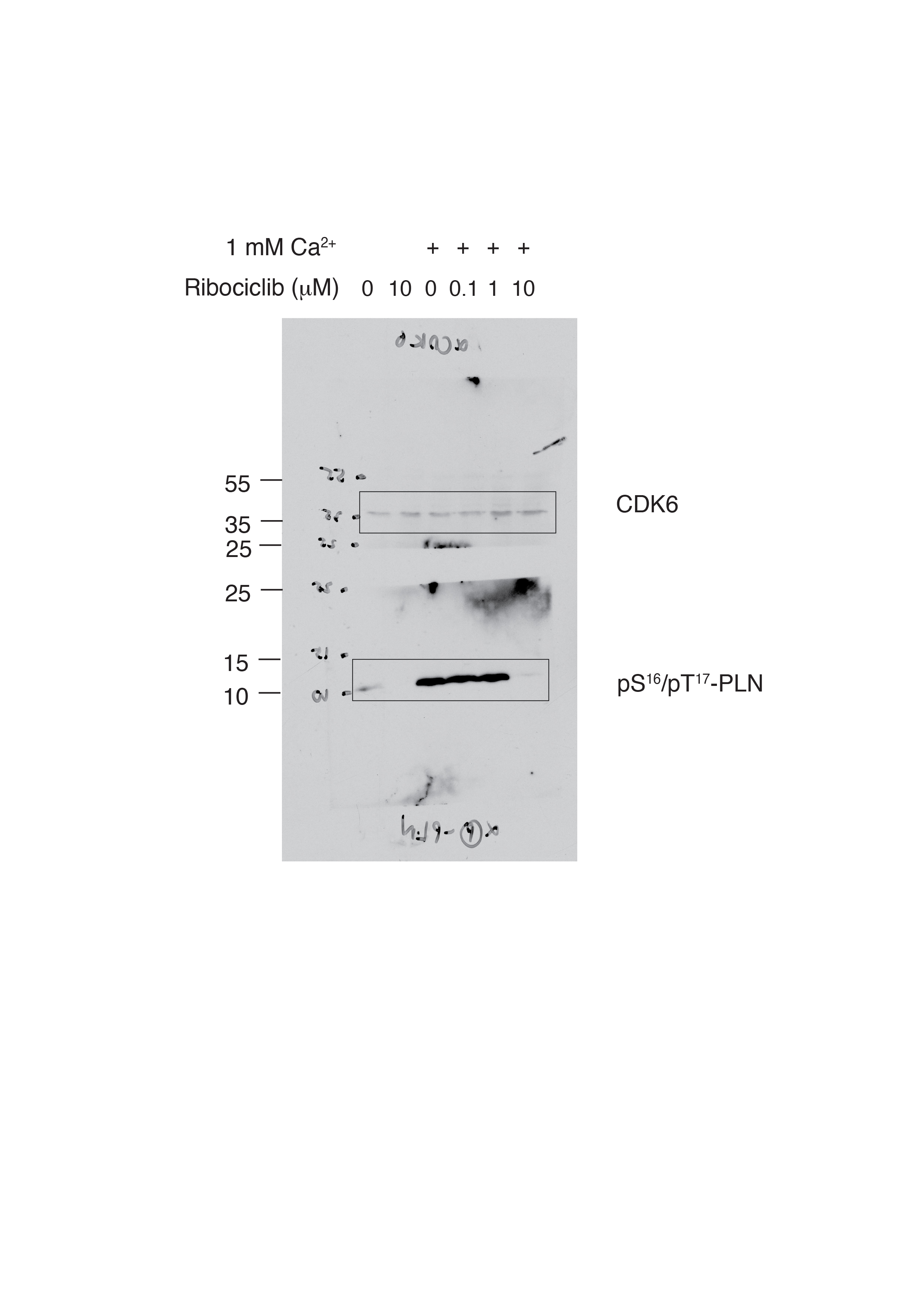

Supplement: Supplementary file 16 — Figure EV3 Source Data [file 44318_2025_665_MOESM16_ESM.zip › Figure EV3/EV3d.jpg]

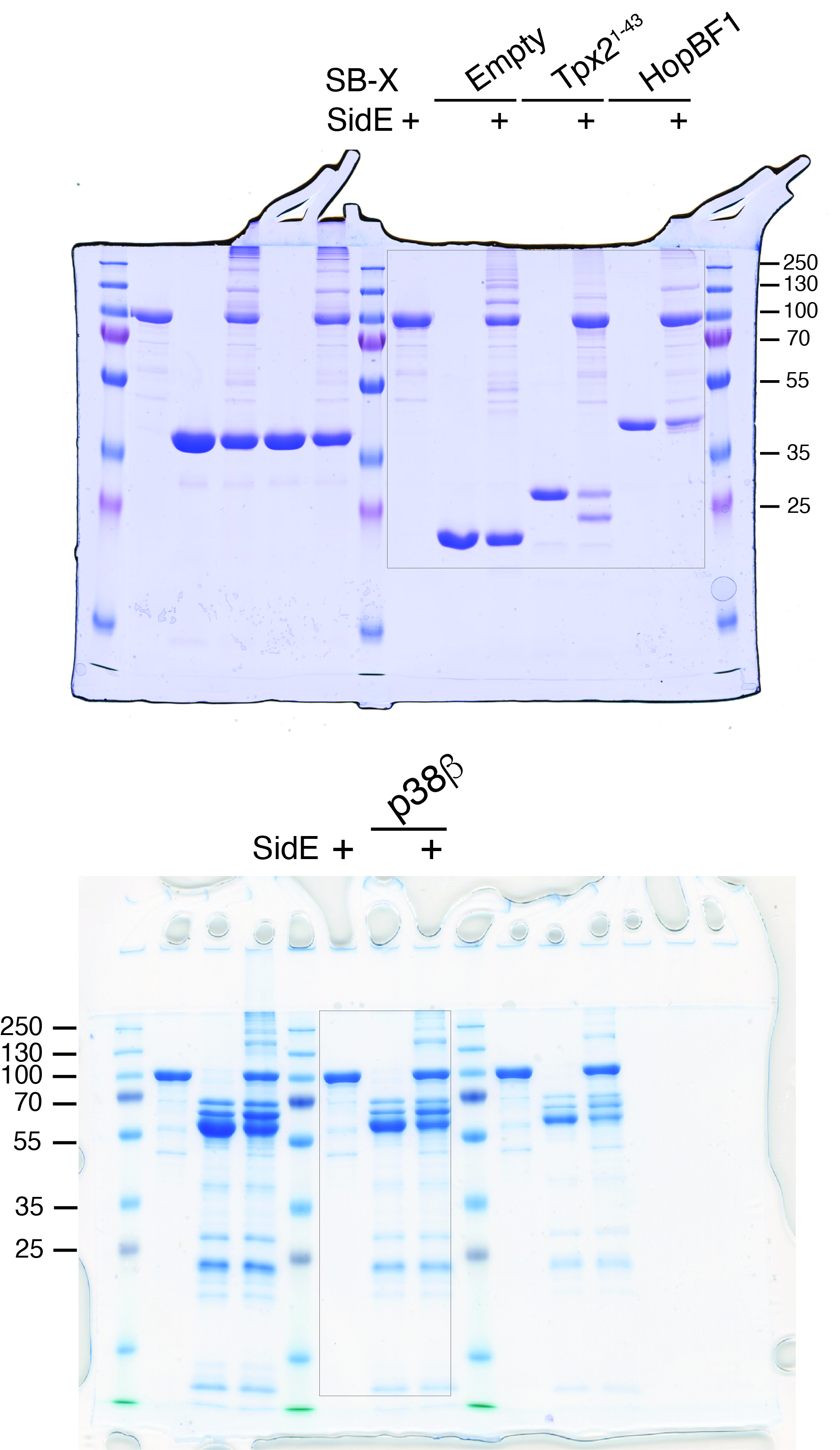

Supplement: Supplementary file 17 — Figure EV4 Source Data [file 44318_2025_665_MOESM17_ESM.zip › Figure EV4/EV4c.jpg]

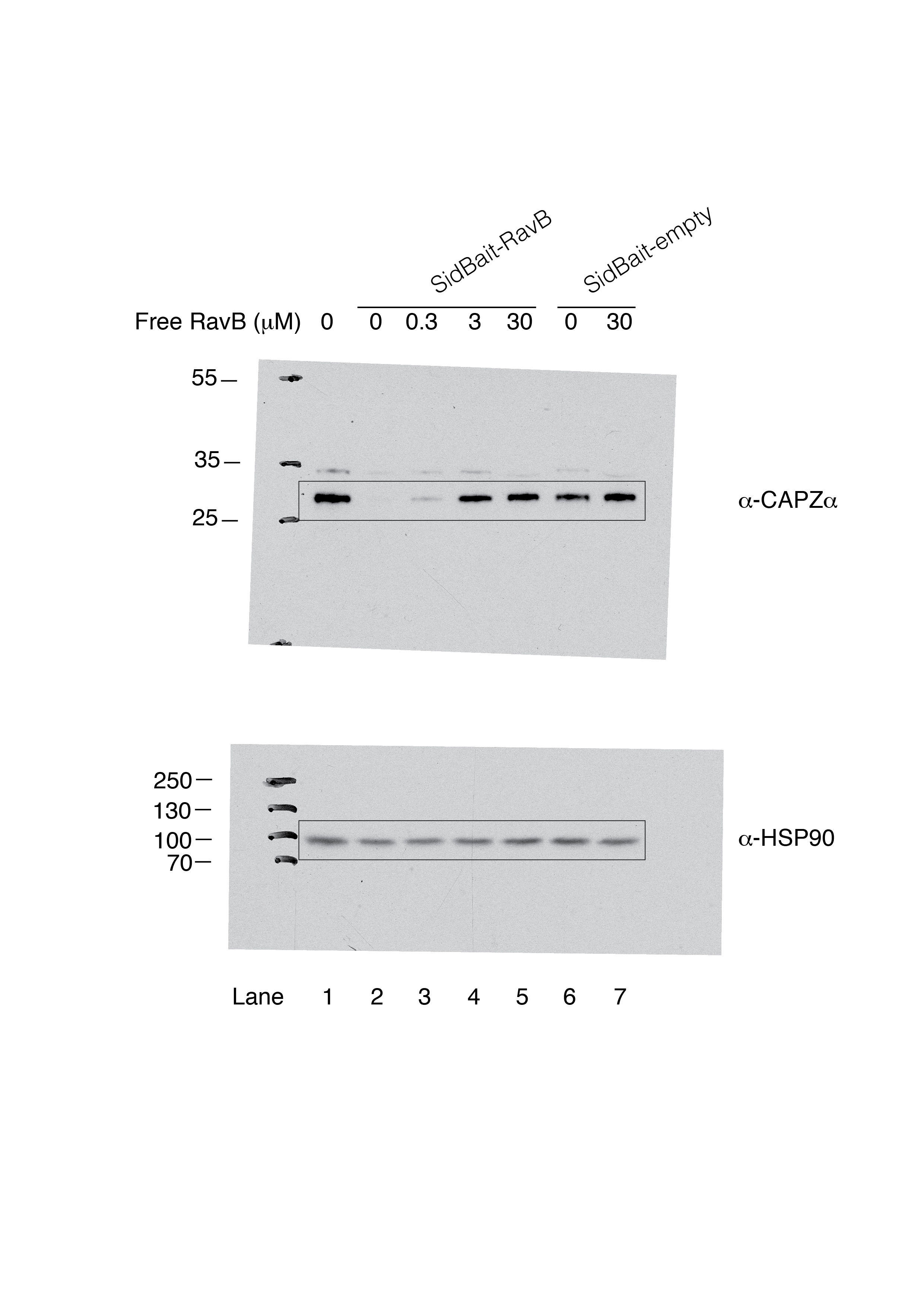

Supplement: Supplementary file 18 — Figure EV5 Source Data [file 44318_2025_665_MOESM18_ESM.zip › Figure EV5/EV5a.jpg]

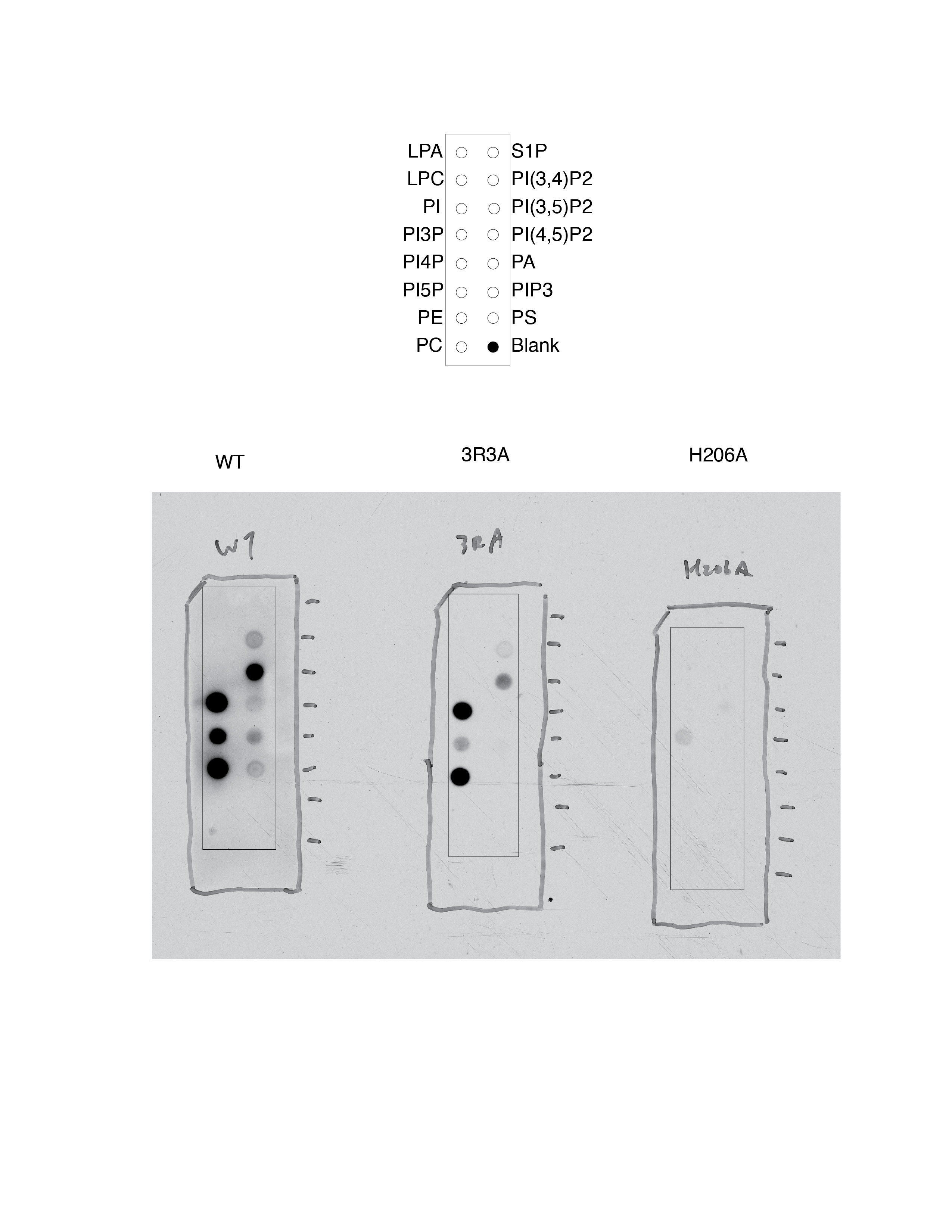

Supplement: Supplementary file 19 — Figure EV6 Source Data [file 44318_2025_665_MOESM19_ESM.zip › Figure EV6/EV6c.jpg]

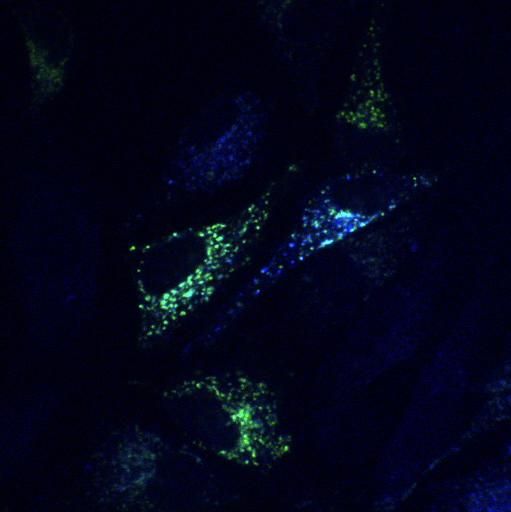

Supplement: Supplementary file 19 — Figure EV6 Source Data [file 44318_2025_665_MOESM19_ESM.zip › Figure EV6/EV6a/3RA EGFP RavB + mTagBFP Lamp1 + AF647 anti rabbit CapZB/100x EMCCD snap_1433.vsi]

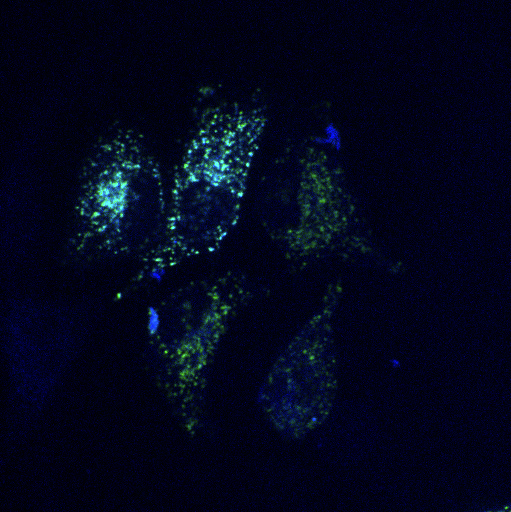

Supplement: Supplementary file 19 — Figure EV6 Source Data [file 44318_2025_665_MOESM19_ESM.zip › Figure EV6/EV6a/3RA EGFP RavB + mTagBFP Lamp1 + AF647 anti rabbit CapZB/100x EMCCD snap_1435.vsi]

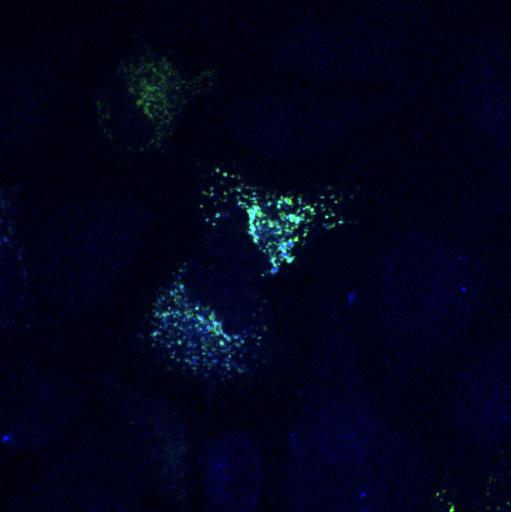

Supplement: Supplementary file 19 — Figure EV6 Source Data [file 44318_2025_665_MOESM19_ESM.zip › Figure EV6/EV6a/3RA EGFP RavB + mTagBFP Lamp1 + AF647 anti rabbit CapZB/100x EMCCD snap_1435_01.vsi]

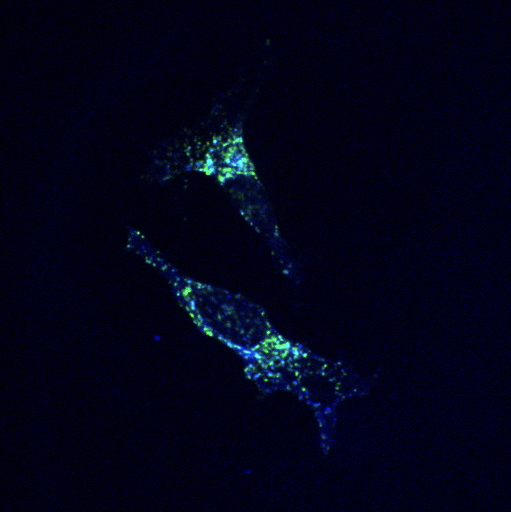

Supplement: Supplementary file 19 — Figure EV6 Source Data [file 44318_2025_665_MOESM19_ESM.zip › Figure EV6/EV6a/3RA EGFP RavB + mTagBFP Lamp1 + AF647 anti rabbit CapZB/100x EMCCD snap_1449.vsi]

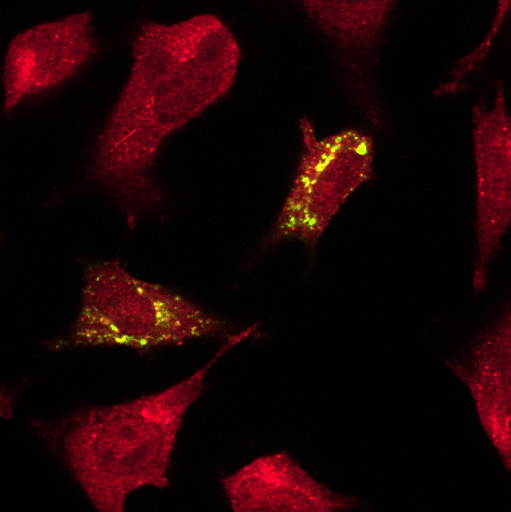

Supplement: Supplementary file 19 — Figure EV6 Source Data [file 44318_2025_665_MOESM19_ESM.zip › Figure EV6/EV6a/CTD EGFP RavB + mTagBFP Lamp1 + AF647 anti rabbit CapZB/100x EMCCD snap_1546.vsi]

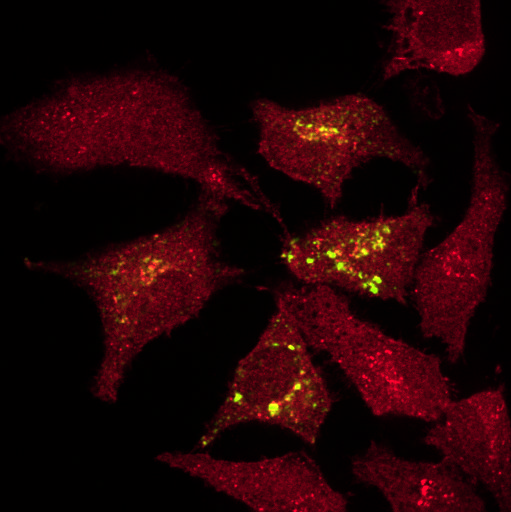

Supplement: Supplementary file 19 — Figure EV6 Source Data [file 44318_2025_665_MOESM19_ESM.zip › Figure EV6/EV6a/CTD EGFP RavB + mTagBFP Lamp1 + AF647 anti rabbit CapZB/100x EMCCD snap_1604.vsi]

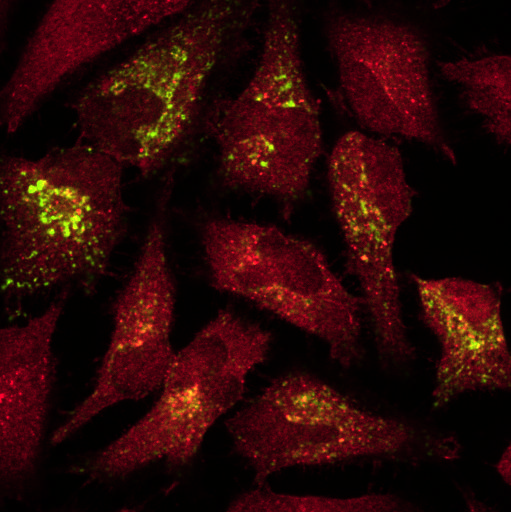

Supplement: Supplementary file 19 — Figure EV6 Source Data [file 44318_2025_665_MOESM19_ESM.zip › Figure EV6/EV6a/CTD EGFP RavB + mTagBFP Lamp1 + AF647 anti rabbit CapZB/100x EMCCD snap_1605.vsi]

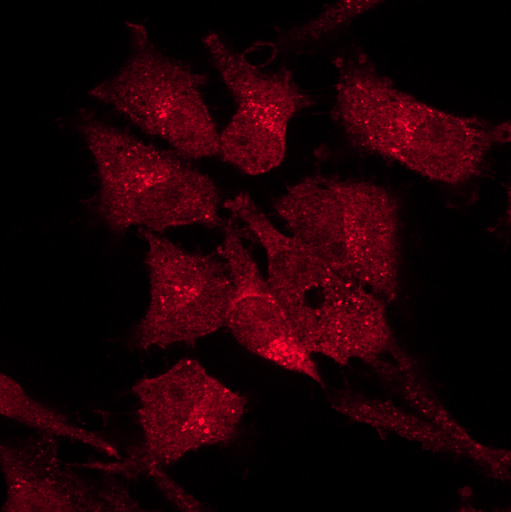

Supplement: Supplementary file 19 — Figure EV6 Source Data [file 44318_2025_665_MOESM19_ESM.zip › Figure EV6/EV6a/CTD EGFP RavB + mTagBFP Lamp1 + AF647 anti rabbit CapZB/100x EMCCD snap_1605_01.vsi]

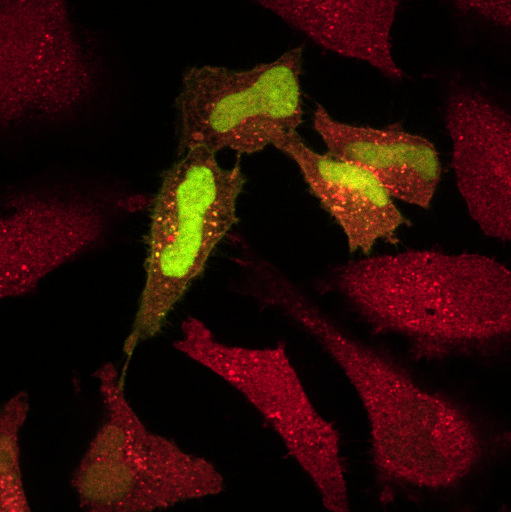

Supplement: Supplementary file 19 — Figure EV6 Source Data [file 44318_2025_665_MOESM19_ESM.zip › Figure EV6/EV6a/H206A EGFP RavB + mTagBFP Lamp1 + AF647 anti rabbit CapZB/100x EMCCD snap_1610.vsi]

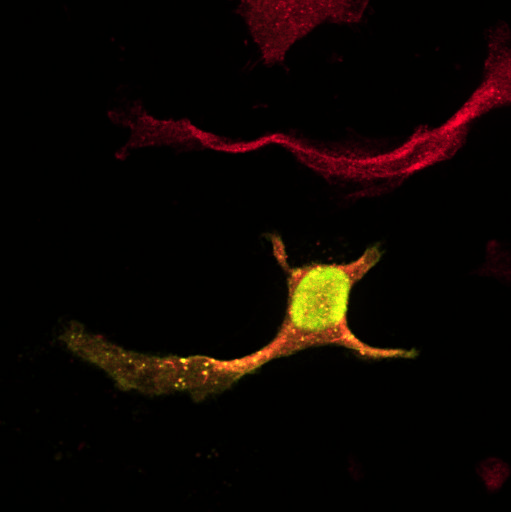

Supplement: Supplementary file 19 — Figure EV6 Source Data [file 44318_2025_665_MOESM19_ESM.zip › Figure EV6/EV6a/H206A EGFP RavB + mTagBFP Lamp1 + AF647 anti rabbit CapZB/100x EMCCD snap_1613.vsi]

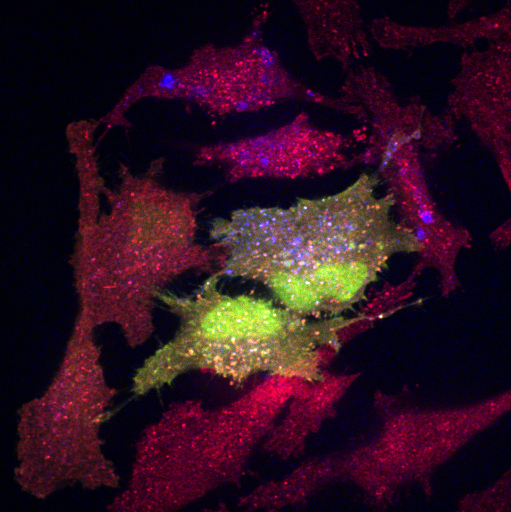

Supplement: Supplementary file 19 — Figure EV6 Source Data [file 44318_2025_665_MOESM19_ESM.zip › Figure EV6/EV6a/H206A EGFP RavB + mTagBFP Lamp1 + AF647 anti rabbit CapZB/100x EMCCD snap_1614.vsi]

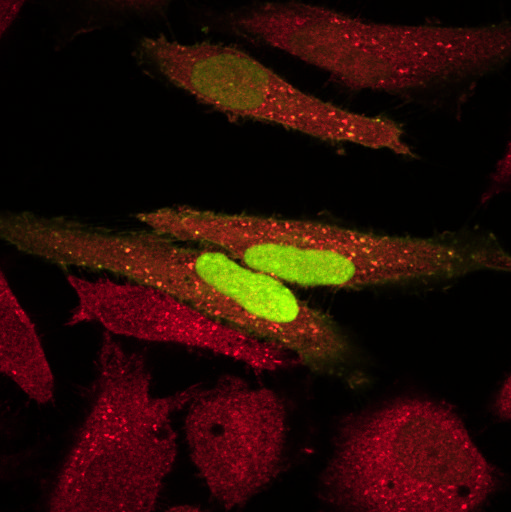

Supplement: Supplementary file 19 — Figure EV6 Source Data [file 44318_2025_665_MOESM19_ESM.zip › Figure EV6/EV6a/H206A EGFP RavB + mTagBFP Lamp1 + AF647 anti rabbit CapZB/100x EMCCD snap_1624.vsi]

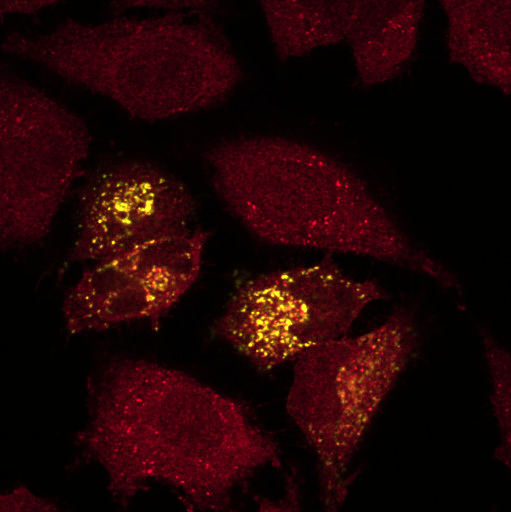

Supplement: Supplementary file 19 — Figure EV6 Source Data [file 44318_2025_665_MOESM19_ESM.zip › Figure EV6/EV6a/WT EGFP RavB + mTagBFP Lamp1 + AF647 anti rabbit CapZB/100x EMCCD snap_1512.vsi]

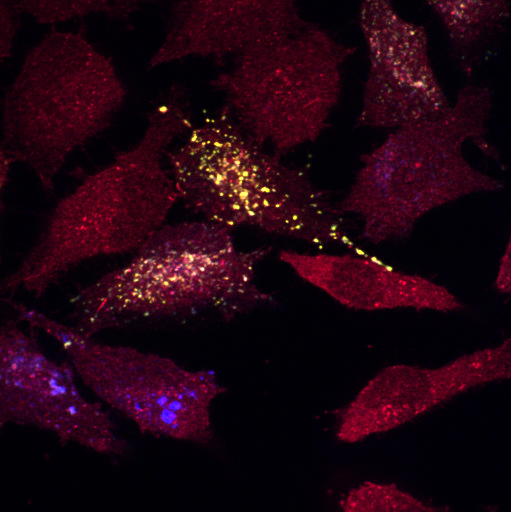

Supplement: Supplementary file 19 — Figure EV6 Source Data [file 44318_2025_665_MOESM19_ESM.zip › Figure EV6/EV6a/WT EGFP RavB + mTagBFP Lamp1 + AF647 anti rabbit CapZB/100x EMCCD snap_1516.vsi]

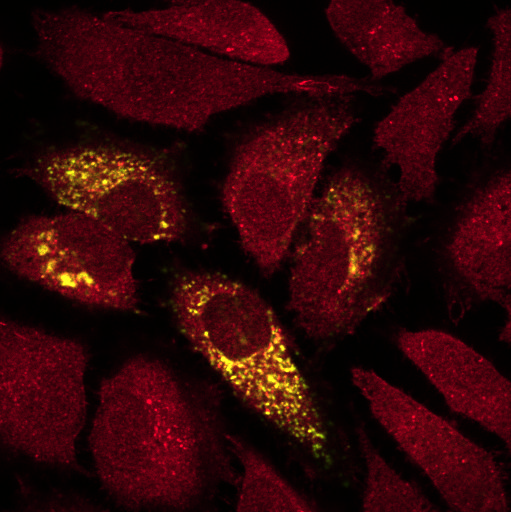

Supplement: Supplementary file 19 — Figure EV6 Source Data [file 44318_2025_665_MOESM19_ESM.zip › Figure EV6/EV6a/WT EGFP RavB + mTagBFP Lamp1 + AF647 anti rabbit CapZB/100x EMCCD snap_1518.vsi]

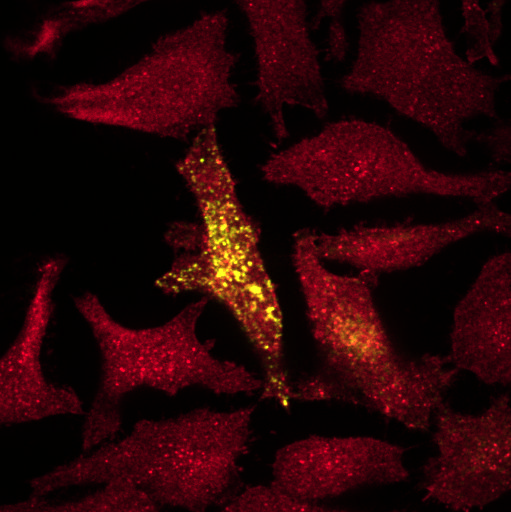

Supplement: Supplementary file 19 — Figure EV6 Source Data [file 44318_2025_665_MOESM19_ESM.zip › Figure EV6/EV6a/WT EGFP RavB + mTagBFP Lamp1 + AF647 anti rabbit CapZB/100x EMCCD snap_1520.vsi]
