# Supplementary material for: Bacterial ubiquitin ligase engineered for small molecule and protein target identification
Source: EMBO J. 2026 Jan 3;45(3):1024–50. doi: 10.1038/s44318-025-00665-0 (PMC12865202; doi:10.1038/s44318-025-00665-0)
Supplement: Supplementary file 20 — Expanded View Figures [file 44318_2025_665_MOESM20_ESM.pdf]

Expanded View Figures

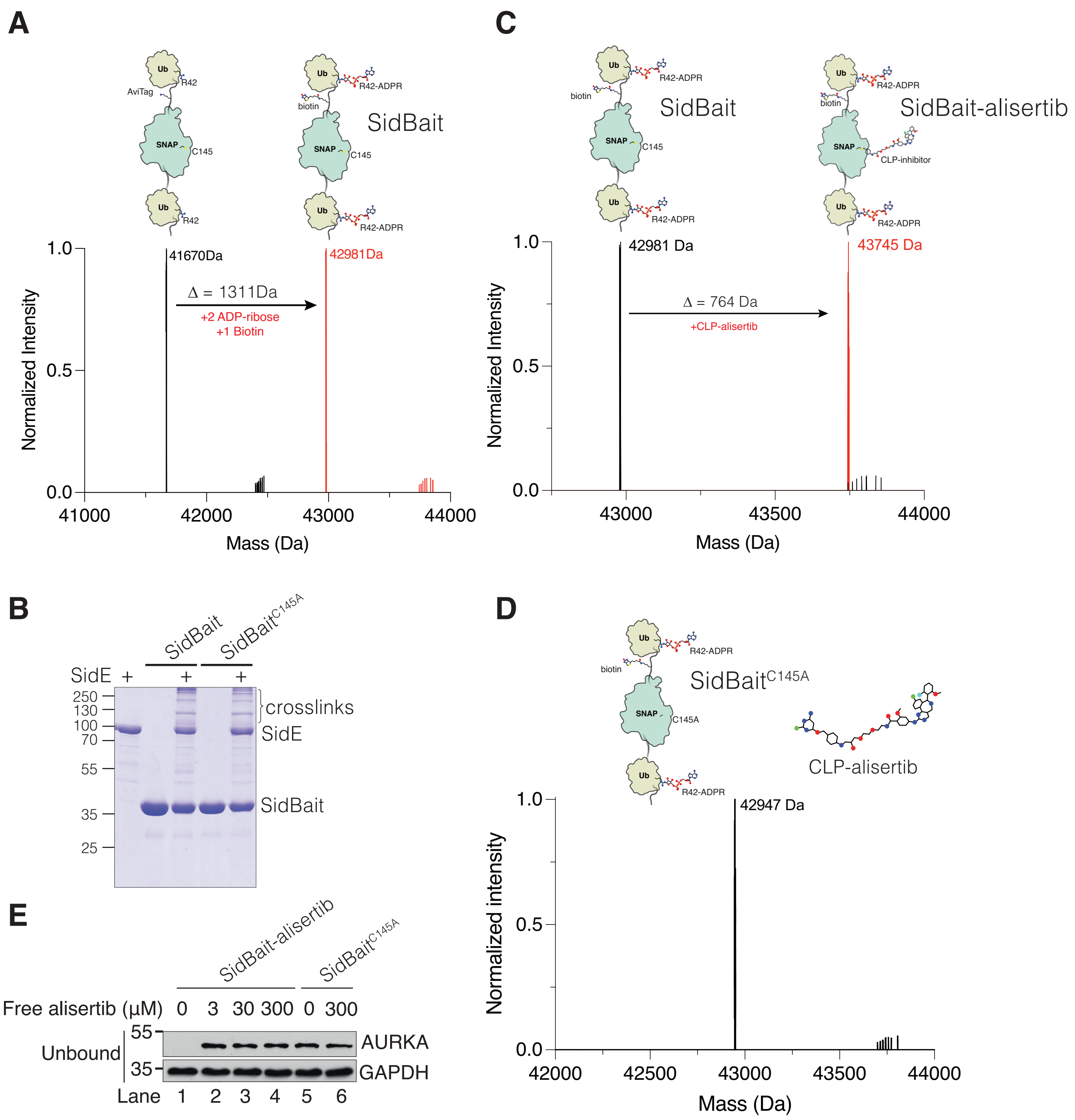

Figure EV1. SidBait identifies targets of small molecules.

(**A**) Intact mass spectrum of unmodified SidBait (left, black) and SidBait which contains a biotin and two ADP-ribose molecules (right, red). (**B**) NAD$^+$-independent SidE autoubiquitination of the SidBait probe. The SidBait probe was incubated with SidE^{PDE} and the reaction products were separated by SDS-PAGE and visualized by Coomassie staining. Cross-linking of the SidBait^{C145A} control (right), which cannot conjugate CLP-derivatives of small molecules, demonstrates an otherwise functional SidBait construct. (**C**) Intact mass spectrum of SidBait (left, black) and SidBait-alisertib (right, red). (**D**) Protein immunoblot of the unbound fractions following avidin enrichment of the SidBait-alisertib probe from HEK293 cell lysates that have been incubated with and without free alisertib. AURKA and GAPDH are shown. All cellular AURKA is bound to the SidBait-alisertib probe in the absence of free alisertib (lane 1). Following the addition of free alisertib, the SidBait-alisertib probe is competed off AURKA (lanes 2–4). (**E**) Intact mass spectrum of the SidBait^{C145A} control after incubation with CLP-alisertib, showing that the mutant protein cannot incorporate the small molecule. Source data are available online for this figure.

 © The Author(s)

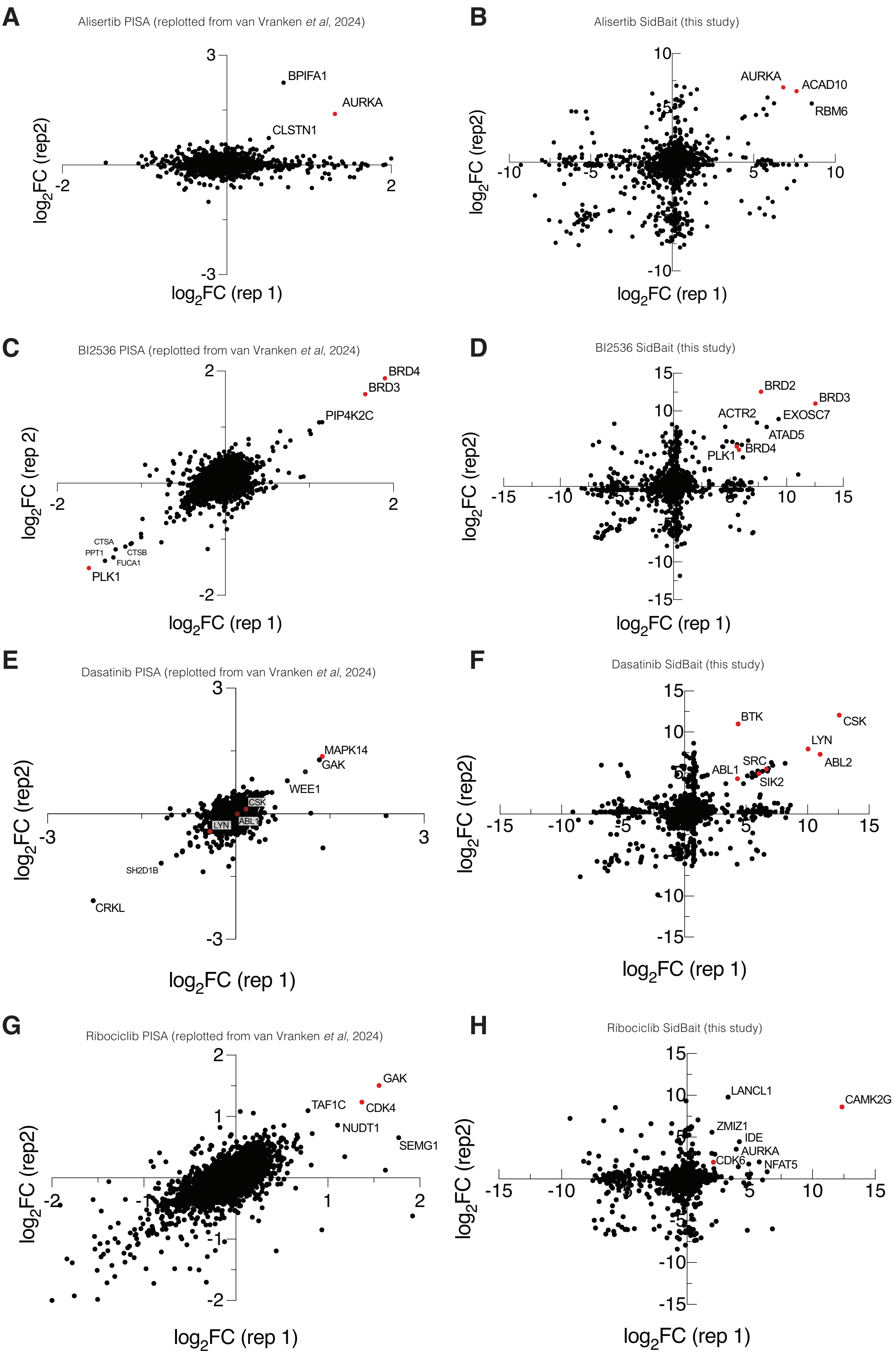
A
Alisertib PISA (replotted from van Vranken et al, 2024)
BPIFA1
AURKA
CLSTN1
log2FC (rep2)
log2FC (rep 1)
B
Alisertib SidBait (this study)
AURKA
ACAD10
RBM6
log2FC (rep2)
log2FC (rep 1)
C
BI2536 PISA (replotted from van Vranken et al, 2024)
BRD4
BRD3
PIP4K2C
CTSA
PPT1
CTSB
FUCA1
PLK1
log2FC (rep 2)
log2FC (rep 1)
D
BI2536 SidBait (this study)
BRD2
BRD3
ACTR2
EXOSC7
ATAD5
BRD4
PLK1
log2FC (rep2)
log2FC (rep 1)
E
Dasatinib PISA (replotted from van Vranken et al, 2024)
MAPK14
GAK
WEE1
CSK
ABL1
LYN
SH2D1B
CRKL
log2FC (rep2)
log2FC (rep 1)
F
Dasatinib SidBait (this study)
BTK
CSK
LYN
ABL2
SRC
ABL1
SIK2
log2FC (rep2)
log2FC (rep 1)
G
Ribociclib PISA (replotted from van Vranken et al, 2024)
GAK
TAF1C
CDK4
NUDT1
SEMG1
log2FC (rep2)
log2FC (rep 1)
H
Ribociclib SidBait (this study)
LANCL1
CAMK2G
ZMIZ1
IDE
AURKA
CDK6
NFAT5
log2FC (rep2)
log2FC (rep 1)

Figure EV2. SidBait identifies small molecule targets in K562 cell lysates.

Comparison of protein targets enriched in PISA experiments (left; van Vranken et al, 2024) with SidBait experiments (right) for alisertib (**A**, **B**); BI2536 (**C**, **D**); dasatinib (**E**, **F**); and ribociclib (**G**, **H**). Data for each SidBait panel is from two independent experiments.

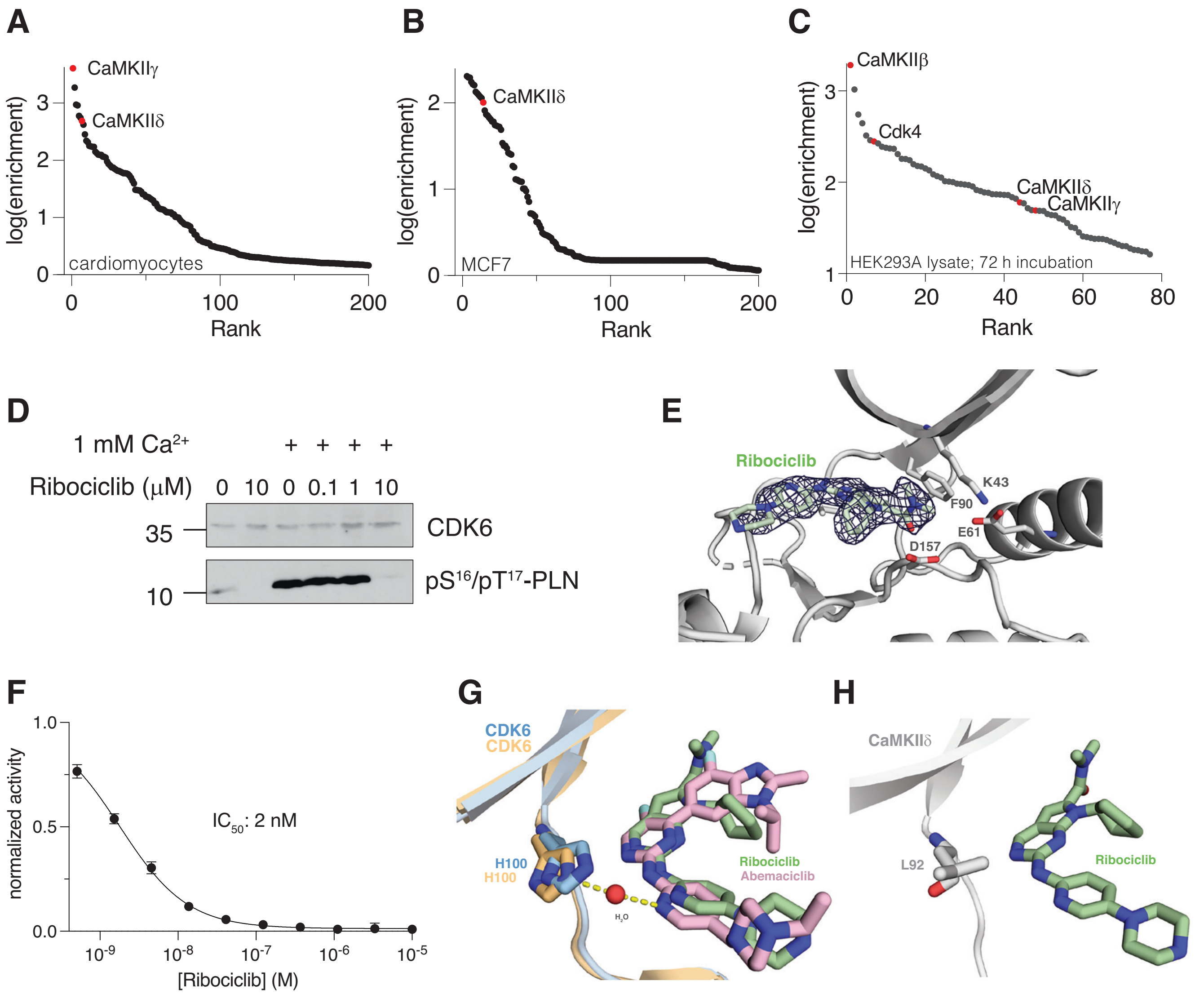

Figure EV3. SidBait identifies CaMKII as a target of the CDK4/6 inhibitor ribociclib.

(**A**, **B**) Plot of the fold enrichment of proteins from SidBait-ribociclib experiments in cultured cardiomyocytes (**A**) and MCF7 cells (**B**). (**C**) Plot of the fold enrichment of proteins from SidBait-ribociclib experiments in HEK293A cells following a 72-hour incubation with the bait. (**D**) Protein immunoblot of human cardiomyocyte lysates that have been stimulated with Ca^{2+} in the presence of varying concentrations of ribociclib. Total CDK6 is shown as a control; $pThr^{16}/pSer^{17}$-phospholamban is shown as an indication of endogenous CaMKII activity. (**E**) A view of ribociclib in the active site of the CaMKII kinase domain. The $2F_o$-F_c electron density map, contoured to 1σ, is represented by a dark blue mesh. (**F**) In vitro CDK4/cyclinD1 activity assay in the presence of varying concentrations of ribociclib. The IC_{50} of ribociclib is shown in the inset. Error bars represent the S.E.M. of three replicates. (**G**, **H**) Structural comparison between CDK6 and CAMKII bound to inhibitors. Structures of ribociclib and abemaciclib in the active site of CDK6 (**G**), showing bridging interaction through an ordered water molecule. Ribociclib in CaMKII (**H**) instead forms a contact with a backbone nitrogen through a water molecule, as seen in Fig. 2. Source data are available online for this figure.

© The Author(s)

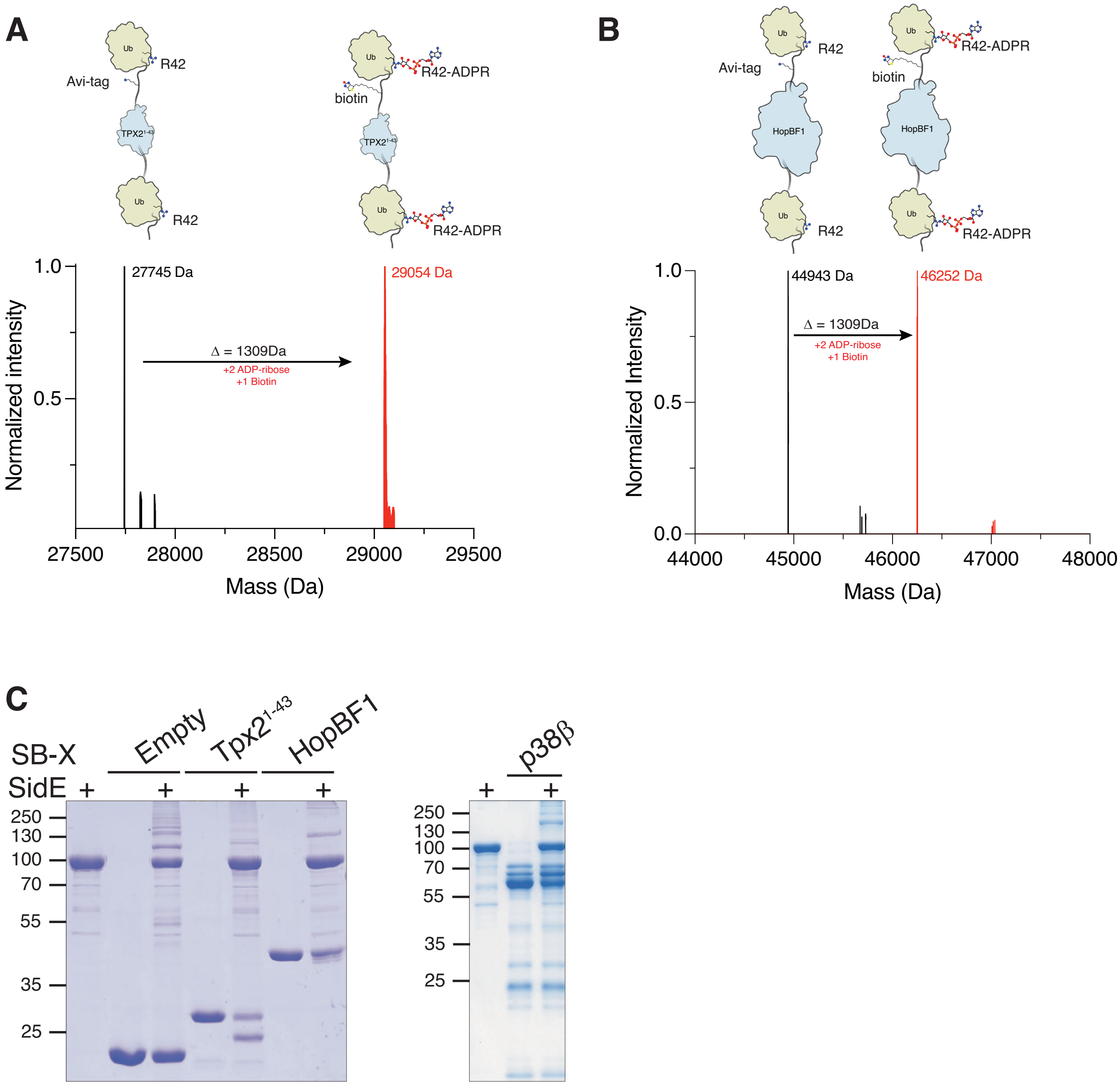

Figure EV4. SidBait identifies targets of proteins of interest.

(**A**, **B**) Intact mass spectra of unmodified SidBait-Tpx2$^{1-43}$ (**A**) and SidBait-HopBF1 (**B**) (left, black), and the respective SidBait-POI molecules containing a biotin and two ADP-ribose molecules (right, red). (**C**) NAD^{+}-independent SidE autoubiquitination of the SidBait-POI probes. The probes were incubated with SidE and the reaction products were separated by SDS-PAGE and visualized by Coomassie staining. Source data are available online for this figure.

 © The Author(s)

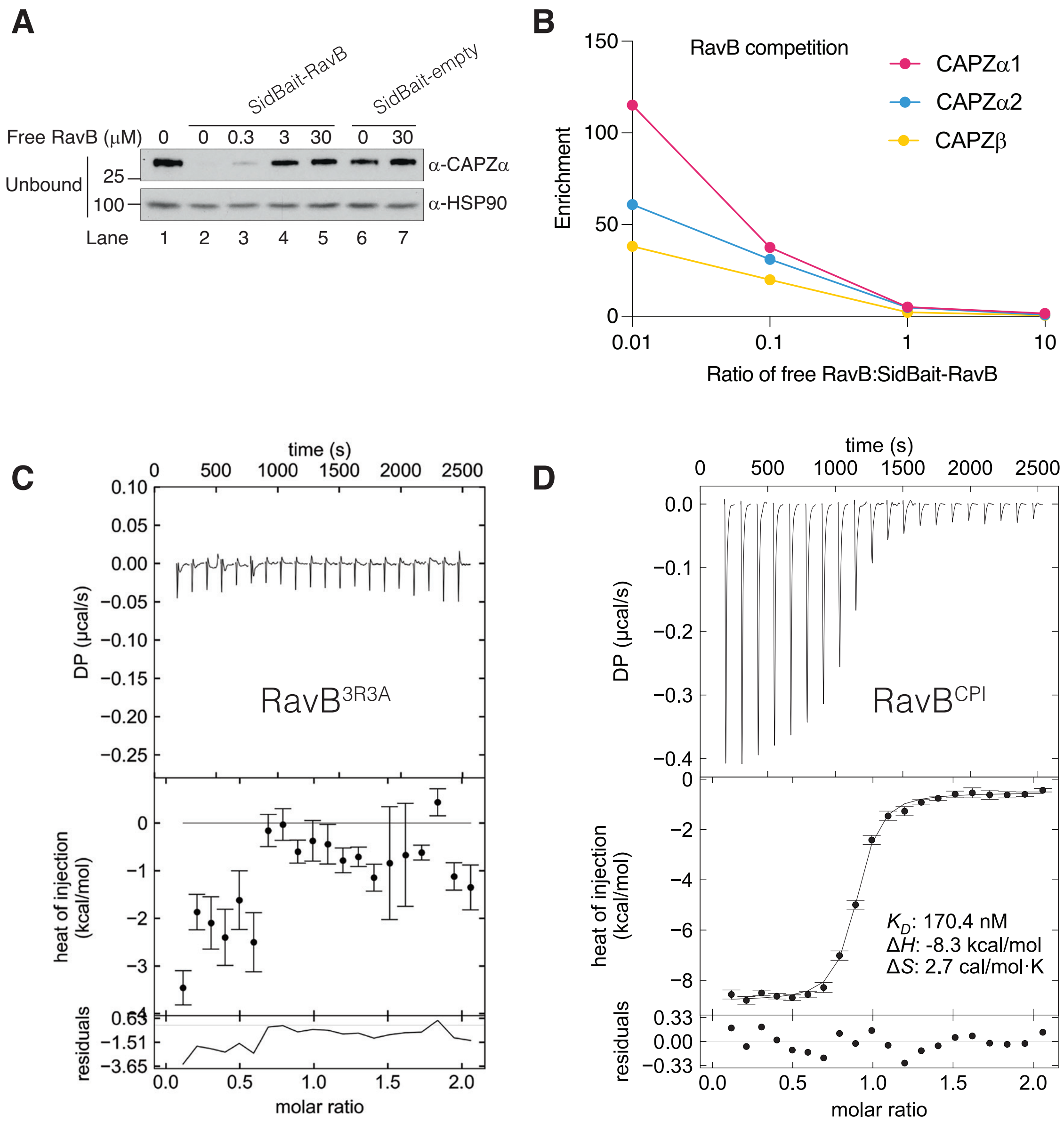

Figure EV5. SidBait identifies CapZ as a binding partner of the *Legionella* effector RavB.

(A) Protein immunoblot of the unbound fractions following avidin enrichment of the SidBait-RavB probe from HEK293 cell lysates that have been incubated with and without free RavB. CapZα and HSP90 are shown. CapZ is readily detectable in a cell lysate (lane 1). All cellular CapZ is bound to the SidBait-RavB probe in the absence of free RavB (lane 2). Following the addition of free RavB, the SidBait-RavB probe is competed off RavB (lanes 3–5). **(B)** Plots of the decreasing fold enrichment of CapZ isoforms from SidBait-RavB probe as quantified by mass spectrometry after addition of increasing competing free RavB. **(C, D)** Isothermal titration calorimetry (ITC) traces showing the binding of $RavB^{3R3A}$ **(C)** or $RavB^{108\text{-}148}$ containing the $RavB^{CPI}$ **(D)** to CapZ. In each ITC experiment, the RavB species was injected into the cell containing CapZ. K_d, enthalpy and entropy values are shown in the inset. These values are undefined for $RavB^{3R3A}$, as no binding was observed. Source data are available online for this figure.

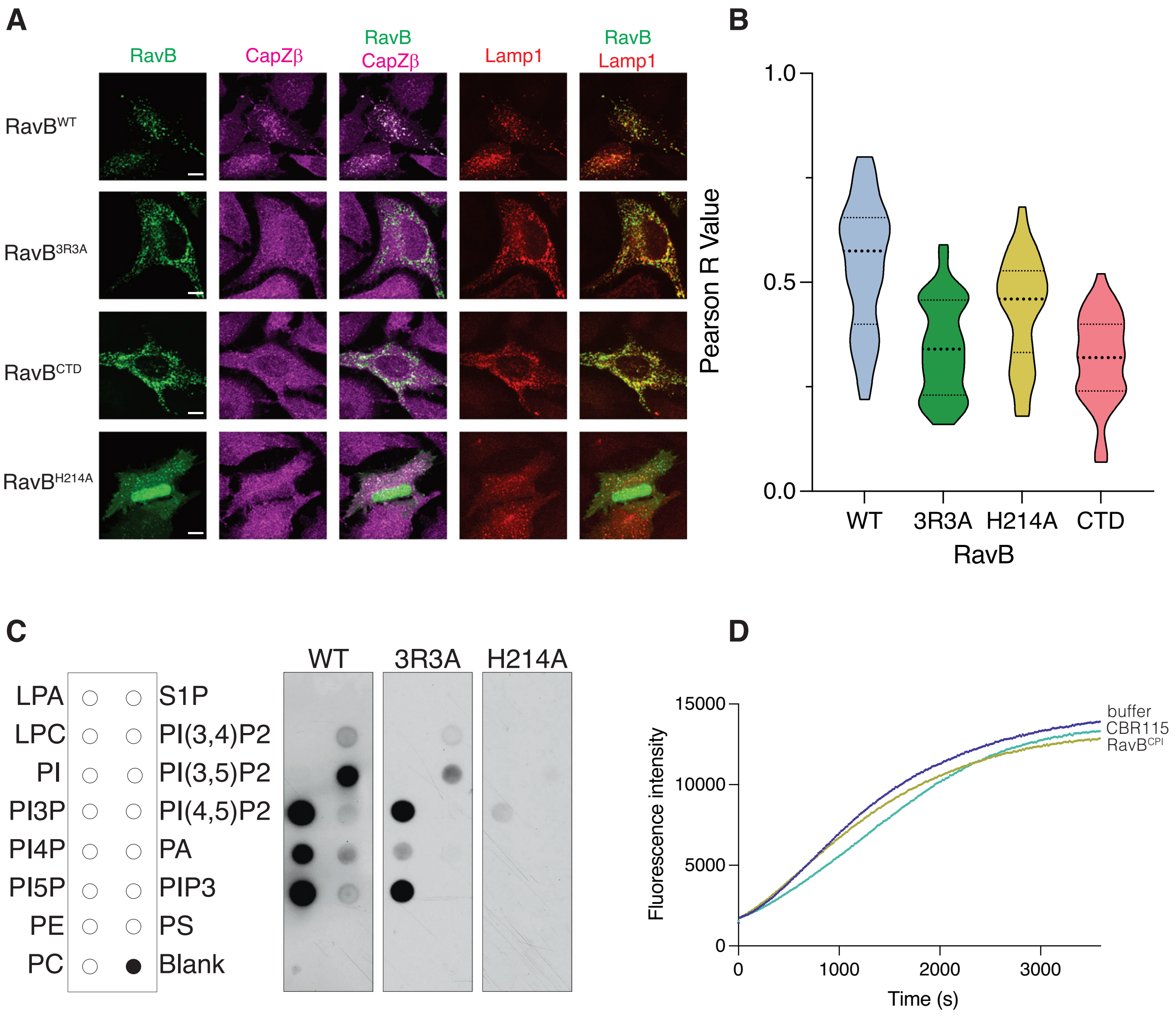

Figure EV6. RavB is a phosphatidylinositol phosphate binding, actin decapping protein.

(**A**) Immunofluorescence microscopy of HeLa cells expressing EGFP-RavB^{WT} or various mutants, mTagBFP-Lamp1. Endogenous CapZβ is also shown (magenta). The images depict the entire cell from Fig. 5a. Scale bar represents 10 µm. (**B**) Plot of the Pearson R Value of colocalization between transfected mTagBFP-Lamp1 and endogenous CapZβ in cells expressing WT or various RavB mutants. Each comparison was calculated using 40 cells across 3 independent experiments. (**C**) Protein immunoblot of RavB or mutants bound to a lipid panel spotted on PIP strips with an anti-RavB antibody. All membranes shown were exposed on the same film. (**D**) Pyrene-actin polymerization assays demonstrating that the RavB^{CPI} does not affect actin polymerization. The polymerization of actin was measured in the presence of a buffer control (blue), the known decapping peptide CBR115 (teal) and the RavB^{CPI} peptide (green). Assays were run with 2 µM G-actin and 1.25 µM decapping peptide. Source data are available online for this figure.

 © The Author(s)

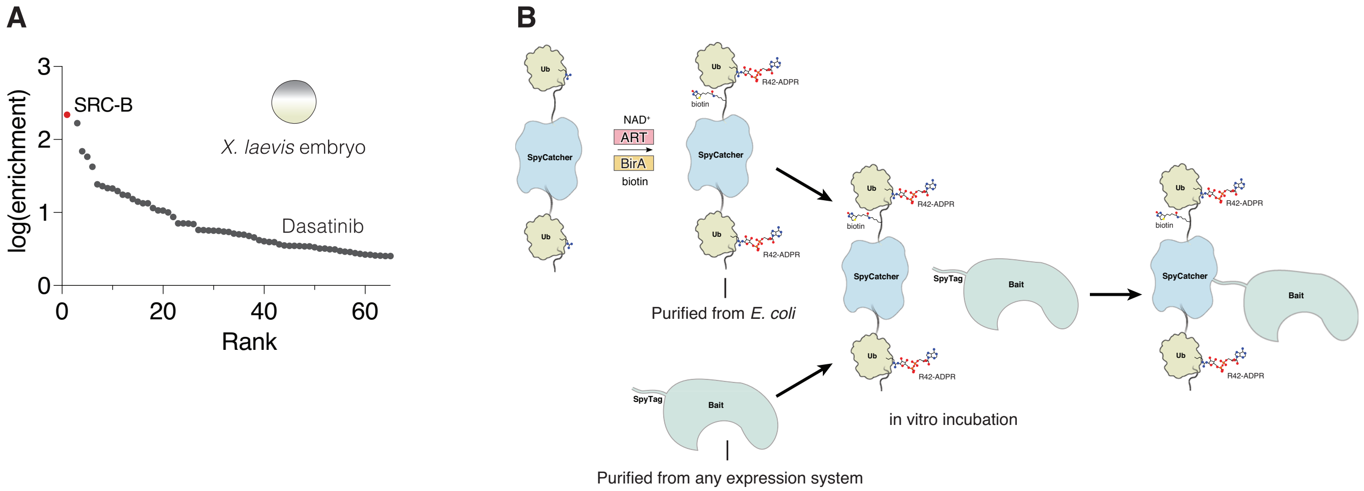

Figure EV7. Additional applications of SidBait.

(**A**) Plot of fold enrichment of proteins from SidBait-dasatinib in live *X. laevis* embryos over pulldowns with the SidBait^{C145A} control. (**B**) Schematic of the SpyTag/SpyCatcher system as applied to SidBait. The Ub-SpyCatcher-Ub fusion protein is co-expressed in *E. coli* with the SidE ART domain and BirA. The ADP-ribosylated and biotinylated protein is incubated with a protein of interest (POI) fused to the SpyTag, which has been purified from an alternative expression system. Upon mixing, the SpyTag-POI spontaneously conjugates to the SpyCatcher protein, forming a stable isopeptide bond. The resulting fusion protein is used in a SidBait experiment.

© The Author(s)
